# Metabolic**/**Proteomic Signature Defines Two Glioblastoma Subtypes With Different Clinical Outcome

**DOI:** 10.1038/srep21557

**Published:** 2016-02-09

**Authors:** G. Marziali, M. Signore, M. Buccarelli, S. Grande, A. Palma, M. Biffoni, A. Rosi, Q.G. D’Alessandris, M. Martini, L. M. Larocca, R. De Maria, R. Pallini, L. Ricci-Vitiani

**Affiliations:** 1 Department of Hematology, Oncology and Molecular Medicine, Istituto Superiore di Sanità, Rome, Italy; 2 Department of Technology and Health, Istituto Superiore di Sanità, Rome, Italy; 3 Institute of Neurosurgery, Università Cattolica del Sacro Cuore, Rome, Italy; 4 Institute of Anatomic Pathology, Università Cattolica del Sacro Cuore, Rome, Italy; 5 Regina Elena National Cancer Institute, Rome, Italy

## Abstract

Glioblastoma (GBM) is one of the deadliest human cancers. Because of the extremely unfavorable prognosis of GBM, it is important to develop more effective diagnostic and therapeutic strategies based on biologically and clinically relevant subclassification systems. Analyzing a collection of seventeen patient-derived glioblastoma stem-like cells (GSCs) by gene expression profiling, NMR spectroscopy and signal transduction pathway activation, we identified two GSC clusters, one characterized by a pro-neural-like phenotype and the other showing a mesenchymal-like phenotype. Evaluating the levels of proteins differentially expressed by the two GSC clusters in the TCGA GBM sample collection, we found that SRC activation is associated with a GBM subgroup showing better prognosis whereas activation of RPS6, an effector of mTOR pathway, identifies a subgroup with a worse prognosis. The two clusters are also differentiated by NMR spectroscopy profiles suggesting a potential prognostic stratification based on metabolic evaluation. Our data show that the metabolic/proteomic profile of GSCs is informative of the genomic/proteomic GBM landscape, which differs among tumor subtypes and is associated with clinical outcome.

Glioblastoma multiforme (GBM) represents the most common and malignant brain tumor in adults, characterized by a high degree of cellular and genetic heterogeneity^1^. The overall prognosis of GBM patients remains poor with a median survival of 12–15 months^2^ despite multimodal therapy, including neurosurgical resection and radiotherapy with concomitant and adjuvant alkylating agent temozolomide. The clinical hallmarks of GBM that contribute to its awful prognosis are aggressive growth, limited response to therapy, and inexorable recurrence.

The emergence of molecularly focused approaches to cancer has fundamentally changed the path to diagnosis and treatment of malignancies. Histology is increasingly being supplemented with molecular analyses and these data subsequently inform therapeutic decision-making^3^.

In the framework of the The Cancer Genome Atlas (TCGA) a large panel of GBM samples have been analyzed at the combined genetic, epigenetic and proteomic level leading to the characterization of core tumorigenic pathways, identification of novel genes associated with the pathogenesis of GBM and classification into distinct, clinically relevant molecular subtypes^4^,^5^,^6^,^7^,^8^,^9^. Genomic profiling defined four subtypes of GBM^8^, which were named based on the expression of signature genes as, i) proneural, highly enriched with the oligodendrocytic signature but not with the astrocytic signature; ii) neural, associated with oligodendrocytic and astrocytic differentiation and additionally enriched for genes expressed by neurons; iii) classical, strongly associated with the murine astrocytic signature; iv) mesenchymal, associated with the expression of mesenchymal and astrocytic markers^4^,^10^. Single cell RNA-seq shows that GBM assigned to a subtype based on tumor bulk analysis, present heterogeneous mixtures with individual cells corresponding to different glioblastoma subtypes and that the presence of heterogeneity of subtypes at the single cell level influences clinical outcome^11^.

Altogether these data show that “glioblastoma” is a heterogeneous collection of distinct diseases, with multiple pathway-dependencies both within and across each particular subtype.

Growing evidences confirm that GBM contains a subpopulation of cells displaying stem-like properties reminiscent of normal stem cells, called tumor-initiating cells or GBM stem-like cells (GSCs) that are believed to play a fundamental role in tumor resistance to chemo- or radiotherapy and in recurrence^12^. GSCs can be isolated to generate cell lines characterized by self-renewing, multipotency, and highly tumorigenic ability and are reported to mirror both the genomic and the gene expression profiles of the original tumor more closely than conventional serum-cultured glioma cell lines^13^,^14^. Although the functional criteria defining GSCs are widely accepted, the molecular characteristics of these cells have not been fully identified^12^. As expected from the heterogeneous histology of GBM, there is extensive cellular heterogeneity within GSCs as well^11^. The complex interplay of signaling pathways and the lack of common molecular markers identifying GSCs further complicate the analysis of these cells.

To further dissect the molecular biology of GBM and seeking for appropriate clinical targets to be exploited for drug treatment, we analyzed our collection of patient-derived GSCs by combining complementary molecular approaches. Taking into account the most variable genes/transcripts, gene expression profiling of GSCs revealed two distinct clusters. These clusters closely overlapped those obtained both from metabolic analysis by NMR spectroscopy and from signal transduction pathway activation, as assessed by reverse-phase protein microarray technology (RPPA). In addition, we analyzed available RPPA data from TCGA to evaluate the capability of proteins differentially expressed in the two GSC clusters to predict patient survival.

## Results

### Gene expression profiling identifies two distinct phenotypes of GSC lines

Tumor samples from 55 GBM patients were mechanically dissociated and cultured in stem cell medium. Seventeen of the 55 GBMs (31%) generated GSC lines. There were no significant differences between GSC-generating tumors and those tumors that did not generate GSC cultures relative to patient age and sex, symptom duration, and tumor location. Notably, median overall and progression free survival were significantly shorter in tumors that generated GSC cultures as compared with those that did not^15^. GSC lines were expanded and previously validated for their stem cell properties, such as the ability to self-renew, to generate progeny of multiple lineages in differentiating culture conditions and to phenocopy the patient tumor in mouse xenografts. In addition they were characterized for the expression of stem cell markers, including CD133 and SOX2, and for the presence of tumor-specific genetic changes^15^.

**Figure 1:**
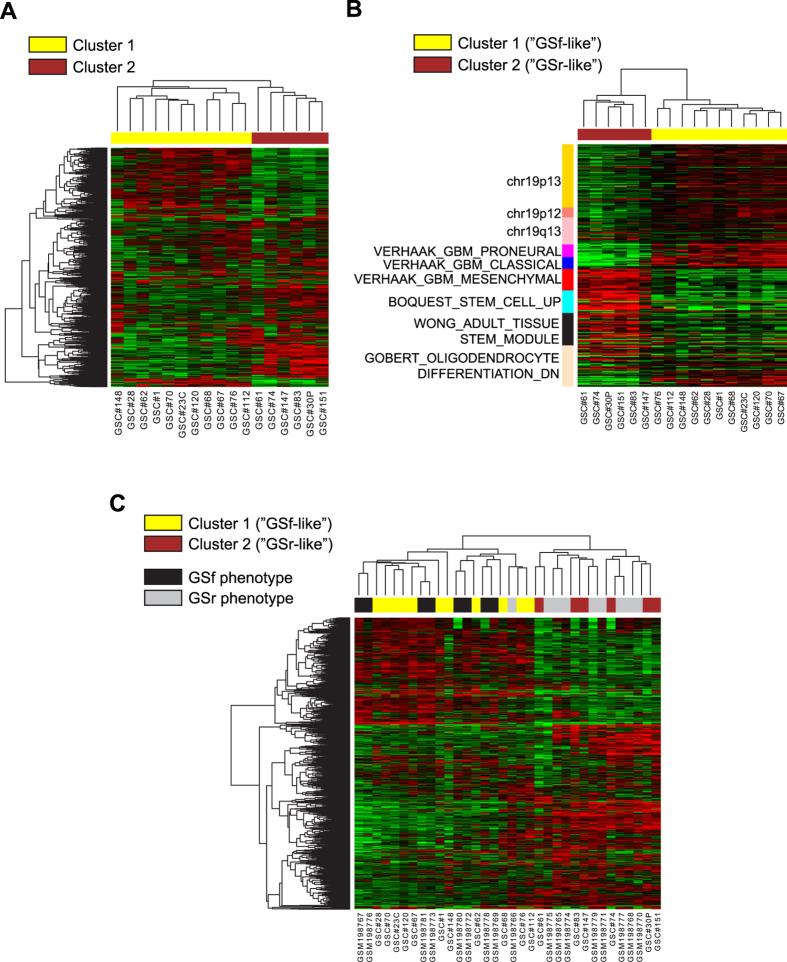
Gene expression analysis of 17 patient-derived GSCs identifies two distinct clusters. (**A**) Hierarchical clustering of GSCs using the top 1,000 most variable genes/probes in our gene expression datasets. (**B**) Hierarchical clustering of GSCs using a subset of the genes/probes in A, selected among the highest-score Gene Sets resulting from GSEA on genes/probes differentially regulated between Cluster 1 and Cluster 2. The colored left-side bar annotates the correspondent Gene Sets clusters. (**C**) Hierarchical clustering of the top 1,000 most variable common genes found in our GSC (yellow and brown rectangles) samples and in Schulte GSr/GSf (grey and black rectangles) samples. Heatmaps throughout the figure display differences in expression levels in log2 scale (red + 3, black = 0, green = −3).

Gene expression data were collected from all the GSC lines and unsupervised hierarchical clustering of the samples using the 1,000 most variable genes/transcripts (below and above the interquartile range of the full dataset) produced two distinct GSC clusters (Fig. 1A), reminiscent of the separation into GSf (full stem) and GSr (restricted stem) phenotypes previously reported by Günther and colleagues, and by Schulte and colleagues^16^,^17^. The GSf phenotype is characterized by proneural-like gene expression signature, growth as floating spheres *in vitro*, CD133 expression, and high invasiveness *in vivo*. Conversely, GSCs with a GSr phenotype show mesenchymal-like expression signature, no CD133 expression, *in vitro* adherent growth, and low invasive behavior *in vivo*^16^.

The 470 genes differentially regulated between our two GSC clusters (“GSC signature”, Supplementary Table S1a) were then subjected to a Gene Set Enrichment Analysis (GSEA)^18^,^19^. Chromosomal loci 19p13, 19p12 and, to a somewhat lesser extent, 19q13 were highly enriched in the GSC signature (Supplementary Table S1b). All these regions contain a large number of Krüppel-Type Zinc Finger proteins^20^. Region 19q13 has been reported as hypermethylated in oropharyngeal squamous cell carcinoma^21^ and copy-number-gain has been described in GBM^22^. The GSEA analysis also showed partial overlaps with mesenchymal, proneural and classical GBM sub-signatures established by Verhaak and colleagues^8^, an adult tissue stem-cell signature^23^, a stromal stem cell signature^24^ and oligodendrocyte differentiation^25^ (Supplementary Table S1c). Clustering of the GSC expression data using these partial gene lists (Fig. 1B) indicates a higher degree of “stemness” in the GSr-like lines coupled to a more mesenchymal-like phenotype. On the contrary GSf-like lines showed a more proneural/classical-like phenotype with reduced neural differentiation features and higher expression of regions 19p12 and 19p13. In line with this observation, proneural GBM has been recently proposed to arise from a non-stem-cell precursor, in contrast to the other three GBM subtypes^26^,^27^.

To further characterize our samples and compare them to previously described signatures, a large unified dataset was generated including data from both the Schulte GSf/GSr^17^ and the Verhaak TCGA GBM studies^8^. Genes/probes from the different microarray platforms were integrated through a batch correction procedure using only two global sample types, stem-cell or bulk tumor, i.e. without explicitly assigning a GSf or GSr phenotype to any sample. In the unified dataset, the GSC and GSf/GSr samples reproduce two homogeneous clusters with GSf(-like) or GSr(-like) phenotype, respectively (Fig. 1C), thus confirming the initial phenotype assignment of the GSC clones. Extending the analysis to include also the four subtypes of TCGA GBM samples and clustering by the Verhaak GBM subtype signatures, the GSr/GSr-like and GSf/GSf-like samples distinctly co-clustered with either the mesenchymal (M) or proneural (P)/classical (C) tumor subtypes, respectively (Supplementary Fig. S1a). Moreover, the overlap between the top 1000 most variable genes in GSf- *vs* GSr-like and the top 1000 most variable genes in GSf/GSr combined dataset, showed that 609 genes are shared between GSf-/GSr-like and GSf/GSr (Supplementary Fig. S1b and Supplementary Table S1d). Interestingly, RNAseq analysis of 447 cancer-related genes on 167 TCGA GBM samples, shows a less stringent division between known subgroups when compared to the complete microarray panel (Supplementary Fig. S2).

### ^1^H NMR spectroscopy reveals two distinct metabolic phenotypes of GSCs

To identify possible metabolic phenotypes, the 17 GSC lines were assessed by 1D^1^H NMR spectroscopy. NMR spectra were acquired seven days after plating and were analyzed as previously described^28^ for the following metabolites: N-acetylaspartate (NAA), a neural marker involved in neurotransmitter production; γ-aminobutyric acid (GABA), glutamate (Glu) and aspartate (Asp), which are major inhibitory or excitatory neurotransmitters in the brain; mobile lipids (MLs), often associated with cell death typical in brain malignancies^29^; myoinositol (Myo-I), related with the grade of brain tumors^30^; glutamine (Gln), related to the glial phenotype, and glutathione (GSH), an antioxidant regulator of ROS level^31^.

Clearly detectable signals of the astrocytic marker Myo-I and Gln were present in all GSC spectra, although with different signal intensities.

**Figure 2:**
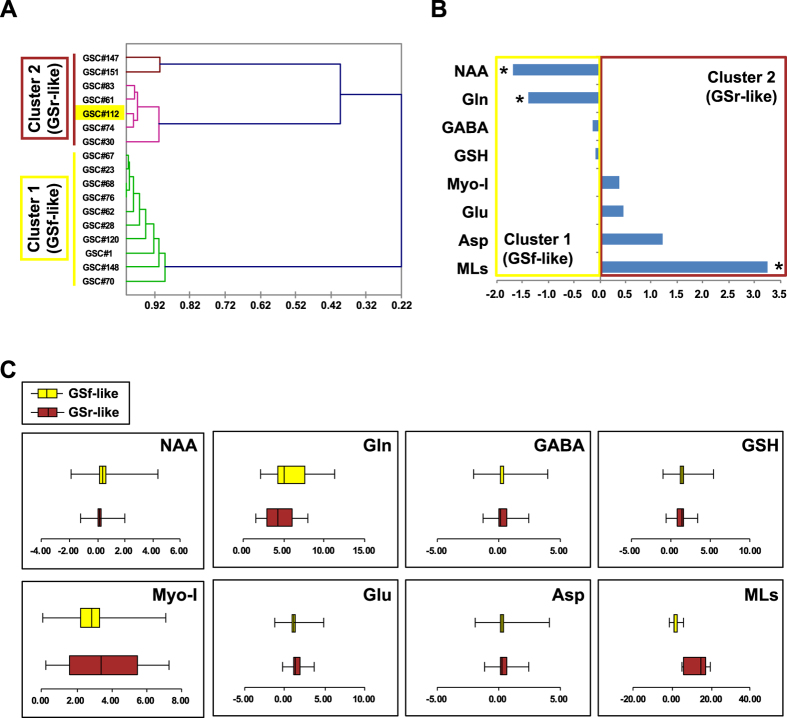
1D^1^H NMR distinguishes two metabolic profiles of GSCs. (**A**) Unsupervised clustering of GSCs based on the levels of the analyzed metabolites. Samples are annotated with their matching gene expression clusters (colored boxes on the left) with the exception of sample #112. (**B**) GSr-/GSf-like ratios of NMR signal intensity means were converted to log2 and expressed as fold change (FC). Metabolites displaying a statistically significant (p < 0.05, Student’s t-test) difference between GSr- and GSf-like are indicated by asterisks. (**C)** Boxplots of raw NMR signal intensities of metabolites measured in GSf-like (cluster 1, yellow) and GSr-like (cluster 2, brown) samples.

Unsupervised cluster analysis on the 17 GSC lines resulted in a dendrogram clearly separating the samples into two clusters (Fig. 2A). The first cluster, which is characterized by low lipids and high NAA and Gln, includes all GSf-like lines except the GSC line #112, whereas all GSr-like lines are grouped in the second cluster, characterized by high lipids, low Gln and absent NAA. NAA signals were clearly observed only in GSf-like lines, indicating a metabolic fingerprint typical of neurons (Fig. 2B). These signals were barely detectable in GSr-like spectra, thus suggesting that the neuronal metabolism, although present, is not prevalent in these lines. The intensity of MLs signal was higher in GSr-like line spectra compared to GSf-like ones (Fig. 2B,C), indicating a prevalent astrocyte/glioma-like metabolism. These two clusters closely superimpose those defined by gene expression analysis with the only exception of GSC line #112 (Fig. 2A), supporting an association of the GSf-like lines with the proneural subtype and of the GSr-like ones with the mesenchymal subtype.

### Phosphoproteomic analysis confirms differential metabolic profiles in GSf-like and GSr-like cells

**Figure 3:**
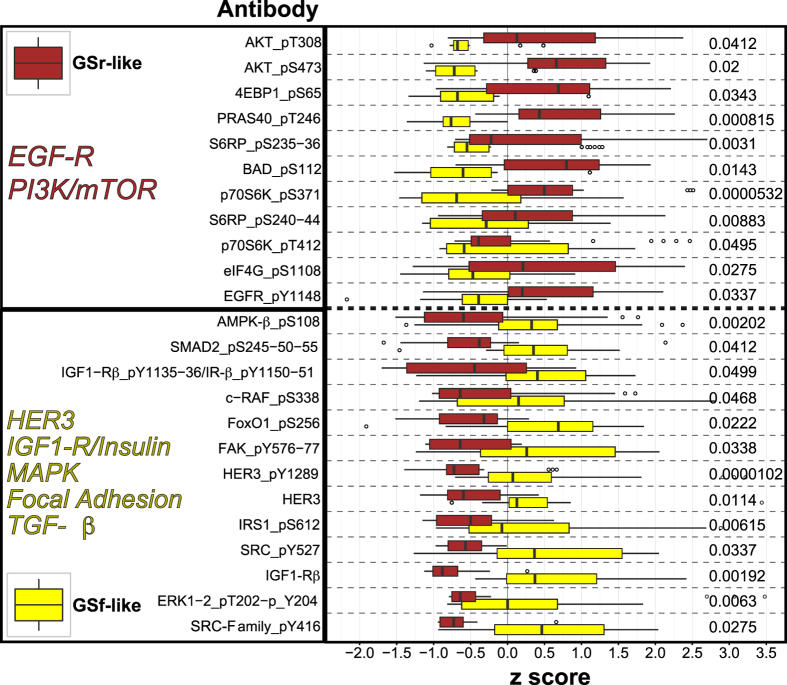
Phosphoproteomic analysis supports genomic and metabolic clustering of GSCs. Boxplots of standardized levels of selected RPPA analytes displaying a statistically significant (p < 0.05, Wilcoxon rank sum test) difference between GSCs of GSr- or GSf-like gene expression cluster. Based on the absolute difference between RPPA levels measured in GSr- and GSf-like GSCs, endpoints were divided in two groups, i.e. GSr-like (brown, high in GSr-like) and GSf-like (yellow, high in GSf-like), respectively. RPPA endpoints are sorted in descending order starting from the one showing the highest levels in GSr-like GSCs. Each antibody is annotated (right side) with the corresponding p value. Outliers are shown as circles and names of the major pathways defining GSr- or GSf-like cells are reported for both clusters.

Genomic and transcriptomic analyses cannot fully account for the translational and post-translational integration of the complex genomic and transcriptomic aberrations accumulated in each tumor^32^,^33^,^34^. We recently demonstrated that reverse-phase protein array (RPPA) analysis identifies pathway activation patterns that correlate with the response of GSCs to kinase inhibitors^35^. Therefore, we performed RPPA analysis on our collection of GSCs to measure the basal state levels of total and/or phosphorylated forms of diverse proteins involved in regulation of stemness, differentiation, migration, cell cycle, DNA damage, proliferation, cell growth, stress response and apoptosis (Supplementary Table S2). A comprehensive graphical representation and a list of the results of RPPA analysis on our GSC lines is available as Supplementary Fig. S3 and Supplementary Table S3, respectively. A pathway-oriented rather than single-endpoint approach to signaling analysis showed that GSf-like cells have a significant increase in SRC, Mitogen Activated Protein Kinase (MAPK), and Insulin-like Growth Factor- Receptor (IGF1-R/IR), whereas GSr-like lines displayed increased levels of phosphorylated proteins associated with the mammalian Target of Rapamycin (mTOR) pathway (Fig. 3).

### GSf- and GSr-like classification and clinical correlations

In a previous study, we demonstrated that the efficiency in establishing a GSC culture is about 30–40%, and that the capacity to generate GSCs represents an independent prognostic factor for poor patient survival^15^. Thus, we explored possible associations of the GSC subtypes, i.e. GSf-like and GSr-like, with clinical and pathological parameters of the donor patients. Symptom duration prior to diagnosis was significantly shorter, indicative of a more aggressive clinical behaviour, in tumors that generated GSr-like cultures as compared with tumors generating GSf-like cultures (*p* = 0.0422; Mann-Whithey *U* test). In addition, a trend towards a worse general status, as assessed by the Karnofsky Performance Status score (KPS), was evident for tumors generating GSr-like cultures (*p* = 0.0529; Mann-Whithey *U* test). The median overall survival of the donor patients was 10.5 and 8 months for GSf-like and GSr-like tumors, respectively. No significant differences were found between GSf- and GSr-like generating tumors in terms of patient age or sex, tumor location or diameter, and extent of tumor resection (Supplementary Table S4). Among the molecular variables, EGFRvIII expression was significantly more frequent in tumors generating GSr-like cultures than in those generating GSf-like cultures (*p* = 0.0345; Fisher exact test; Supplementary Table S5).

### Analysis of GSf- and GSr-like molecular signatures in TCGA data

**Figure 4:**
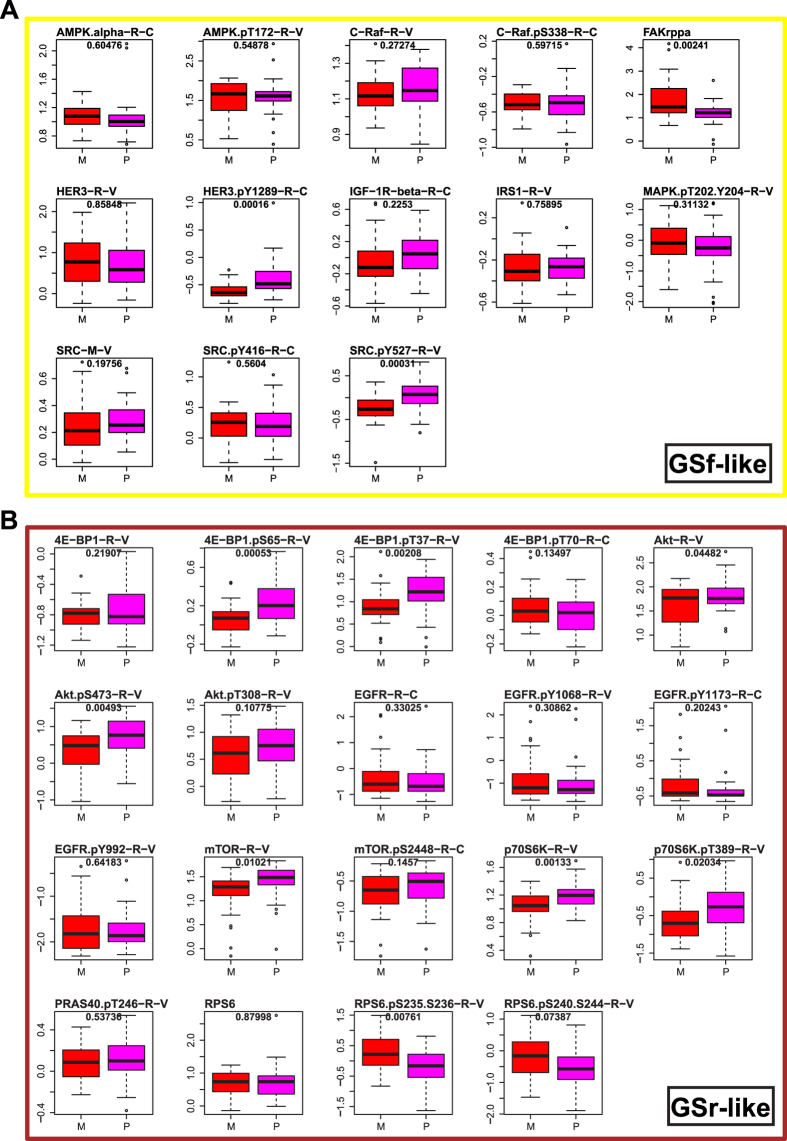
RPPA data from TCGA GBM samples. Boxplots of standardized levels of RPPA analytes measured in P (proneural, n = 41, pink) and M (mesemchymal, n = 29, red) samples selected from the full cohort (n = 214) of GBM tumors that was subjected to RPPA analysis within the TCGA consortium. The RPPA endpoints reported here were selected based on the presence, in the TCGA data, of analytes matching the GSf- (**A**, yellow box), or GSr-like (**B**, brown box) lists defined by our RPPA analysis (see methods sections for details). P values from unpaired t test are reported on top of each endpoint plot.

**Figure 5:**
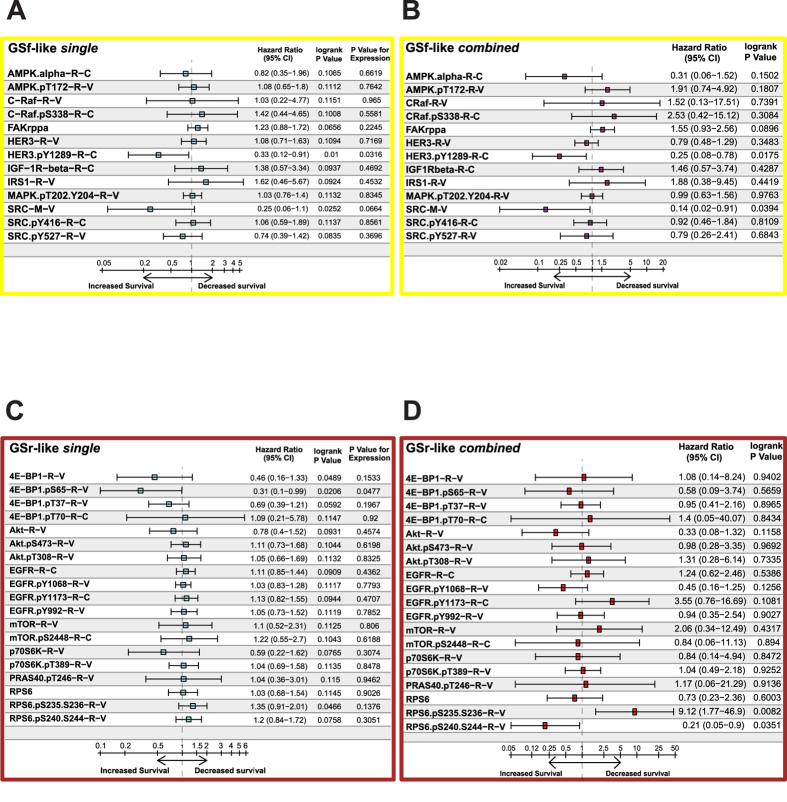
GSf- and GSr-like RPPA endpoints ability to predict TCGA patient survival. Table plots of hazard ratios (HR) for expression levels of individual RPPA analytes, selected as distinctive of GSf- (**A**) and GSr-like (**B**) groups. For each RPPA endpoint, a Cox regression model was applied using the intensity levels (z score) and the subtyping as covariates. In order to generate a multi-gene prognostic index (mPI), the full selection of either GSf- (**C**) or GSr-like (**D**) RPPA analytes were included as covariates in a Cox model and table plots of corresponding hazard ratios are shown. Confidence intervals (95% CI) are reported for all HR together with logrank test p values and, for **A** and **B**, p values for the RPPA levels. The cohort of patients used for performing Cox survival analyses includes all TCGA patients with known Verhaak subtype (n = 111).

The most comprehensive (phospho-)proteomic characterization of GBM has recently been done under TCGA initiative, by Brennan and colleagues using RPPA^9^. We explored such data, comprising 181 protein analytes on 251 GBM samples, and found that unsupervised clustering failed to produce a consistent partitioning of the sample cohort into the four clearly-defined gene expression subtypes described by Verhaak and colleagues^8^, but rather grouped them into two dominant GBM subtypes (Supplementary Fig. S4). Thus, we asked whether the significantly different endpoints identified in GSf- and GSr-like GSCs by our RPPA analysis, could have prognostic value in the large cohort of GBM samples from TCGA. To this end, we used the Glioblastoma Bio Discovery Portal (GBM-BioDP, see methods section for details) and, as a query input, two gene symbol lists based on RPPA endpoints that were differentially expressed between GSf- and GSr-like GSCs (Supplementary Table S6 and methods section). Focusing on the proteins characterizing the GSf-like subgroup, we found that the levels of activated Human Epidermal Growth Factor Receptor 3 (HER3 pY1289) were higher in P *versus* M subtype (*p* = 0.00016, Fig. 4A) confirming a relationship between P and GSf subtypes. In addition, HER3 pY1289 was associated with longer overall survival in the global population (*p* = 0.01; Cox log rank test) (Fig. 5A and Supplementary Table S7). Phosphorylation of SRC at Y527 (SRC pY527) was significantly higher in P than in M GBM subtypes (*p* = 0.00031), whereas SRC pY416 was not different (Fig. 4A and Supplementary Table S7). Interestingly, despite a significant overall decrease in the levels of Focal Adhesion Kinase (FAK) in P *versus* M subtype (*p* = 0.00241), apparently challenging our RPPA data (Fig. 4A), FAK expression in TCGA data is significantly associated with poor prognosis only in P subtype (*p* = 0.0243; Cox log rank test, Supplementary Fig. S5a and Supplementary Table S7).

After single-endpoint analyses, we exploited the built-in feature of the GBM-BioDP platform to generate a multi-gene prognostic index (mPI) from RPPA data, including all GSf-like endpoints as covariates in a single Cox regression model (Fig. 5B). The resulting mPI (mPI_GSf_) was significantly associated with the outcome in both P and M subtypes (*p* = 0.000035 for the P subgroup and *p* = 0.000273 for the M subgroup; Cox log-rank test, Supplementary Fig. S6a), and also with the entire patient population (*p* = 0.000558; Cox log-rank test, Supplementary Fig. S6a) suggesting its potential unrestricted clinical relevance. Notably, levels of HER3 pY1289 and SRC resulted as significant covariates (HR = 0.25, *p* = 0.0175 and HR = 0.14, *p* = 0.0394, respectively; Cox log-rank test), while FAK expression did not reach statistical significance (HR = 1.55, *p* = 0.0896; Cox log-rank test, Fig. 5B). Therefore, we used these three endpoints to build up a simplified mPI_GSf_ model which, despite a lack of power in predicting outcome in the M subtype, was capable of predicting overall survival in the overall population (*p* = 0.002786; Cox log-rank test) and in the P subtype (p = 0.000207; Cox log-rank test, Supplementary Table S8 and Supplementary Fig. S7a).

Analyzing GBM TCGA patients using single GSr-like specific endpoints, we observed significantly higher levels of phosphorylated Ribosomal Protein S6 (RPS6 pS235-36) in M *versus* P (*p* = 0.00761, Fig. 4B). Survival analysis showed that high RPS6 pS235-36 had a negative impact on survival, specifically in the P subtype (*p* = 0.0428; Cox log-rank test, Supplementary Fig. S5b and Supplementary Table S7). Notably, high levels of total and phosphorylated Eukaryotic initiation factor 4E binding protein 1 (4EBP1 and 4EBP1 pS65) were significantly associated with a better prognosis in the overall population of TCGA patients (*p* = 0.0489 and *p* = 0.0206, respectively; Cox log-rank test, Fig. 5C). High levels of 4EBP1 pS65 and two distinct phosphorylated forms of Epidermal Growth Factor Receptor (EGF-R pY1173 and EGF-R pY992) correlated with a better prognosis only in the P subgroup, whereas high AKT pS473 was markedly associated with poor survival in the same subtype (Supplementary Fig. S5b and Supplementary Table S7).

Similarly to the GSf-like signature, to define a GSr-like mPI (mPI_GSr_), we computed a single Cox regression model with all the GSr-like-specific endpoints as covariates (Fig. 5D). Interestingly, we found that levels of RPS6 pS235-36 inversely correlated with patient survival (HR = 9.12, p = 0.0082; Cox analysis, Fig. 5D) and that an increased overall survival was significantly predicted for all patients by the mPI_GSr_ (*p* = 0.002019; Cox log-rank test, Supplementary Fig. S6b). Addition of age and MGMT status as covariates, significantly increased the capacity of the mPI_GSr_ to predict a worst outcome (*p* = 0.001965; Cox log-rank test, Supplementary Fig. 7B). In particular, the mPI_GSr_ HR for the P subtype were 5.7 (*p* = 0.000073; Cox log-rank test) and 13.43 (*p* = 0.026878; Cox log-rank test) without and with age and MGMT status inclusion, respectively (Supplementary Fig. S6b and Supplementary Fig. S7b). Altogether these results indicate that the activation of two GSf-like endpoints, HER3 and SRC, is associated with a GBM subgroup showing better prognosis whereas activation of RPS6, effector of mTOR pathway and GSr-like endpoint, identifies a subgroup with a worse prognosis.

**Figure 6:**
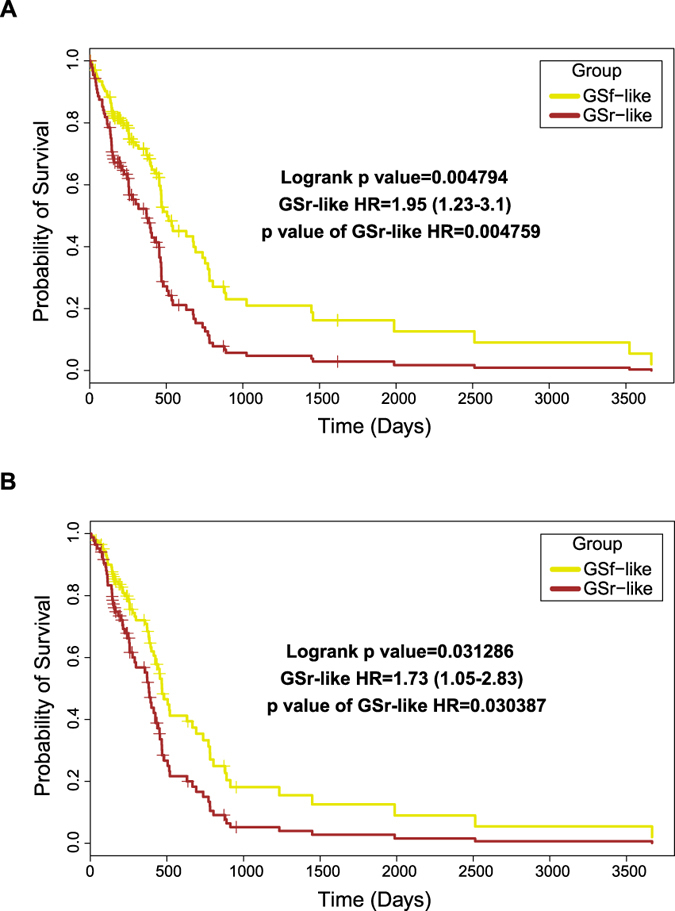
Combined levels of phospho-SRC and phospho-RPS6 predict survival of TCGA GBM patients. GSf- (yellow) or GSr-like (brown) phenotypes were assigned to patients based on the combined expression of either SRC and RPS6 pS235-36 (**A**) or SRC pY527 and RPS6 pS235-36 (**B**) see methods section for details. Patients fulfilling the SRC/RPS6 pS235-36 median expression cut-off criterion were 58 per group while for the SRC pY527/RPS6 pS235-36 were 53 per group out of a total 211 TCGA patients analyzed. Log-rank p values, hazard ratio (with 95% confidence interval) and relative p values for the group variable are reported inside each plot.

Finally, based on the subset of GSr- and GSf-like RPPA endpoints which resulted significantly different among the two groups in previous analyses, we tested all possible binary combinations of such analytes that would better stratify TCGA patients into two groups with a significant survival difference. Notably, two specific expression patterns, i.e. i) elevated total SRC or SRC pY527 coupled to low RPS6 pS235-36 and ii) high RPS6 pS235-36 coupled to low total SRC or SRC pY527, allowed us to discriminate two groups of TCGA patients: group with pattern i) displays a significantly better outcome than group with pattern ii) (Fig. 6). These results confirm the potential prognostic role of SRC and RPS6 in a large cohort of GBM patients.

## Discussion

Glioblastomas are genetically heterogeneous tumors, suggesting that a diverse set of gene products may act to regulate their behavior and ultimately their outcome. Molecular classification of GBM is still at the beginning, and there is no general consensus on GBM subtypes. Despite several studies have provided a high resolution picture of GBM molecular landscape and revealed major alterations that may drive disease pathogenesis and biology, dependencies of tumors on altered signaling pathways suitable for direct translation to the clinics have not been yet identified and exploited in this tumor. The availability of GBM stem-like cell (GSC) line models reflecting gene expression patterns and phenotypic characteristics of human GBMs^36^ more closely than serum-cultured glioma cell lines, may help identifying cues for targeted therapies^14^. Although GSC cultures may represent a valuable tool for obtaining data to be translated onto the clinical setting, their translational power is still debated for at least two limiting factors. The first one is that, using our techniques, we arose GSC cultures from approximately one third of the tumors only. This implies that the GSC paradigm might not be applied in a substantial fraction of patients suffering from GBM but mainly to more aggressive cases, since median overall and progression free survival were significantly shorter in tumors that generated GSC cultures as compared with those that did not^15^. The second issue relates to cell heterogeneity of individual GBM^11^ and within individual GSC cultures, where mixed populations of GSCs and committed progenitors coexist, both capable of forming GSCs but where only GSCs are clonogenic^37^. Nonetheless, GSC cultures represent a valuable surrogate of their parental tumor and, although bearing intrinsic limitations, they allow molecular interrogation and study of one of deadliest tumors such as GBM.

In the attempt to find druggable signaling pathways in GBM, we analyzed a collection of 17 patient-derived GSCs by applying independent but complementary molecular approaches. Taking into account the potential bias of information obtained from our limited GSC samples, we compared our observations obtained on GSCs with datasets from large public databases annotated with clinical records. We were able to define two homogeneous subtypes resembling respectively the GSf and GSr groups described by Schulte, though with distinct molecular signatures.

Comparing genes differentially expressed between the GSf-like and the GSr-like GSC groups with gene sets from the literature, highly significant overlaps were present with two of the Verhaak GBM subtype signatures (mesenchymal and proneural), two stem-cell signatures^23^,^24^, and a gene set related to oligodendrocyte differentiation^25^. The GSf-like and GSr-like clusters are characterized by a “pro-neural-like” and a “mesenchymal-like” expression signature, respectively. However, it should be emphasized that, comparing the molecular signature of our GSCs with that of GBM TCGA, which reflects the bulk tumor, may represent a limiting factor of this analysis.

Assessing more deeply the genes/transcripts that are differentially expressed between our GSf- and GSr-like lines, we found that genes located in chromosomal loci 19p13, 19p12, and 19q13 were highly enriched in the “GSC signature”. These regions contain a large number of Kruppel-type Zinc Finger transcription factors (ZNFs), which act mostly as chromatin modulating transcription repressor^20^. Several ZNFs have been identified as potential tumor suppressors and have often been found silenced in several tumor types. Deletions along chromosome 19 are common events in several malignancies including ovarian^38^ cancer, esophageal squamous cell carcinoma^39^ and oropharyngeal carcinoma^21^. The relevance of chromosome 19 alterations on the pathogenesis and prognosis of GBM deserves further evaluation.

To further investigate the signaling pathways differentially activated in GSf and GSr-like subgroups we performed RPPA analysis. We found that GSf-like lines displayed a significant increase in activation of SRC, MAPK and IGF1R/IR pathways. Conversely, GSr-like lines showed increased levels of phosphorylated proteins associated with the EGFR and PI3K/mTOR pathways.

We then evaluated the expression levels of either GSf- or GSr-like RPPA endpoints in the large cohort of GBM samples from TCGA^9^ and compared them with the proneural and mesenchymal subtypes. Although our data on GSCs do not completely match previous RPPA analysis of TCGA GBM tumors, they confirmed the relevance of mTOR pathway in defining molecular subgroups of GBM.

A complete correlation between GSf-like and proneural or GSr-like and mesenchymal subtypes was not present for all common RPPA endpoints, probably due to the different complexity of the systems (i.e. GBM tumors *versus* GSC lines) however, some of the RPPA analytes shared between TCGA and our data, displayed significant correlations with the clinical outcome of GBM patients. In particular, the GSf-like signature is characterized by the presence of active HER3, previously associated with CD133^+^ GBM stem cells^40^ and recently proposed as a potential target in GBM^32^. The GSf-like signature is also characterized by activation of proteins involved in the focal adhesion signaling axis, which is known to be driven by integrins, a hallmark of GBM stem cells^41^. The activation of HER3 and focal adhesion pathways confers a survival advantage to GBM patients in the TCGA cohort. This observation suggests that GSf-like GSCs might harbor phenotypical and molecular characteristics reminiscent of normal neural stem cells with migratory habit^42^. Conversely, the GSr-like signature is characterized by a strong activation of downstream targets of the EGFR and PI3K/mTOR pathway, which plays a critical role in cancer and, in particular, in GBM^43^. Interestingly, although the expression or activation of EGFR failed to correlate with the outcome in TCGA data, the presence of elevated levels of specific phosphorylation sites of both EGFR and RPS6, significantly associated with poor prognosis in the same cohort of GBM patients. Therefore, the GSr-like signature might be distinctive of a subset of metabolically active GSCs with high resistance to chemo- and radio-therapy hence negatively influencing the survival of GBM patients. Indeed, when classifying TCGA patients based on combined expression patterns of the two RPPA endpoints typical of either the GSf- and GSr-like phenotype (i.e. SRC and RPS6, respectively), patients with GSr-like features display a significantly shorter overall survival. Despite the small sample size of our cohort, these results are in line with the clinical data of our patients producing lines of either GSf- or GSr-like groups.

Finally, the GSf-like and GSr-like clusters were clearly confirmed by NMR metabolic profiling. The GSf-like subtype, characterized by metabolites involved in the production of neurotransmitters such as NAA and GABA, is suggestive of a prevalent neuronal metabolism. The GSr-like subtype, characterized by lack of NAA and GABA and by high mobile lipids (MLs), is indicative of a prevalent astroglial-like metabolism. These metabolic profiles corroborate the association of the GSf-like group with the pro-neural GBM subtype resulting from independent gene expression analysis and with a different prognosis. In the clinical practice, NMR spectroscopy is mainly used to differentiate GBM from lower grade gliomas^44^. Hallmarks of GBM are high choline/NAA and choline/creatinine ratios, and a lactate/lipid peak, which reflects tumor necrosis. Currently, NMR spectroscopy is not used to stratify GBM patients for prognostic purposes. In experimental settings, high glutamate signal has been correlated to IDH1 wild-type GBM^45^. In this study we showed that, at least in a subgroup of patients, the metabolic features of GBM cells, as assessed by NMR analysis, may be informative of the genomic/proteomic landscape of the tumor, and ultimately of the GBM subtype and clinical outcome. Finally, here we provide evidence that combined measurement of levels of two proteins, namely phospho-SRC and phospho-RPS6, may be informative on the outcome of GBM patients. Further investigation with increased sample size could confirm our observations providing important information on the pathogenesis of human GBM and eventually leading to an improvement of GBM diagnosis and to an indication for most appropriate therapeutic strategy.

## Methods

### Patients, diagnosis, and tumor characterization

Tumor tissue samples were collected from adult patients with GBM tumors (WHO grade IV) undergoing complete or partial surgical resection at the Institute of Neurosurgery, Catholic University School of Medicine in Rome. Informed consent was obtained from the patients before surgery. Experimental protocols were approved by the ethical committee of the Catholic University of Rome. Experiments were performed according to the guidelines approved by ethical committees of the Istituto Superiore di Sanità and Catholic University of Rome. Clinical and pathological features are summarized in Supplementary Table S3.

The expression of the proliferation marker Ki-67 and of Phosphatase and Tensin Homolog (PTEN) were characterized on tumor specimen by immunohistochemistry on deparaffinized sections using the avidin-biotin-peroxidase complex methods (ABC-Elite kit, Vector Laboratories), anti-Ki67 monoclonal antibody (MIB-1, Dako) and anti-PTEN mouse monoclonal antibody (clone 28H6; Novo Castra, Newcastle, United Kingdom). O6-methylguanine-DNA methyltransferase (MGMT) promoter methylation patterns were assessed on genomic DNA extracted from paraffin-embedded tissue by methylation-specific PCR as previously described^15^. Levels of VEGF and EGFRvIII were assessed as previously described^46^.

### Establishing GSC cultures

GSCs were isolated through mechanical dissociation of the tumor tissue and cultured in a serum-free medium supplemented with epidermal growth factor and basic fibroblast growth factor as previously described^15^. Cell lines actively proliferating required 3 to 4 weeks to be established. In these conditions, cells grow as clusters of undifferentiated cells, as indicated by morphology and expression of stem cell markers such as CD133, SOX2, Musashi-1, and nestin. The *in vivo* tumorigenic potential of GBM neurospheres was assayed by intracranial or subcutaneous cell injection in immunocompromised mice. GBM neurospheres were able to generate a tumor identical to the human tumor in antigen expression and histological tissue organization.

### Gene expression profiling

For GSC gene expression data collection, total RNA was extracted, labeled and hybridized to the Affymetrix GeneChip1.0ST array (Affymetrix, Santa Clara, CA, USA) according the manufacturer’s instructions. Data preprocessing prior to the formal statistical analysis involved standard processes of normalization [robust Multi-array Average (RMA) method]. All data analysis was performed with R (http://www.R-project.org) using Bioconductor^47^. Differentially regulated genes were determined with LIMMA^48^ applying default parameters and a FDR-corrected p value cutoff <0.05.Generation of the unified dataset involved through two consecutive steps. After downloading the data from GEO (GSE23806)^17^, or the TCGA website (https://tcga-data.nci.nih.gov/docs/publications/gbm_exp/)^8^, equivalent “best” probes from the different microarray platforms (GSCs: Affymetrix Gene Chip 1.0S^17^, GSE23806: Affymetrix HG-U133 Plus 2.0^8^, data: LBL202 dataset Affymetrix HuExon) were selected based on the Affymetrix annotation files from the NetAffx website. The combined dataset comprised 15697 probes/genes. In a second step the ComBat batch correction algorithm^49^ was used to combine the three different datasets. GSC samples and GSr/GSf samples from GSE23806 were all assigned the same SC qualifier as the first covariate variable. Likewise GBM tumor samples from TCGA and GSE23806 were all assigned a TUMOR qualifier. No explicit assignment of a GSf or GSr phenotype for either GSC or GSE23806 samples was performed during the ComBat normalization procedure. All clustering and heatmap calculation was performed using heatmap.2 of the ‘R’ package “gplots”. For the clustering of the full unified dataset including the TCGA tumor samples the top 100 genes for each tumor subset assignment were chosen from the full 840 gene signature (file TCGA_unified_CORE_ClaNC840.txt at https://tcga-data.nci.nih.gov/docs/publications/gbm_exp/). Gene set enrichment analysis was based on MSigDB using the GSEA online tool^18^,^19^ hosted by the Broad Institute (http://www.broadinstitute.org/gsea/index.jsp). Overlap of genes differentially expressed between the two GSC sample groups and the Verhaak and colleagues GBM subtype signatures was determined manually in R because a detailed examination of the lists present in MSigDB (VERHAAK_GLIOBLASTOMA_*) revealed a large discordance with the gene/GBM-subtype association present in the original file on the TCGA website (https://tcga-data.nci.nih.gov/docs/publications/gbm_exp/ TCGA_unified_CORE_ClaNC840.txt).

### 1H NMR spectroscopy

GSC 1D 1H NMR spectra were analyzed as previously described^28^. Briefly, cells were washed in PBS, centrifuged and suspended in PBS with 20% D2O and 2 mM Sodium 3-(TriMethyl-Silyl)Propionate 2,2,3,3-d (TMSP) for a frequency standard. Cell suspension were inserted in a 1 mm NMR tube and centrifuged to obtain a final packed cell sample. NMR reagents were purchased from Cambridge Isotope Laboratories, Inc. (CIL Andover MA,USA).

1D and 2D COSY 1H NMR spectra were run at 400.14 MHz on a digital Avance spectrometer equipped with a 1 mm microprobe (Bruker, AG, Darmstadt, Germany). Both 1D and 2D COSY spectra were acquired at T = 298 K. Water suppression in 1D and 2D 1H experiments was obtained using pre-saturation. The measurement of cell samples lasted approximately 210 min (60 min for the 1D experiment and 150 min for the 2D experiment).

NMR spectra were characterized by line widths in the range 9–15 Hz, which is typical of metabolite signals in intact cells and in the *in vivo* brain^50^. Chemical shifts were measured in cells with respect to Lactate methyl signal (Lac) at 1.33 ppm in 1D spectra and to Lac cross peak at 1.33–4.12 ppm in 2D COSY spectra. All NMR parameters were obtained in at least three independent experiments.

Metabolite signal assignments were performed according to indications from literature and by comparison with pure compounds.

1D peak deconvolution and integration as well as 2D COSY cross peak integration were performed by the WINNMR software (Bruker).

Individual integral values were normalized using the methyl group of cytosolic polypeptides at 0.94 ppm as internal reference for 1D spectra while for 2D spectra signal integrals were normalized to the intensity of the lysine cross-peak at 1.70–3.00 ppm.

### Reverse-phase protein microarrays

RPPAs were performed as previously described^35^. Briefly, cell lysates were diluted with 2X Tris-Glycine SDS Sample Buffer (Life Technologies Corporation, Carlsbad, CA) prior to printing on nitrocellulose slides (Grace Bio-Labs, Bend, OR, USA) and were spotted in triplicate with the Aushon 2470 contact pin arrayer (Aushon Bio Systems Inc., Billerica, MA), in 4-point two-fold dilution curves. Positive and negative expression control lysates were printed on every slide in a ten-point two-fold dilution curve. After incubation for 2 hours with I-Block (Life Technologies), array staining with antibodies was carried out on an automated slide stainer per manufacturer’s instructions (Autostainer CSA kit, DAKO, Carpinteria, CA). Biotinylated secondary antibody was either goat anti-rabbit IgG H + L (Vector Labs, Burlingame, CA) or anti-mouse (from CSA kit, DAKO). Streptavidin-conjugated IRDye680LT^®^ (LI-COR Biosciences, Lincoln, NE, USA) was used as a final signal generating step. All antibodies used in these experiments are listed in Supplementary Table S2. Total protein concentration values were assessed by staining with Sypro Ruby Blot Stain (Life Technologies). Stained slides were scanned on a Tecan Power Scanner (Männedorf, Switzerland) equipped with a customized emission filter to increase efficiency in collection of IRDye680LT^®^ fluorescence. Image analysis for spot recognition, quantification and normalization was carried out using MicroVigene 5.1 software (Vigene Tech Inc., Carlisle, MA, USA).

### Statistical analysis

For ^1^H NMR cluster analysis, agglomerative hierarchical clustering was performed utilizing XLSTAT software, Addinsoft TM, version 2012.2.02. Values of log2 fold change (FC) resulting from the comparison between the two main clusters were calculated for each metabolite. Student’s t test was performed utilizing XLSTAT software, Addinsoft TM, version 2012.2.02.

RPPA data analysis was performed on standardized data using dedicated packages of the ‘R’ software (http://www.r-project.org/, packages: Bioconductor, Heatplus, ggplot2, plyr, gplots, coin). Briefly, after local background correction, signal for secondary antibody staining alone was subtracted to raw antibody intensities for all spots and further normalization over the corresponding total protein staining allowed correction of potential loading biases. Standardized data were generated individually for each antibodies by subtracting the sample mean and dividing by the standard deviation (z scores). Wilcoxon rank sum test was used to compare RPPA data generated on our GSC lines while unpaired t test was used for statistical analysis of expression levels between P and M subtypes in TCGA level 3 RPPA data downloaded from http://gbm-biodp.nci.nih.gov. Statistical significance was accepted for p values lower than 0.05.

### TCGA database bioinformatic analyses

In order to investigate GBM RNAseq and RPPA data from the TCGA project, we exploited ‘Next-Generation Clustered Heatmaps’ available at the Pan-Cancer NG-CHM Compendium website (http://bioinformatics.mdanderson.org/TCGA/NGCHMPortal/), hosted by the bioinformatics department of MD Anderson, University of Texas (Houston, TX, USA). Most of our results for the mRNA expression refer to the 3-Platform Aggregates used by Verhaak and colleagues^8^. To study the relationships between RPPA endpoints associated with GSf- and GSr-like clusters and patient outcome in TCGA data we used the GBM-BioDP software platform^51^ (http://gbm-biodp.nci.nih.gov). Briefly, based on (phospho-)proteins that resulted significantly modulated between GSr- and GSf-like GSC clusters in our RPPA analysis, we generated two lists of corresponding gene symbols (Supplementary Table S6) and we queried the GBM-BioDP web platform. For further analysis we selected only those RPPA endpoints shared between our analyses on GSCs and that performed in the TCGA, either as total, phosphorylated or both. Thus, we evaluated whether the levels in P and M samples were in line or not with the RPPA levels measured in GSf-like versus GSr-like in our GSC samples (Supplementary Table S6). Methods used to compare RPPA levels between the P and M subtypes are described in the previous paragraph. The prognostic index for each patients was calculated as the sum of the products of the linear components of the Cox model and the corresponding RPPA values of the endpoints included as covariates in the full model (Fig. 5B,D). Survival analysis in Fig. 6 was performed on TCGA data downloaded directly via ‘TCGA Data Matrix’ (http://tcga-data.nci.nih.gov/tcga/dataAccessMatrix.htm) and using as event variable either ‘days to death’ for deceased patients or the maximum follow-up time for all other patients. Briefly, stratification of TCGA patients in either the GSf- or the GSr-like group was done by applying RPPA expression cut-offs for selected endpoint combinations. To this end we decided to use a subset of RPPA analytes differentially expressed between P and M subtypes or statistically significant covariates (p < 0.05) in single or combined Cox model done either on the full cohort of patients or on P and M subtypes, i.e. EGFR pY1173, RPS6 pS235-36, HER3 pY1289, SRC and SRC pY527. Based on this subset of RPPA endpoints, we tested all possible binary combinations of GSf- and GSr-like analytes using median expression level cut-offs, e.g. patients having expression values above the median for either SRC or SRC pY527 or HER3 pY1289 and simultaneous lower than median levels of either RPS6 pS235-36 or EGFR pY1173 were assigned the GSF-like group while patients with opposite pattern were considered as GSr-like. Survival and prognostic index analyses have been performed by means of the *survival* package of the ‘R’ software. The results shown in the present manuscript are in part based upon data generated by the TCGA Research Network (http://cancergenome.nih.gov/).

## Additional Information

**How to cite this article**: Marziali, G. *et al*. Metabolic/Proteomic Signature Defines Two Glioblastoma Subtypes With Different Clinical Outcome. *Sci. Rep.* **6**, 21557; doi: 10.1038/srep21557 (2016).

## Supplementary Material

Supplementary Information

## References

[b1] Dunn G. P. . Emerging insights into the molecular and cellular basis of glioblastoma. Genes Dev 26, 756–784 (2012). 22508724 10.1101/gad.187922.112 PMC3337451

[b2] Stupp R. . Radiotherapy plus concomitant and adjuvant temozolomide for glioblastoma. N Engl J Med 352, 987–996 (2005). 15758009 10.1056/NEJMoa043330

[b3] Chabner B. A. Early accelerated approval for highly targeted cancer drugs. N Engl J Med 364, 1087–1089 (2011). 21428763 10.1056/NEJMp1100548

[b4] Phillips H. S. . Molecular subclasses of high-grade glioma predict prognosis, delineate a pattern of disease progression, and resemble stages in neurogenesis. Cancer Cell 9, 157–173 (2006). 16530701 10.1016/j.ccr.2006.02.019

[b5] Comprehensive genomic characterization defines human glioblastoma genes and core pathways. *Nature* **455**, 1061–1068 (2008). 10.1038/nature07385 PMC2671642 18772890

[b6] Parsons D. W. . An integrated genomic analysis of human glioblastoma multiforme. Science 321, 1807–1812 (2008). 18772396 10.1126/science.1164382 PMC2820389

[b7] Noushmehr H. . Identification of a CpG island methylator phenotype that defines a distinct subgroup of glioma. Cancer Cell 17, 510–522 (2010). 20399149 10.1016/j.ccr.2010.03.017 PMC2872684

[b8] Verhaak R. G. . Integrated genomic analysis identifies clinically relevant subtypes of glioblastoma characterized by abnormalities in PDGFRA, IDH1, EGFR, and NF1. Cancer Cell 17, 98–110 (2010). 20129251 10.1016/j.ccr.2009.12.020 PMC2818769

[b9] Brennan C. W. . The somatic genomic landscape of glioblastoma. Cell 155, 462–477 (2013). 24120142 10.1016/j.cell.2013.09.034 PMC3910500

[b10] Thiery J. P. Epithelial-mesenchymal transitions in tumour progression. Nat Rev Cancer 2, 442–454 (2002). 12189386 10.1038/nrc822

[b11] Patel A. P. . Single-cell RNA-seq highlights intratumoral heterogeneity in primary glioblastoma. Science 344, 1396–1401 (2014). 24925914 10.1126/science.1254257 PMC4123637

[b12] Singh S. K. . Identification of human brain tumour initiating cells. Nature 432, 396–401 (2004). 15549107 10.1038/nature03128

[b13] Ernst A. . Genomic and expression profiling of glioblastoma stem cell-like spheroid cultures identifies novel tumor-relevant genes associated with survival. Clin Cancer Res 15, 6541–6550 (2009). 19861460 10.1158/1078-0432.CCR-09-0695

[b14] Lee J. . Tumor stem cells derived from glioblastomas cultured in bFGF and EGF more closely mirror the phenotype and genotype of primary tumors than do serum-cultured cell lines. Cancer Cell 9, 391–403 (2006). 16697959 10.1016/j.ccr.2006.03.030

[b15] Pallini R. . Cancer stem cell analysis and clinical outcome in patients with glioblastoma multiforme. Clin Cancer Res 14, 8205–8212 (2008). 19088037 10.1158/1078-0432.CCR-08-0644

[b16] Gunther H. S. . Glioblastoma-derived stem cell-enriched cultures form distinct subgroups according to molecular and phenotypic criteria. Oncogene 27, 2897–2909 (2008). 18037961 10.1038/sj.onc.1210949

[b17] Schulte A. . A distinct subset of glioma cell lines with stem cell-like properties reflects the transcriptional phenotype of glioblastomas and overexpresses CXCR4 as therapeutic target. Glia 59, 590–602 (2011). 21294158 10.1002/glia.21127

[b18] Mootha V. K. . PGC-1alpha-responsive genes involved in oxidative phosphorylation are coordinately downregulated in human diabetes. Nat Genet 34, 267–273 (2003). 12808457 10.1038/ng1180

[b19] Subramanian A. . Gene set enrichment analysis: a knowledge-based approach for interpreting genome-wide expression profiles. Proc Natl Acad Sci USA 102, 15545–15550 (2005). 16199517 10.1073/pnas.0506580102 PMC1239896

[b20] Lukic S., Nicolas J. C. & Levine A. J. The diversity of zinc-finger genes on human chromosome 19 provides an evolutionary mechanism for defense against inherited endogenous retroviruses. Cell Death Differ 21, 381–387 (2014). 24162661 10.1038/cdd.2013.150 PMC3921586

[b21] Lleras R. A. . Hypermethylation of a cluster of Kruppel-type zinc finger protein genes on chromosome 19q13 in oropharyngeal squamous cell carcinoma. Am J Pathol 178, 1965–1974 (2011). 21514414 10.1016/j.ajpath.2011.01.049 PMC3081156

[b22] Roversi G. . Identification of novel genomic markers related to progression to glioblastoma through genomic profiling of 25 primary glioma cell lines. Oncogene 25, 1571–1583 (2006). 16247447 10.1038/sj.onc.1209177

[b23] Wong D. J. . Module map of stem cell genes guides creation of epithelial cancer stem cells. Cell Stem Cell 2, 333–344 (2008). 18397753 10.1016/j.stem.2008.02.009 PMC2628721

[b24] Boquest A. C. . Isolation and transcription profiling of purified uncultured human stromal stem cells: alteration of gene expression after *in vitro* cell culture. Molecular biology of the cell 16, 1131–1141 (2005). 15635089 10.1091/mbc.E04-10-0949 PMC551479

[b25] Gobert R. P. . Convergent functional genomics of oligodendrocyte differentiation identifies multiple autoinhibitory signaling circuits. Mol Cell Biol 29, 1538–1553 (2009). 19139271 10.1128/MCB.01375-08 PMC2648232

[b26] DeAngelis L. M. & Mellinghoff I. K. Virchow 2011 or how to ID(H) human glioblastoma. Journal of clinical oncology : official journal of the American Society of Clinical Oncology 29, 4473–4474 (2011). 22025161 10.1200/JCO.2011.37.5873

[b27] Lai A. . Evidence for sequenced molecular evolution of IDH1 mutant glioblastoma from a distinct cell of origin. Journal of clinical oncology : official journal of the American Society of Clinical Oncology 29, 4482–4490 (2011). 22025148 10.1200/JCO.2010.33.8715 PMC3236649

[b28] Guidoni L. . 1H NMR detects different metabolic profiles in glioblastoma stem-like cells. NMR Biomed 27, 129–145 (2014). 24142746 10.1002/nbm.3044

[b29] Opstad K. S., Bell B. A., Griffiths J. R. & Howe F. A. An investigation of human brain tumour lipids by high-resolution magic angle spinning 1H MRS and histological analysis. NMR Biomed 21, 677–685 (2008). 18186027 10.1002/nbm.1239

[b30] Castillo M., Smith J. K. & Kwock L. Correlation of myo-inositol levels and grading of cerebral astrocytomas. AJNR Am J Neuroradiol 21, 1645–1649 (2000). 11039343 PMC8174883

[b31] Sato A. . Pivotal role for ROS activation of p38 MAPK in the control of differentiation and tumor-initiating capacity of glioma-initiating cells. Stem Cell Res 12, 119–131 (2014). 24185179 10.1016/j.scr.2013.09.012

[b32] Akbani R. . A pan-cancer proteomic perspective on The Cancer Genome Atlas. Nat Commun 5, 3887 (2014). 24871328 10.1038/ncomms4887 PMC4109726

[b33] Zhang B. . Proteogenomic characterization of human colon and rectal cancer. Nature 513, 382–387 (2014). 25043054 10.1038/nature13438 PMC4249766

[b34] Cancer Genome Atlas Research, N. Comprehensive molecular profiling of lung adenocarcinoma. Nature 511, 543–550 (2014). 25079552 10.1038/nature13385 PMC4231481

[b35] Signore M. . Combined PDK1 and CHK1 inhibition is required to kill glioblastoma stem-like cells *in vitro* and *in vivo*. Cell Death Dis 5, e1223 (2014). 24810059 10.1038/cddis.2014.188 PMC4047898

[b36] Piccirillo S. G. . Genetic and functional diversity of propagating cells in glioblastoma. Stem Cell Reports 4, 7–15 (2015). 25533637 10.1016/j.stemcr.2014.11.003 PMC4297869

[b37] Chen J. . A restricted cell population propagates glioblastoma growth after chemotherapy. Nature 488, 522–526 (2012). 22854781 10.1038/nature11287 PMC3427400

[b38] Gorringe K. L. . Are there any more ovarian tumor suppressor genes? A new perspective using ultra high-resolution copy number and loss of heterozygosity analysis. Genes Chromosomes Cancer 48, 931–942 (2009). 19603523 10.1002/gcc.20694

[b39] Zhu Y. H. . Downregulation of the novel tumor suppressor DIRAS1 predicts poor prognosis in esophageal squamous cell carcinoma. Cancer Res 73, 2298–2309 (2013). 23436800 10.1158/0008-5472.CAN-12-2663

[b40] Duhem-Tonnelle V. . Differential distribution of erbB receptors in human glioblastoma multiforme: expression of erbB3 in CD133-positive putative cancer stem cells. J Neuropathol Exp Neurol 69, 606–622 (2010). 20467331 10.1097/NEN.0b013e3181e00579 PMC3173752

[b41] Lathia J. D. . Integrin alpha 6 regulates glioblastoma stem cells. Cell Stem Cell 6, 421–432 (2010). 20452317 10.1016/j.stem.2010.02.018 PMC2884275

[b42] Vescovi A. L., Galli R. & Reynolds B. A. Brain tumour stem cells. Nat Rev Cancer 6, 425–436 (2006). 16723989 10.1038/nrc1889

[b43] Liu P., Cheng H., Roberts T. M. & Zhao J. J. Targeting the phosphoinositide 3-kinase pathway in cancer. Nat Rev Drug Discov 8, 627–644 (2009). 19644473 10.1038/nrd2926 PMC3142564

[b44] Stadlbauer A. . Preoperative grading of gliomas by using metabolite quantification with high-spatial-resolution proton MR spectroscopic imaging. Radiology 238, 958–969 (2006). 16424238 10.1148/radiol.2382041896

[b45] Chaumeil M. M. . Hyperpolarized [1-13C] glutamate: a metabolic imaging biomarker of IDH1 mutational status in glioma. Cancer Res 74, 4247–4257 (2014). 24876103 10.1158/0008-5472.CAN-14-0680 PMC4134724

[b46] Martini M. . Epigenetic silencing of Id4 identifies a glioblastoma subgroup with a better prognosis as a consequence of an inhibition of angiogenesis. Cancer 119, 1004–1012 (2013). 23132729 10.1002/cncr.27821

[b47] Gentleman R. C. . Bioconductor: open software development for computational biology and bioinformatics. Genome biology 5, R80 (2004). 15461798 10.1186/gb-2004-5-10-r80 PMC545600

[b48] Ritchie M. E. . limma powers differential expression analyses for RNA-sequencing and microarray studies. Nucleic acids research 43, e47 (2015). 25605792 10.1093/nar/gkv007 PMC4402510

[b49] Johnson W. E., Li C. & Rabinovic A. Adjusting batch effects in microarray expression data using empirical Bayes methods. Biostatistics 8, 118–127 (2007). 16632515 10.1093/biostatistics/kxj037

[b50] Mlynarik V., Cudalbu C., Xin L. & Gruetter R. 1H NMR spectroscopy of rat brain *in vivo* at 14.1Tesla: improvements in quantification of the neurochemical profile. J Magn Reson 194, 163–168 (2008). 18703364 10.1016/j.jmr.2008.06.019

[b51] Celiku O., Johnson S., Zhao S., Camphausen K. & Shankavaram U. Visualizing molecular profiles of glioblastoma with GBM-BioDP. PLoS One 9, e101239 (2014). 25010047 10.1371/journal.pone.0101239 PMC4091869

