# Supplementary material for: Metabolic/Proteomic Signature Defines Two Glioblastoma Subtypes With Different Clinical Outcome
Source: Sci Rep. 2016 Feb 9;6:21557. doi: 10.1038/srep21557 (PMC4746700; doi:10.1038/srep21557)
Supplement: Supplementary Information [file srep21557-s1.pdf]

METABOLIC/PROTEOMIC SIGNATURE DEFINES TWO GLIOBLASTOMA SUBTYPES WITH DIFFERENT CLINICAL OUTCOME

G. Marziali[1]*, M. Signore[1]*, M. Buccarelli[1], S. Grande[2], A. Palma[2], M. Biffoni[1], A. Rosi[2], Q. G. D'Alessandris[3], M. Martini[4], L. M. Larocca[4], R. De Maria[5], R. Pallini[3§] and L. Ricci-Vitiani[1§]

[1] Department of Hematology, Oncology and Molecular Medicine, Istituto Superiore di Sanità, Rome, Italy;

[2] Department of Technology and Health, Istituto Superiore di Sanità, Rome, Italy;

[3] Institute of Neurosurgery, Università Cattolica del Sacro Cuore, Rome, Italy;

[4] Institute of Anatomic Pathology, Università Cattolica del Sacro Cuore, Rome, Italy;

[5] Regina Elena National Cancer Institute, Rome, Italy;

*These authors equally contributed to the manuscript

[§]These authors shared senior authorship

Correspondence: Lucia Ricci-Vitiani (E-mail: lriccivitiani@yahoo.it) and Giovanna Marziali (E-mail: giovanna.marziali@iss.it), Department of Hematology, Oncology and Molecular Medicine, Istituto Superiore di Sanità, Viale Regina Elena 299, 00161 Rome, Italy

This file includes:

Supplementary Figures S1-7

Supplementary Tables S1-8

Supplementary Figure S1a

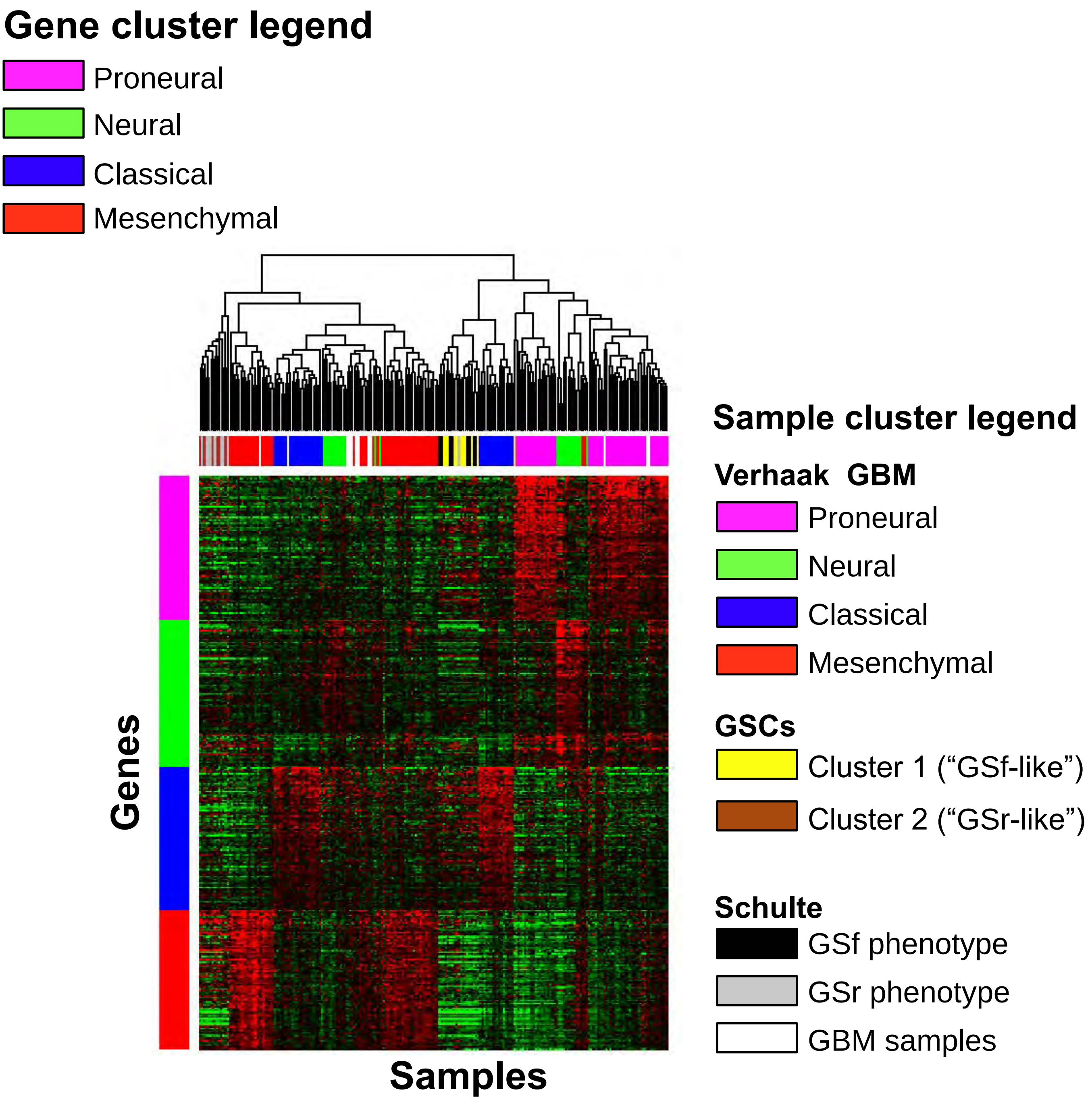

Supplementary Figure S1b

Top 1000 most variable genes:
GSCs cluster 1 vs cluster 2

Top 1000 most variable genes:
combined GSCs and
Schulte GSr/GSf samples.

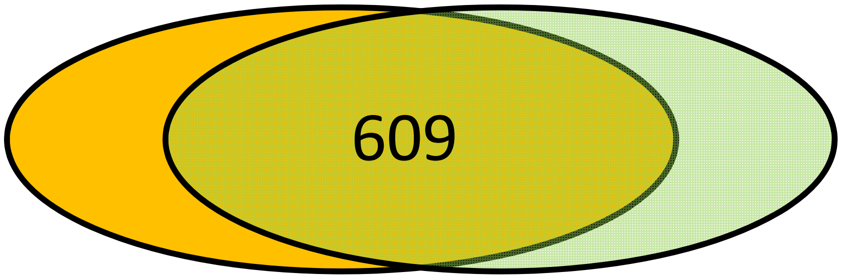

GSEA GO enrichment present only in
"GSC cluster 1 vs cluster 2"

GO Gene Set	# Genes in Overlap (k)	FDR q-value
CELL_CYCLE_GO_0007049	32	2.07E-11
MITOTIC_CELL_CYCLE	22	8.02E-11
NUCLEOBASENUCLEOSIDENUCLEOTIDE_AND_NUCLEIC_ACID_METABOLIC_PROCESS	68	1.11E-10
POSITIVE_REGULATION_OF_CELL_PROLIFERATION	21	3.27E-10
NEUROGENESIS	17	4.20E-10
CELL_CYCLE_PHASE	22	5.51E-10
PROTEIN_KINASE_ACTIVITY	28	8.47E-10
TRANSCRIPTION	48	8.86E-10
CELL_CYCLE_PROCESS	23	9.74E-10
INTRACELLULAR_SIGNALING_CASCADE	44	1.88E-09
CYTOSKELETON	31	3.27E-09
KINASE_ACTIVITY	31	3.68E-09
REGULATION_OF_NUCLEOBASENUCLEOSIDENUCLEOTIDE_AND_NUCLEIC_ACID_METABOLIC_PROCESS	41	6.69E-09
NUCLEUS	69	1.17E-08
NEURON_DIFFERENTIATION	14	1.76E-08
REGULATION_OF_TRANSCRIPTION	38	1.96E-08
REGULATION_OF_GENE_EXPRESSION	42	2.30E-08
RESPONSE_TO_CHEMICAL_STIMULUS	27	2.88E-08
INTRACELLULAR_NON_MEMBRANE_BOUND_ORGANELLE	40	3.47E-08
NON_MEMBRANE_BOUND_ORGANELLE	40	3.47E-08
RECEPTOR_BINDING	29	8.72E-08
NEURON_DEVELOPMENT	12	1.11E-07
PROTEIN_KINASE_CASCADE	25	1.22E-07
TRANSMEMBRANE_RECEPTOR_PROTEIN_KINASE_ACTIVITY	11	1.62E-07
COLLAGEN	8	2.75E-07
POST_TRANSLATIONAL_PROTEIN_MODIFICATION	32	3.16E-07
AXONOGENESIS	10	3.33E-07
NITROGEN_COMPOUND_METABOLIC_PROCESS	17	7.22E-07
DNA_BINDING	36	7.58E-07
TRANSCRIPTION_FACTOR_BINDING	24	1.02E-06

GSEA GO enrichment present only in
"combined GSCs and Schulte GSr/GSf samples"

GO Gene Set	# Genes in Overlap (k)	FDR q-value
PHOSPHORIC_ESTER_HYDROLASE_ACTIVITY	19	9.07E-09
SUBSTRATE_SPECIFIC_TRANSMEMBRANE_TRANSPORTER_ACTIVITY	28	1.45E-08
SUBSTRATE_SPECIFIC_TRANSPORTER_ACTIVITY	30	1.47E-08
TRANSMEMBRANE_TRANSPORTER_ACTIVITY	29	2.15E-08
MEMBRANE_FRACTION	27	4.20E-08
INTRACELLULAR_SIGNALING_CASCADE	39	8.94E-08
RESPONSE_TO_WOUNDING	19	2.75E-07
IONOTROPIC_GLUTAMATE_RECEPTOR_ACTIVITY	6	3.14E-07
REGULATION_OF_SIGNAL_TRANSDUCTION	20	6.35E-07
REGULATION_OF_PHOSPHORYLATION	10	9.17E-07
ION_BINDING	22	9.24E-07
HOMEOSTATIC_PROCESS	19	9.79E-07
CELL_SURFACE	12	1.25E-06
LIPID_RAFT	8	1.61E-06
TRANSPORT	40	2.59E-06
EXTRACELLULAR_STRUCTURE_ORGANIZATION_AND_BIOGENESIS	8	3.42E-06
ENZYME_ACTIVATOR_ACTIVITY	14	4.42E-06
POSITIVE_REGULATION_OF_CATALYTIC_ACTIVITY	16	4.47E-06
SYNAPSE_ORGANIZATION_AND_BIOGENESIS	7	4.47E-06
BIOSYNTHETIC_PROCESS	28	6.76E-06
ESTABLISHMENT_OF_LOCALIZATION	41	8.27E-06
CELLULAR_MORPHOGENESIS_DURING_DIFFERENTIATION	9	8.39E-06
CHEMICAL_HOMEOSTASIS	15	9.46E-06
GLUTAMATE_SIGNALING_PATHWAY	6	1.06E-05
REGULATION_OF_TRANSFERASE_ACTIVITY	15	1.42E-05
ION_TRANSMEMBRANE_TRANSPORTER_ACTIVITY	20	1.49E-05
POSITIVE_REGULATION_OF_TRANSFERASE_ACTIVITY	11	1.75E-05
GTPASE_REGULATOR_ACTIVITY	13	2.05E-05
CELLULAR_HOMEOSTASIS	14	2.27E-05
METAL_ION_TRANSMEMBRANE_TRANSPORTER_ACTIVITY	14	2.27E-05

Supplementary Figure S2

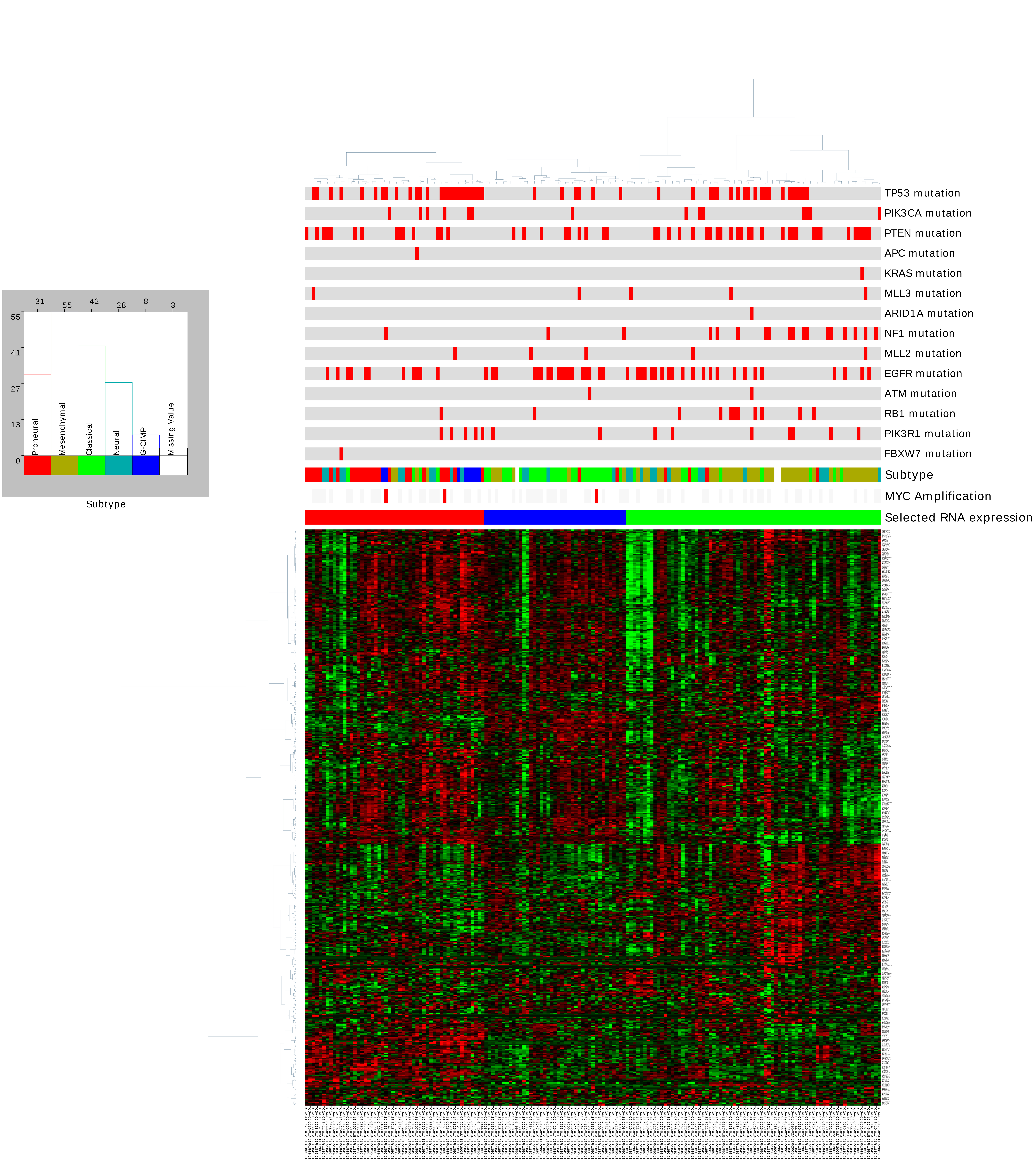

Supplementary Figure S3

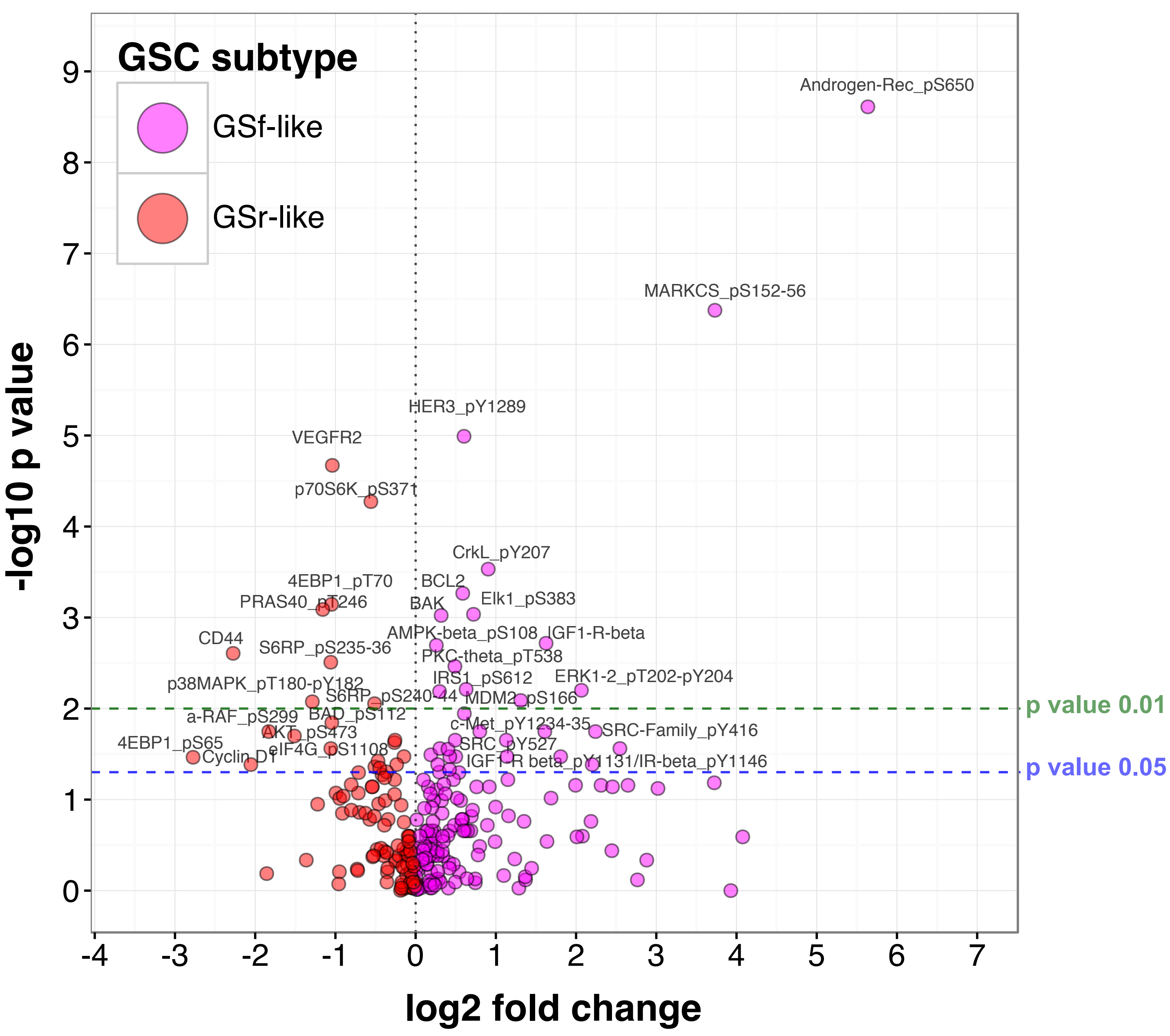

Supplementary Figure S4

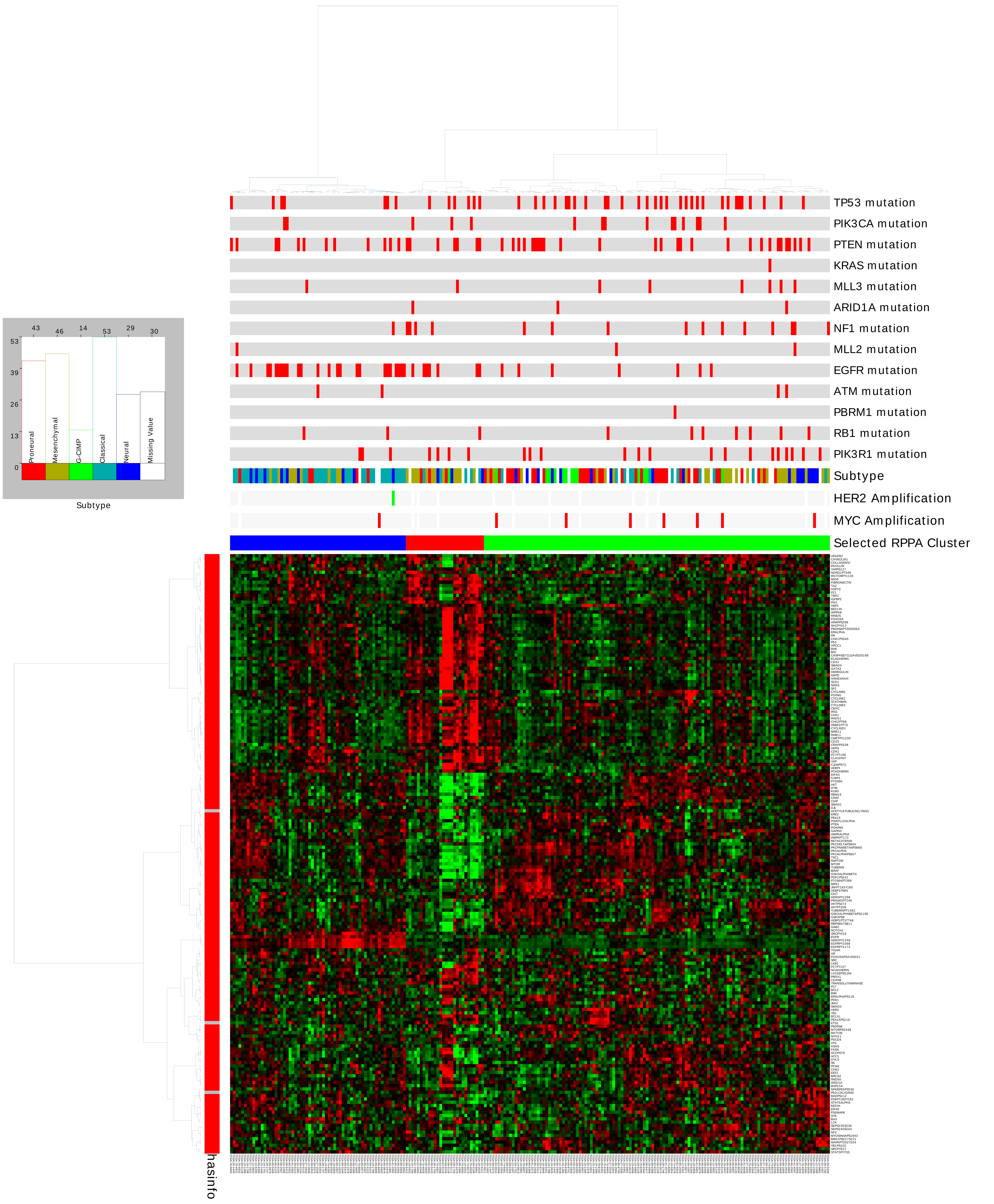

Supplementary Figure S5

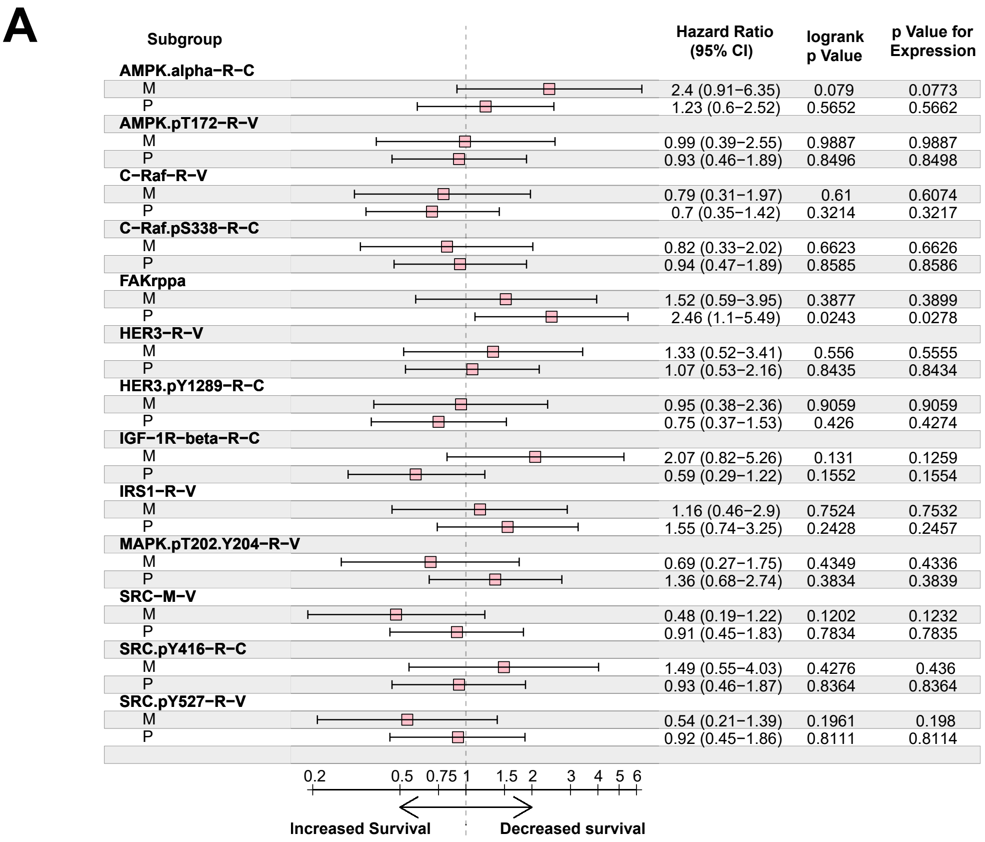

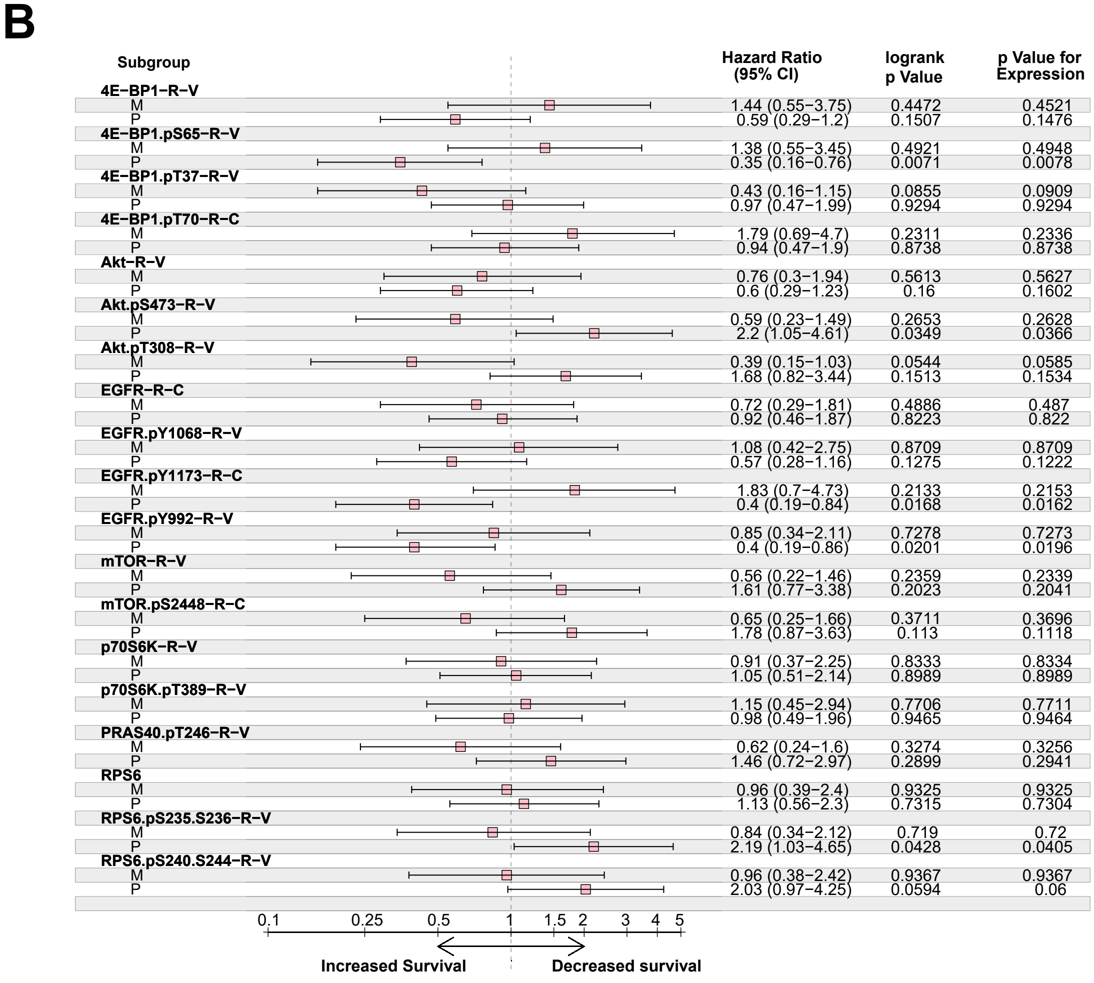

Supplementary Figure S6

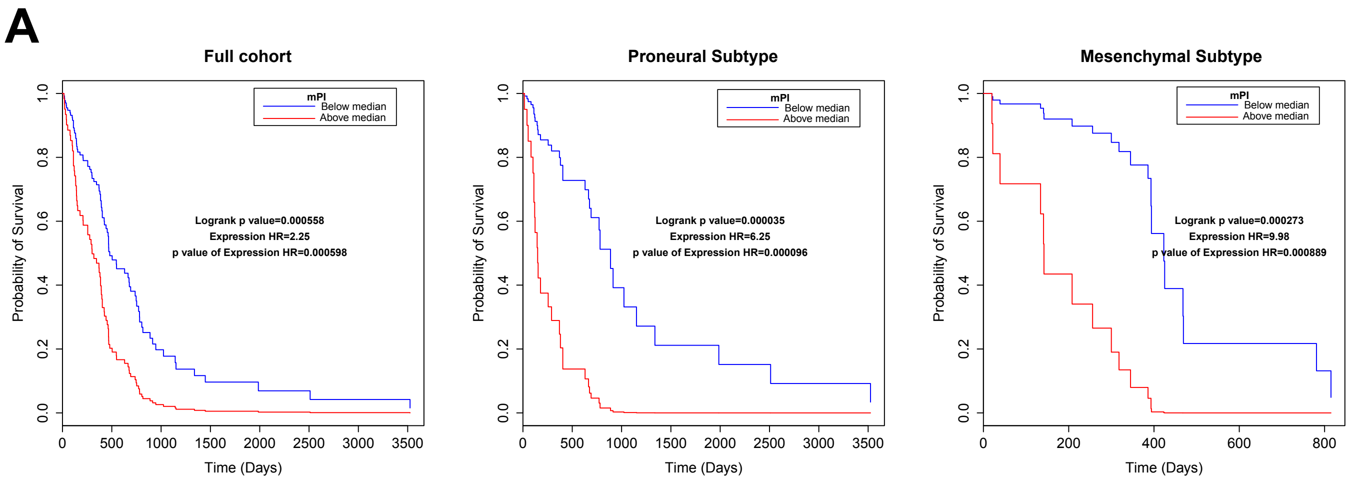

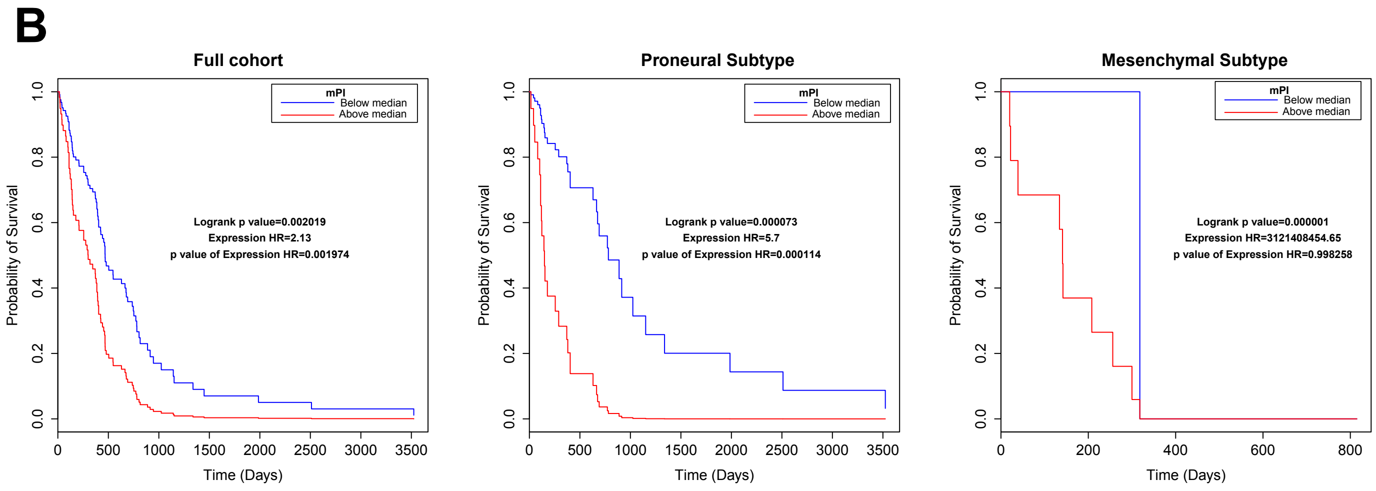

Supplementary Figure S7

A

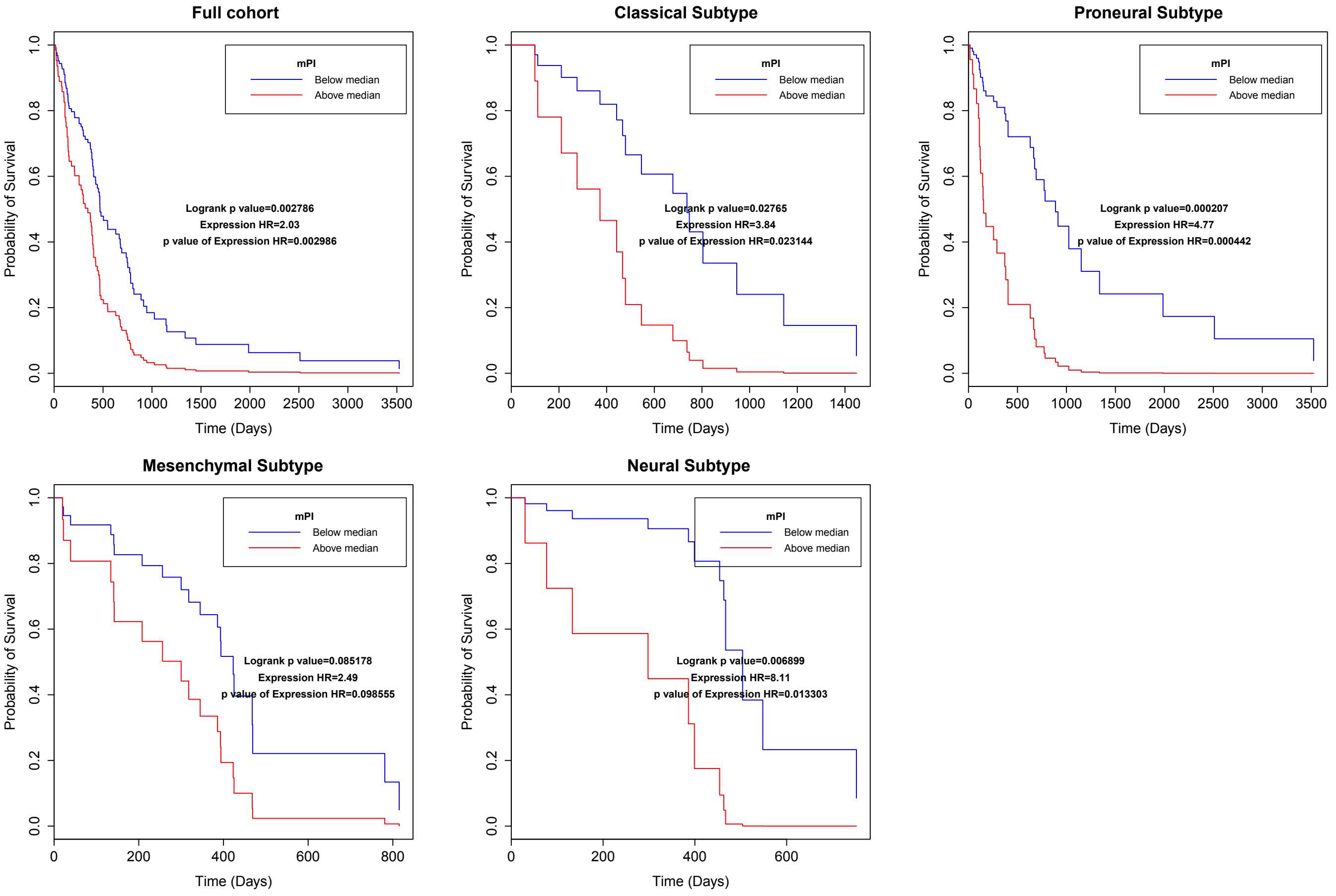

B

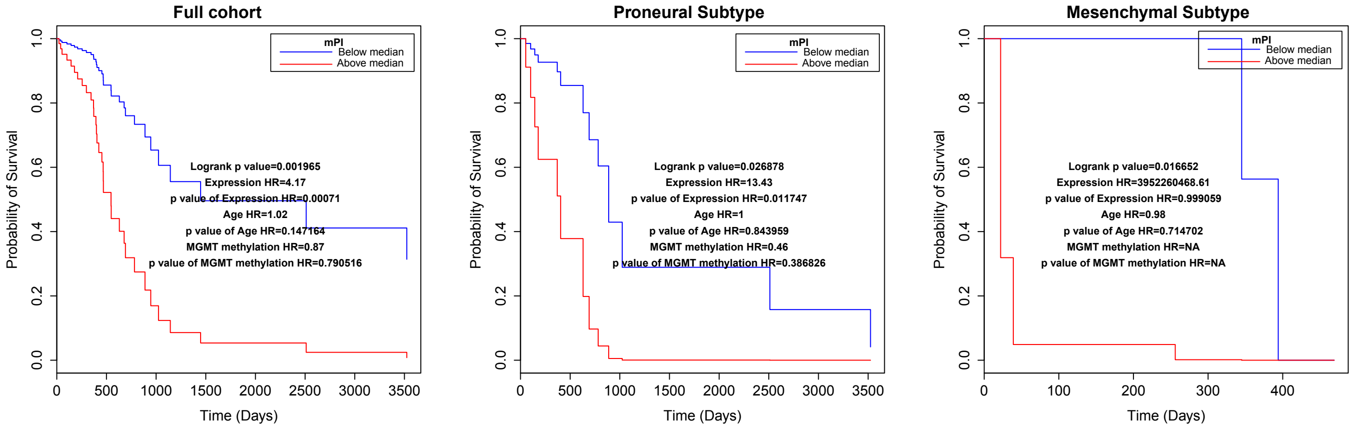

Supplementary Table S1a. Differentially expressed gene cluster1-GSf *vs* cluster2-GSr

Probe	Gene	logFC GSf vs GSr	FC GSf vs GSr	Ave Expr	t	p value	adj. p value	B	chr19p13	chr19p12	chr19q13	VER_PN	VER_CL	VER_MES	BOQUEST_STEM	WONG_STEM	GOBERT
7921003	C1ORF61	5,06	33,39	7,60	5,13	8,28E-05	2,24E-02	1,67				■					
7953200	CCND2	4,98	31,60	9,87	5,96	1,53E-05	1,60E-02	3,21									
8095585	SLC4A4	3,69	12,95	8,64	4,37	0,0004152	3,84E-02	0,19					■				
8105585	RNF180	3,49	11,22	6,96	4,07	0,0007928	4,89E-02	-0,40									
8123920	ELOVL2	3,49	11,22	7,26	5,96	1,53E-05	1,60E-02	3,21					■		■		
8081620	TAGLN3	3,47	11,05	7,27	6,79	3,12E-06	1,10E-02	4,63								■	
8168622	KLHL4	3,35	10,22	7,09	5,14	8,18E-05	2,24E-02	1,69					■				
8168589	ZNF711	3,31	9,91	7,26	5,35	5,29E-05	2,20E-02	2,08				■					
7922018	ILDR2	3,21	9,25	7,23	5,40	4,79E-05	2,20E-02	2,17									
7961829	BCAT1	3,05	8,27	9,16	4,55	0,0002854	3,35E-02	0,54									
8121712	SLC35F1	3,04	8,23	8,18	4,20	0,0006037	4,46E-02	-0,15									
8064790	RASSF2	2,93	7,63	7,95	5,46	4,23E-05	2,20E-02	2,29									■
8110990	CTNND2	2,89	7,39	7,85	6,97	2,23E-06	1,02E-02	4,93									
7958305	RFX4	2,88	7,36	6,14	5,32	5,61E-05	2,20E-02	2,03									
8090690	CPNE4	2,67	6,37	7,08	5,00	0,0001087	2,34E-02	1,42									
7918449	KCNA2	2,63	6,18	6,75	4,12	0,000714	4,75E-02	-0,31								■	
7905131	CA14	2,61	6,11	6,87	4,37	0,0004152	3,84E-02	0,19									■
8106827	GPR98	2,54	5,82	6,41	4,26	0,0005262	4,26E-02	-0,03									
8004545	ATP1B2	2,50	5,67	8,75	4,72	0,0001969	2,96E-02	0,88									■
8092520	C3ORF70	2,48	5,57	7,24	4,12	0,0007136	4,75E-02	-0,31									
8150419	ZMAT4	2,37	5,19	6,03	4,25	0,0005376	4,29E-02	-0,05									
8001197	NETO2	2,36	5,12	9,40	4,44	0,0003575	3,62E-02	0,33									
8034263	ELAVL3	2,35	5,09	6,99	4,59	0,0002597	3,25E-02	0,62	■								■
8083360	MED12L	2,34	5,05	7,70	4,57	0,0002739	3,32E-02	0,58									
8042356	MEIS1	2,33	5,02	8,55	4,45	0,00035	3,62E-02	0,35					■		■	■	
8072088	SEZ6L	2,29	4,90	7,17	4,72	0,0001967	2,96E-02	0,88				■					
8004152	WSCD1	2,28	4,85	7,15	5,11	8,66E-05	2,28E-02	1,63					■				■
8160024	GLDC	2,24	4,74	9,38	4,18	0,000632	4,50E-02	-0,20									
8116835	GCNT2	2,24	4,74	5,88	5,02	0,0001054	2,34E-02	1,45									
8046078	B3GALT1	2,23	4,70	6,94	4,69	0,000212	3,08E-02	0,81					■				
7951977	FXYD6	2,20	4,60	8,64	4,79	0,0001687	2,79E-02	1,02				■					
7910727	ACTN2	2,19	4,55	6,34	5,09	9,11E-05	2,28E-02	1,59									
7993126	ABAT	2,18	4,52	8,13	5,38	5,01E-05	2,20E-02	2,13				■					
8068231	OLIG2	2,17	4,49	6,96	4,22	0,0005799	4,40E-02	-0,12				■					
8058542	C2ORF80	2,16	4,48	5,36	4,46	0,0003396	3,59E-02	0,38									
7986068	BLM	2,16	4,48	7,67	5,67	2,76E-05	2,06E-02	2,68					■				■
8143564	FAM131B	2,15	4,44	7,69	4,36	0,0004287	3,88E-02	0,16									
8160521	MOB3B	2,13	4,39	6,96	5,23	6,78E-05	2,24E-02	1,86									
8112841	HOMER1	2,10	4,29	8,62	5,00	0,0001085	2,34E-02	1,43									■
8089729	IGSF11	2,07	4,19	7,03	5,20	7,19E-05	2,24E-02	1,80									■
8146579	CHD7	2,06	4,18	8,65	4,16	0,000659	4,59E-02	-0,23				■					
7946365	STK33	2,05	4,16	7,44	4,61	0,0002464	3,18E-02	0,67									
8145611	FZD3	2,05	4,14	9,30	4,74	0,0001873	2,92E-02	0,92					■				
7982187	APBA2	2,02	4,06	7,77	5,34	5,43E-05	2,20E-02	2,06									■
8173647	ZDHHC15	2,02	4,04	6,59	5,41	4,70E-05	2,20E-02	2,19									
7962312	ABCD2	2,01	4,02	5,98	4,29	0,0004946	4,14E-02	0,03					■				

Probe	Gene	logFC GSf vs GSr	FC GSf vs GSr	Ave Expr	t	p value	adj. p value	B	chr19p13	chr19p12	chr19q13	VER_PN	VER_CL	VER_MES	BOQUEST_STEM	WONG_STEM	GOBERT
7989647	KIAA0101	1,97	3,93	8,76	4,61	0,0002478	3,18E-02	0,67									
8040725	DPYSL5	1,97	3,93	7,65	5,26	6,35E-05	2,24E-02	1,92									
7925250	GNG4	1,97	3,91	7,41	4,78	0,0001735	2,83E-02	1,00									
7940147	FAM111B	1,96	3,89	7,61	4,42	0,0003714	3,65E-02	0,29									
8168264	NLGN3	1,96	3,89	7,68	4,38	0,0004104	3,84E-02	0,20									
7972289	ENST00000470538	1,91	3,76	5,69	4,33	0,0004549	3,98E-02	0,11									
7917347	DDAH1	1,90	3,74	9,08	6,07	1,25E-05	1,60E-02	3,39									
7940643	ASRGL1	1,88	3,69	7,11	5,03	0,000102	2,33E-02	1,48									
8035838	ZNF724P	1,84	3,57	7,44	5,28	6,09E-05	2,24E-02	1,95									
8046428	RAPGEF4	1,82	3,54	6,68	4,06	0,0008103	4,93E-02	-0,42									
8086799	CSPG5	1,82	3,54	6,93	4,38	0,0004093	3,84E-02	0,20									
8035318	UNC13A	1,82	3,52	6,87	4,58	0,0002634	3,25E-02	0,61									
7909841	MARK1	1,79	3,45	7,65	4,40	0,0003923	3,80E-02	0,24									
8066848	PREX1	1,78	3,44	8,97	5,10	8,94E-05	2,28E-02	1,60									
8083407	AF318318	1,77	3,41	6,55	4,47	0,0003341	3,57E-02	0,39									
7958207	BC013935	1,76	3,39	6,98	4,66	0,000226	3,17E-02	0,75									
7939383	PRR5L	1,76	3,39	7,17	4,62	0,0002415	3,17E-02	0,69									
7978391	NOVA1	1,75	3,37	8,63	4,82	0,0001598	2,72E-02	1,07									
8071086	CECR2	1,74	3,34	6,27	4,79	0,00017	2,79E-02	1,01									
8152119	NCALD	1,72	3,29	7,78	4,32	0,0004662	4,01E-02	0,08									
7978846	POLE2	1,71	3,28	6,53	4,32	0,0004645	4,01E-02	0,09									
7962274	KIF21A	1,69	3,23	9,13	4,99	0,0001112	2,36E-02	1,40									
7961900	ITPR2	1,68	3,21	8,56	4,22	0,0005742	4,37E-02	-0,11									
7985829	FANCI	1,67	3,18	9,24	4,45	0,0003504	3,62E-02	0,35									
8142405	ENST00000485160	1,62	3,07	8,70	4,12	0,0007166	4,75E-02	-0,31									
8070421	DSCAM	1,62	3,07	5,83	5,15	8,03E-05	2,24E-02	1,70									
7946340	RIC3	1,61	3,06	5,30	4,26	0,0005232	4,26E-02	-0,02									
7915982	BEND5	1,60	3,04	7,21	4,17	0,0006368	4,50E-02	-0,20									
8027247	ZNF93	1,60	3,03	7,20	6,49	5,47E-06	1,54E-02	4,13									
7982889	NUSAP1	1,59	3,02	8,85	4,43	0,0003624	3,62E-02	0,32									
7988444	MYEF2	1,58	3,00	9,43	4,76	0,000181	2,92E-02	0,96									
8005683	ENST00000516637	1,58	2,99	4,42	4,09	0,0007687	4,81E-02	-0,38									
7953211	C12ORF5	1,57	2,96	7,75	4,43	0,0003651	3,62E-02	0,31									
8027241	ZNF253	1,57	2,96	7,84	5,36	5,15E-05	2,20E-02	2,11									
8103932	MLF1IP	1,56	2,95	7,02	4,82	0,0001599	2,72E-02	1,07									
7947744	LRP4	1,55	2,92	7,97	4,37	0,0004185	3,84E-02	0,18									
7942832	C11ORF82	1,54	2,92	6,75	4,50	0,0003182	3,48E-02	0,44									
8035813	ZNF43	1,52	2,87	7,70	7,35	1,12E-06	9,96E-03	5,53									
8074789	ENST00000496490	1,52	2,87	5,41	4,59	0,0002624	3,25E-02	0,61									
7974372	GPR137C	1,50	2,83	7,75	4,68	0,0002129	3,08E-02	0,81									
7961960	ENST00000391247	1,50	2,82	5,82	4,52	0,0003016	3,39E-02	0,49									
7932214	ACBD7	1,47	2,77	6,24	4,36	0,0004265	3,87E-02	0,17									
8136473	TRIM24	1,47	2,77	10,18	4,64	0,0002355	3,17E-02	0,71									
8097586	GAB1	1,44	2,72	9,08	4,22	0,0005688	4,37E-02	-0,10									
7929438	HELLS	1,44	2,72	7,76	4,27	0,0005173	4,22E-02	-0,01									
8097857	MND1	1,43	2,70	5,92	4,97	0,000117	2,38E-02	1,36									
8157246	KIAA1958	1,42	2,68	8,02	5,07	9,40E-05	2,28E-02	1,56									
8027312	ZNF429	1,41	2,65	6,81	5,23	6,81E-05	2,24E-02	1,85									

Probe	Gene	logFC GSf vs GSr	FC GSf vs GSr	Ave Expr	t	p value	adj. p value	B	chr19p13	chr19p12	chr19q13	VER_PN	VER_CL	VER_MES	BOQUEST_STEM	WONG_STEM	GOBERT
7959025	RNFT2	1,39	2,63	8,11	4,26	0,0005314	4,27E-02	-0,04									
8025964	ZNF439	1,39	2,63	6,78	4,14	0,0006789	4,64E-02	-0,26									
8039642	VN1R1	1,39	2,62	5,61	4,84	0,0001543	2,69E-02	1,10									
8035847	ZNF675	1,38	2,61	6,59	4,98	0,0001149	2,38E-02	1,37									
7958202	CHST11	1,37	2,59	9,54	4,91	0,0001318	2,51E-02	1,25									
8035808	ZNF100	1,35	2,55	7,50	6,11	1,15E-05	1,60E-02	3,47									
7932118	BEND7	1,34	2,54	5,77	4,09	0,0007551	4,78E-02	-0,36									
7926259	MCM10	1,33	2,51	7,45	4,22	0,0005702	4,37E-02	-0,10									
8024900	UHRF1	1,32	2,50	8,29	4,33	0,0004565	3,98E-02	0,10									
8038913	ZNF649	1,32	2,50	7,60	4,92	0,0001303	2,50E-02	1,26									
8102389	C4ORF21	1,32	2,50	7,04	4,09	0,0007532	4,78E-02	-0,36									
8027292	ZNF431	1,32	2,49	7,04	5,15	8,03E-05	2,24E-02	1,70									
7983843	TCF12	1,30	2,47	10,63	5,46	4,20E-05	2,20E-02	2,29									
7976621	VRK1	1,30	2,47	8,73	5,23	6,74E-05	2,24E-02	1,86									
7983734	TMOD2	1,30	2,47	8,34	4,06	0,0008108	4,93E-02	-0,43									
7952884	B3GAT1	1,30	2,46	6,25	4,56	0,0002752	3,32E-02	0,57									
8145622	ENST00000365541	1,29	2,45	7,01	4,90	0,0001346	2,51E-02	1,23									
8129231	FAM184A	1,29	2,45	6,51	4,93	0,0001264	2,50E-02	1,29									
8035782	ZNF682	1,28	2,44	7,43	5,24	6,64E-05	2,24E-02	1,88									
8151496	ZNF704	1,27	2,42	8,03	4,37	0,0004162	3,84E-02	0,19									
8012285	KCNAB3	1,27	2,41	5,66	5,39	4,86E-05	2,20E-02	2,16									
7954077	KIAA1467	1,27	2,41	7,85	4,96	0,00012	2,42E-02	1,33									
8071212	CDC45	1,27	2,41	7,48	4,16	0,0006546	4,59E-02	-0,23									
8062766	MYBL2	1,26	2,40	8,16	4,23	0,0005648	4,37E-02	-0,09									
7906602	VANGL2	1,25	2,39	6,88	4,24	0,0005562	4,36E-02	-0,08									
8133062	ZNF273	1,25	2,38	7,58	5,49	3,95E-05	2,20E-02	2,35									
8034393	ZNF443	1,25	2,38	5,50	5,32	5,60E-05	2,20E-02	2,03									
8117640	ZSCAN16	1,25	2,38	5,90	4,31	0,0004702	4,02E-02	0,08									
8027268	ZNF66	1,24	2,36	7,13	5,11	8,73E-05	2,28E-02	1,63									
7989657	CSNK1G1	1,24	2,36	6,16	5,78	2,20E-05	1,94E-02	2,88									
8169083	ZCCHC18	1,24	2,36	5,59	4,63	0,0002372	3,17E-02	0,71									
7982792	RAD51	1,23	2,35	6,18	4,59	0,0002593	3,25E-02	0,63									
8027239	ZNF56	1,23	2,34	5,00	5,60	3,20E-05	2,15E-02	2,54									
8102371	C4ORF21	1,23	2,34	7,55	4,20	0,0006062	4,46E-02	-0,16									
8031768	ZIK1	1,22	2,33	7,64	6,28	8,21E-06	1,60E-02	3,77									
7987636	OIP5	1,18	2,27	6,81	4,56	0,0002758	3,32E-02	0,57									
7970162	ATP11A	1,17	2,24	7,54	4,35	0,000432	3,88E-02	0,16									
8046186	KLHL23	1,15	2,22	7,55	4,87	0,0001437	2,60E-02	1,17									
8086880	CDC25A	1,14	2,20	7,02	4,72	0,0001973	2,96E-02	0,88									
8021565	PHLPP1	1,14	2,20	8,15	4,20	0,0006032	4,46E-02	-0,15									
8020847	DTNA	1,13	2,19	8,67	4,19	0,0006163	4,46E-02	-0,17									
7934789	GRID1	1,13	2,19	6,39	4,66	0,000226	3,17E-02	0,75									
8133845	ENST00000410500	1,13	2,19	4,44	4,76	0,0001824	2,92E-02	0,95									
7976336	UBR7	1,13	2,19	7,99	4,99	0,0001115	2,36E-02	1,40									
8094550	C4ORF19	1,13	2,19	5,35	4,07	0,0007901	4,88E-02	-0,40									
7968212	WASF3	1,12	2,17	7,66	4,19	0,0006134	4,46E-02	-0,17									
8133057	ZNF138	1,09	2,13	7,68	5,08	9,28E-05	2,28E-02	1,57									
8092640	RFC4	1,09	2,13	8,87	4,21	0,0005814	4,40E-02	-0,12									

Probe	Gene	logFC GSf vs GSr	FC GSf vs GSr	Ave Expr	t	p value	adj. p value	B	chr19p13	chr19p12	chr19q13	VER_PN	VER_CL	VER_MES	BOQUEST_STEM	WONG_STEM	GOBERT
7984932	SCAMP5	1,09	2,12	7,37	4,61	0,000248	3,18E-02	0,67									
8027297	ZNF738	1,08	2,12	8,02	4,19	0,00061	4,46E-02	-0,16									
8035803	ZNF708	1,06	2,08	7,26	4,93	0,0001255	2,50E-02	1,29									
8005661	SPECC1	1,05	2,07	8,93	4,08	0,0007761	4,82E-02	-0,39									
8107330	APC	1,04	2,05	7,94	4,07	0,0007874	4,87E-02	-0,40									
8027304	ZNF493	1,03	2,05	6,92	4,15	0,000675	4,64E-02	-0,26									
8027272	ZNF85	1,02	2,03	7,46	5,01	0,0001076	2,34E-02	1,43									
8133049	ZNF107	1,01	2,02	7,72	6,26	8,53E-06	1,60E-02	3,73									
7916167	ORC1	1,00	2,00	6,79	4,06	0,0008053	4,93E-02	-0,42									
8139820	ZNF680	1,00	2,00	8,26	5,17	7,64E-05	2,24E-02	1,75									
8133089	ZNF92	1,00	1,99	7,37	5,30	5,90E-05	2,24E-02	1,98									
8144151	AK126609	0,99	1,99	4,68	4,13	0,0006963	4,69E-02	-0,29									
7941587	CNIH2	0,99	1,99	7,90	4,11	0,0007367	4,78E-02	-0,34									
8035842	ZNF91	0,96	1,94	8,40	5,01	0,0001066	2,34E-02	1,44									
8027285	ZNF714	0,95	1,94	7,44	4,18	0,0006325	4,50E-02	-0,20									
8039006	ZNF320	0,95	1,93	7,59	4,47	0,0003392	3,59E-02	0,38									
7897691	PTCHD2	0,95	1,93	6,20	4,34	0,0004424	3,92E-02	0,13									
8031807	ZNF551	0,95	1,93	7,44	5,75	2,34E-05	1,95E-02	2,82									
7970111	ARHGEF7	0,94	1,92	8,98	4,10	0,0007519	4,78E-02	-0,36									
8036460	DPF1	0,94	1,92	6,70	4,38	0,0004048	3,82E-02	0,22									
8026063	MAST1	0,94	1,91	6,65	4,86	0,0001465	2,62E-02	1,15									
8078999	ZNF620	0,93	1,90	6,89	4,10	0,0007531	4,78E-02	-0,36									
7907183	SCYL3	0,93	1,90	6,84	4,10	0,0007473	4,78E-02	-0,35									
8101945	H2AFZ	0,93	1,90	10,22	4,23	0,0005579	4,36E-02	-0,08									
8044882	EPB41L5	0,92	1,89	8,24	4,47	0,0003363	3,58E-02	0,39									
8025958	ZNF440	0,90	1,86	6,98	5,06	9,67E-05	2,28E-02	1,53									
7901192	RAD54L	0,89	1,85	7,29	4,41	0,0003858	3,76E-02	0,26									
8030908	ZNF480	0,89	1,85	7,85	4,90	0,0001353	2,51E-02	1,22									
8025933	ZNF833P	0,89	1,85	5,57	5,39	4,83E-05	2,20E-02	2,17									
8031815	ZNF776	0,88	1,85	8,41	4,27	0,0005116	4,21E-02	0,00									
8034315	ZNF823	0,88	1,85	6,66	5,17	7,65E-05	2,24E-02	1,75									
8025978	ZNF763	0,88	1,84	5,71	5,09	9,12E-05	2,28E-02	1,59									
8031748	ZNF749	0,87	1,83	6,68	4,36	0,0004264	3,87E-02	0,17									
7947540	TRAF6	0,87	1,82	8,70	4,99	0,0001122	2,36E-02	1,40									
8151510	ENST00000464971	0,85	1,80	5,52	4,59	0,0002625	3,25E-02	0,61									
8071332	RANBP1	0,85	1,80	9,82	4,94	0,0001229	2,46E-02	1,31									
8028200	ZNF567	0,84	1,79	5,92	4,09	0,0007629	4,80E-02	-0,37									
8027368	ZNF254	0,84	1,78	8,52	5,36	5,18E-05	2,20E-02	2,10									
8036420	ZFP30	0,83	1,78	7,74	4,65	0,0002288	3,17E-02	0,74									
8035779	ZNF253	0,82	1,77	11,29	5,17	7,69E-05	2,24E-02	1,74									
8031669	ZNF470	0,82	1,77	7,68	4,19	0,0006084	4,46E-02	-0,16									
7960383	PARP11	0,82	1,76	6,68	4,23	0,0005585	4,36E-02	-0,08									
8147101	E2F5	0,81	1,75	7,68	4,15	0,0006742	4,64E-02	-0,26									
8070046	PAXBP1	0,81	1,75	9,16	5,95	1,58E-05	1,60E-02	3,18									
7939102	ELP4	0,80	1,74	7,93	4,62	0,0002433	3,17E-02	0,68									
8033789	ZNF121	0,80	1,74	8,29	4,20	0,0006033	4,46E-02	-0,15									
7990086	THAP10	0,80	1,74	7,01	4,41	0,0003792	3,70E-02	0,28									
7950248	FCHSD2	0,78	1,71	8,45	4,62	0,0002429	3,17E-02	0,69									

Probe	Gene	logFC GSf vs GSr	FC GSf vs GSr	Ave Expr	t	p value	adj. p value	B	chr19p13	chr19p12	chr19q13	VER_PN	VER_CL	VER_MES	BOQUEST_STEM	WONG_STEM	GOBERT
8031762	ZNF549	0,77	1,71	6,62	5,32	5,57E-05	2,20E-02	2,04			■						
8034334	ZNF20	0,75	1,69	5,87	4,54	0,0002867	3,35E-02	0,53	■				■				
7959657	ATP6V0A2	0,75	1,69	8,00	4,74	0,0001904	2,95E-02	0,91									■
8021101	HAUS1	0,75	1,68	7,10	4,11	0,0007303	4,76E-02	-0,33									■
8031750	ZNF419	0,75	1,68	6,84	4,40	0,0003926	3,80E-02	0,24			■		■				
8034390	ZNF799	0,74	1,68	7,26	4,62	0,0002424	3,17E-02	0,69	■								
8025973	ZNF700	0,73	1,66	8,21	5,13	8,30E-05	2,24E-02	1,67	■								
7903334	CDC14A	0,73	1,65	7,09	5,24	6,67E-05	2,24E-02	1,87									
8013022	C17ORF76	0,72	1,64	6,64	4,10	0,0007396	4,78E-02	-0,34									■
7977646	ZNF219	0,71	1,63	6,89	4,20	0,0006043	4,46E-02	-0,15									
8024816	FSD1	0,69	1,61	7,23	4,90	0,0001349	2,51E-02	1,23	■								
8015868	MPP2	0,69	1,61	6,03	4,13	0,0007006	4,71E-02	-0,29									■
8039664	ZNF416	0,69	1,61	7,11	4,87	0,0001424	2,60E-02	1,18			■						
8031784	ZNF134	0,68	1,60	8,16	4,17	0,0006345	4,50E-02	-0,20			■		■				
8024391	DOT1L	0,68	1,60	7,89	4,27	0,0005121	4,21E-02	0,00	■								
8033801	ZNF562	0,68	1,60	7,20	4,69	0,0002096	3,07E-02	0,82	■								
7993146	ENST00000475032	0,67	1,59	3,84	4,24	0,0005449	4,32E-02	-0,06									
7953243	NDUFA9	0,67	1,59	9,02	4,85	0,0001492	2,65E-02	1,13									
8131709	SP4	0,66	1,59	8,70	4,23	0,0005678	4,37E-02	-0,10									
8031732	ZNF547	0,65	1,57	5,98	4,25	0,0005393	4,29E-02	-0,05			■						
8128075	ENST00000410213	0,64	1,56	5,82	4,22	0,0005736	4,37E-02	-0,11									
7998129	POLR3K	0,63	1,55	8,97	4,06	0,0008154	4,93E-02	-0,43									
8035236	HAUS8	0,63	1,55	6,74	4,14	0,000688	4,67E-02	-0,27	■								■
7965956	NFYB	0,61	1,53	7,68	4,19	0,0006103	4,46E-02	-0,16									
8031778	ZNF530	0,61	1,52	6,09	4,33	0,0004569	3,98E-02	0,10			■						
8027233	ZNF101	0,59	1,50	7,33	4,87	0,0001434	2,60E-02	1,17	■								
8096081	ENOPH1	0,58	1,49	10,07	4,45	0,0003472	3,62E-02	0,36									
7979663	RAB15	0,57	1,48	6,70	4,50	0,0003118	3,47E-02	0,46									
7958455	UNG	0,56	1,48	7,76	4,57	0,0002696	3,29E-02	0,59									■
8025458	ZNF317	0,56	1,47	7,78	5,54	3,56E-05	2,20E-02	2,44	■								
8033479	ELAVL1	0,55	1,47	9,09	5,18	7,51E-05	2,24E-02	1,76	■								■
8034401	ZNF564	0,55	1,46	7,41	4,19	0,0006171	4,46E-02	-0,17	■								
8033795	ZNF561	0,55	1,46	7,58	4,50	0,0003163	3,48E-02	0,44	■								
7976726	EVL	0,54	1,46	7,20	4,10	0,0007507	4,78E-02	-0,35								■	
8103235	TIGD4	0,54	1,46	6,09	4,07	0,0008033	4,93E-02	-0,42									
8031097	NDUFA3	0,54	1,45	9,43	4,38	0,0004073	3,83E-02	0,21			■						
8101411	LIN54	0,53	1,45	7,66	4,66	0,0002259	3,17E-02	0,75									■
8025927	ZNF627	0,53	1,45	7,67	4,50	0,0003149	3,48E-02	0,45	■								
7998967	ZSCAN32	0,51	1,42	6,75	4,10	0,0007481	4,78E-02	-0,35									
7991173	KLHL25	0,50	1,42	6,74	4,58	0,0002639	3,25E-02	0,61					■				
7946211	RRP8	0,50	1,41	7,14	4,54	0,000288	3,35E-02	0,53									
7959751	ZNF664	0,50	1,41	8,78	4,83	0,0001573	2,72E-02	1,09									
8027650	UBA2	0,48	1,40	9,96	4,14	0,0006826	4,64E-02	-0,27									
8090256	SNX4	0,48	1,40	9,10	4,11	0,0007288	4,76E-02	-0,33									
7951144	CCDC82	0,48	1,39	8,56	4,10	0,0007379	4,78E-02	-0,34									
7998931	ZNF200	0,46	1,38	6,65	4,73	0,0001948	2,96E-02	0,89									
7947624	PHF21A	0,45	1,36	8,44	4,19	0,0006145	4,46E-02	-0,17									
8031939	ZNF584	0,42	1,34	7,30	4,14	0,0006801	4,64E-02	-0,26			■						

Probe	Gene	logFC GSf vs GSr	FC GSf vs GSr	Ave Expr	t	p value	adj. p value	B	chr19p13	chr19p12	chr19q13	VER_PN	VER_CL	VER_MES	BOQUEST_STEM	WONG_STEM	GOBERT
8008716	MSX2P1	-0,37	-1,29	6,56	-4,39	0,0003984	3,81E-02	0,23									
7905233	ADAMTSL4	-0,37	-1,29	5,83	-4,63	0,0002364	3,17E-02	0,71									
8175900	ARHGAP4	-0,44	-1,36	6,16	-4,34	0,0004429	3,92E-02	0,13									
7929932	KAZALD1	-0,46	-1,37	6,44	-4,15	0,0006648	4,62E-02	-0,24									
8148737	MAF1	-0,48	-1,39	7,89	-4,48	0,0003277	3,53E-02	0,41									
8088054	TMEM110	-0,49	-1,40	7,82	-5,18	7,48E-05	2,24E-02	1,77									
8060225	HDLBP	-0,49	-1,40	10,83	-4,59	0,0002612	3,25E-02	0,62									
8085300	SEC13	-0,49	-1,41	8,68	-4,24	0,0005501	4,34E-02	-0,07									
8032755	ZBTB7A	-0,49	-1,41	7,94	-4,12	0,0007211	4,75E-02	-0,32									
8101376	SEC31A	-0,50	-1,41	10,47	-4,35	0,0004334	3,88E-02	0,15									
8145027	FAM160B2	-0,51	-1,42	7,50	-4,22	0,0005742	4,37E-02	-0,11									
8017867	FAM20A	-0,51	-1,42	4,73	-5,06	9,64E-05	2,28E-02	1,53									
8029854	SLC1A5	-0,51	-1,43	6,19	-4,09	0,0007671	4,81E-02	-0,37									
8082478	COPG1	-0,53	-1,44	9,33	-4,06	0,0008169	4,93E-02	-0,43									
8147970	EBAG9	-0,53	-1,44	7,18	-4,18	0,0006227	4,47E-02	-0,18									
8148824	HSF1	-0,54	-1,45	8,25	-4,63	0,0002401	3,17E-02	0,70									
8039273	CDC42EP5	-0,55	-1,46	6,57	-4,15	0,0006754	4,64E-02	-0,26									
8082380	ABTB1	-0,55	-1,46	6,52	-4,16	0,0006588	4,59E-02	-0,23									
8041197	YPEL5	-0,55	-1,47	8,13	-4,14	0,0006759	4,64E-02	-0,26									
7906898	ENST00000473793	-0,59	-1,50	5,71	-4,53	0,0002966	3,38E-02	0,50									
8009253	LOC100510740	-0,59	-1,51	5,42	-4,18	0,0006275	4,50E-02	-0,19									
8176109	SLC10A3	-0,59	-1,51	7,23	-4,30	0,0004797	4,08E-02	0,06									
8109086	ADRB2	-0,60	-1,51	5,23	-4,56	0,0002763	3,32E-02	0,57									
8054997	MAP3K2	-0,60	-1,51	8,32	-4,78	0,0001753	2,84E-02	0,99									
7899703	TXLNA	-0,61	-1,52	8,99	-4,82	0,0001594	2,72E-02	1,07									
8025199	PNPLA6	-0,61	-1,52	7,97	-5,43	4,48E-05	2,20E-02	2,23									
8017927	ABCA9	-0,63	-1,54	4,17	-4,11	0,0007246	4,76E-02	-0,32									
8019250	P4HB	-0,63	-1,55	11,12	-4,12	0,0007163	4,75E-02	-0,31									
8086467	ANO10	-0,63	-1,55	8,83	-4,49	0,0003244	3,52E-02	0,42									
8088700	TMF1	-0,64	-1,56	8,95	-4,69	0,0002089	3,07E-02	0,82									
8062023	MAP1LC3A	-0,64	-1,56	6,81	-4,51	0,0003081	3,44E-02	0,47									
8042519	PCYOX1	-0,64	-1,56	9,24	-4,07	0,0007973	4,90E-02	-0,41									
8008297	XYLT2	-0,65	-1,56	7,34	-4,13	0,000703	4,71E-02	-0,29									
7993433	PDXDC1	-0,66	-1,58	8,93	-5,08	9,21E-05	2,28E-02	1,58									
8008350	MYCBPAP	-0,67	-1,59	5,22	-5,40	4,77E-05	2,20E-02	2,18									
8002322	PDXDC2P	-0,67	-1,59	8,26	-5,43	4,51E-05	2,20E-02	2,23									
8164293	AK1	-0,68	-1,60	7,88	-4,47	0,0003332	3,57E-02	0,39									
7966035	CKAP4	-0,68	-1,60	8,57	-4,27	0,0005134	4,21E-02	0,00									
8008113	CALCOCO2	-0,68	-1,60	9,10	-4,05	0,0008283	4,98E-02	-0,45									
8013157	TOM1L2	-0,69	-1,61	6,94	-4,20	0,0006067	4,46E-02	-0,16									
8004867	NDEL1	-0,69	-1,61	9,16	-4,42	0,0003719	3,65E-02	0,29									
8111670	GDNF	-0,70	-1,62	4,44	-4,58	0,0002662	3,27E-02	0,60									
7916304	GLIS1	-0,70	-1,63	6,08	-4,23	0,0005607	4,36E-02	-0,09									
8017619	BC062608	-0,71	-1,64	3,14	-4,16	0,000657	4,59E-02	-0,23									
7905754	ATP8B2	-0,71	-1,64	8,13	-4,20	0,0006064	4,46E-02	-0,16									
7900146	ZC3H12A	-0,72	-1,65	6,78	-4,19	0,0006139	4,46E-02	-0,17									
8089759	TMEM39A	-0,72	-1,65	8,73	-4,46	0,0003457	3,62E-02	0,36									
8008087	NFE2L1	-0,73	-1,66	9,63	-4,43	0,0003625	3,62E-02	0,32									

Probe	Gene	logFC GSf vs GSr	FC GSf vs GSr	Ave Expr	t	p value	adj. p value	B	chr19p13	chr19p12	chr19q13	VER_PN	VER_CL	VER_MES	BOQUEST_STEM	WONG_STEM	GOBERT
8104201	EXOC3	-0,74	-1,67	8,10	-4,25	0,0005346	4,29E-02	-0,04									
8086572	FYCO1	-0,78	-1,71	7,73	-4,43	0,0003633	3,62E-02	0,31									■
8159566	MAN1B1	-0,78	-1,72	8,01	-4,32	0,0004644	4,01E-02	0,09									
8025053	TNFSF9	-0,79	-1,72	6,65	-4,62	0,0002415	3,17E-02	0,69	■								■
7908041	LAMC1	-0,79	-1,73	10,75	-4,14	0,0006783	4,64E-02	-0,26								■	
7956166	ESYT1	-0,80	-1,74	8,73	-4,44	0,0003605	3,62E-02	0,32									
7930682	FAM160B1	-0,81	-1,75	7,96	-4,81	0,0001645	2,76E-02	1,04									
8097038	NDST3	-0,81	-1,75	3,68	-4,27	0,0005159	4,22E-02	-0,01									
8040753	TMEM214	-0,83	-1,77	8,91	-5,37	5,10E-05	2,20E-02	2,12									
8053406	RETSAT	-0,83	-1,78	7,93	-4,13	0,0006916	4,67E-02	-0,28									
8012197	PLSCR3	-0,84	-1,79	7,03	-4,84	0,0001513	2,66E-02	1,12									
8164398	GOLGA2	-0,84	-1,79	8,50	-5,27	6,23E-05	2,24E-02	1,93									
8080926	ARL6IP5	-0,85	-1,80	10,90	-4,05	0,0008273	4,98E-02	-0,44									
8087833	DUSP7	-0,86	-1,81	8,10	-4,26	0,0005314	4,27E-02	-0,04									■
7899023	LDLRAP1	-0,86	-1,81	7,12	-4,20	0,0006036	4,46E-02	-0,15									
7914021	SLC9A1	-0,86	-1,81	7,30	-4,45	0,0003491	3,62E-02	0,35									
8149793	ENTPD4	-0,86	-1,82	9,06	-5,63	3,01E-05	2,15E-02	2,60									
8147206	RIPK2	-0,86	-1,82	7,86	-4,21	0,0005911	4,45E-02	-0,13									
7966600	SLC24A6	-0,89	-1,85	6,35	-5,07	9,52E-05	2,28E-02	1,55									
8014063	EVI2B	-0,89	-1,85	4,50	-4,30	0,0004856	4,10E-02	0,05									
8150714	PCMTD1	-0,89	-1,86	8,98	-4,28	0,0005044	4,19E-02	0,01									
7949532	FOSL1	-0,93	-1,90	7,64	-4,35	0,0004329	3,88E-02	0,15									
8146285	HGSNAT	-0,93	-1,91	8,69	-4,92	0,0001304	2,50E-02	1,26									
8006229	RNF135	-0,94	-1,91	6,19	-4,56	0,0002795	3,33E-02	0,56									
8145122	SLC39A14	-0,95	-1,93	8,39	-5,79	2,17E-05	1,94E-02	2,89							■		
8015759	VAT1	-0,95	-1,93	9,35	-4,10	0,0007393	4,78E-02	-0,34									■
7963577	SPRYD3	-0,95	-1,93	7,58	-5,16	7,80E-05	2,24E-02	1,73									
8034130	KANK2	-0,95	-1,94	8,12	-5,17	7,65E-05	2,24E-02	1,75	■								
8034698	MIR23A	-0,95	-1,94	6,07	-4,30	0,0004829	4,09E-02	0,05									
8157605	NA	-0,96	-1,95	4,99	-4,49	0,0003183	3,48E-02	0,44									
8007212	STAT5A	-0,98	-1,98	5,97	-4,71	0,0002006	3,00E-02	0,86									
7958989	PLBD2	-0,99	-1,99	7,66	-5,27	6,19E-05	2,24E-02	1,94									
8148333	EFR3A	-0,99	-1,99	9,09	-5,99	1,46E-05	1,60E-02	3,25									
8165258	AGPAT2	-0,99	-1,99	7,48	-5,02	0,0001049	2,34E-02	1,46									
8131091	MAFK	-0,99	-1,99	6,39	-4,42	0,0003702	3,65E-02	0,30									
7920664	THBS3	-1,00	-2,00	7,64	-4,12	0,0007205	4,75E-02	-0,32									
7972735	ENST00000364977	-1,00	-2,01	3,45	-4,12	0,0007105	4,75E-02	-0,30									
7970498	LATS2	-1,01	-2,01	7,16	-5,39	4,90E-05	2,20E-02	2,15								■	■
7948565	CYB561A3	-1,01	-2,01	7,19	-4,39	0,0004002	3,82E-02	0,23									
8110982	DAP	-1,01	-2,01	8,79	-4,66	0,0002256	3,17E-02	0,75									
7902702	CLCA2	-1,01	-2,02	4,16	-4,29	0,0004946	4,14E-02	0,03									
8115783	STK10	-1,02	-2,03	7,57	-4,15	0,0006743	4,64E-02	-0,26								■	
7947425	CD59	-1,04	-2,05	10,16	-4,65	0,0002307	3,17E-02	0,73									
8037005	TGFB1	-1,04	-2,06	8,09	-4,92	0,0001286	2,50E-02	1,27			■						
8034696	MIR27A	-1,05	-2,07	5,95	-4,29	0,0004957	4,14E-02	0,03									
7924499	TLR5	-1,06	-2,08	4,36	-4,64	0,0002357	3,17E-02	0,71									
7963631	RARG	-1,06	-2,08	7,28	-4,61	0,0002508	3,20E-02	0,66									
8077993	TMEM43	-1,06	-2,09	9,42	-5,20	7,22E-05	2,24E-02	1,80									

Probe	Gene	logFC GSf vs GSr	FC GSf vs GSr	Ave Expr	t	p value	adj. p value	B	chr19p13	chr19p12	chr19q13	VER_PN	VER_CL	VER_MES	BOQUEST_STEM	WONG_STEM	GOBERT
8056201	RBMS1	-1,07	-2,10	8,53	-4,10	0,0007486	4,78E-02	-0,35									
7972826	C13ORF29	-1,08	-2,11	4,84	-4,56	0,0002777	3,32E-02	0,56									
7906433	ENST00000410620	-1,08	-2,11	6,59	-5,41	4,63E-05	2,20E-02	2,20									
8028652	ZFP36	-1,09	-2,13	6,32	-4,28	0,0005038	4,19E-02	0,01									
8106170	TMEM171	-1,09	-2,13	6,04	-4,38	0,0004116	3,84E-02	0,20									
7932966	ITGB1	-1,10	-2,14	10,12	-5,07	9,48E-05	2,28E-02	1,55									
8158250	CERCAM	-1,10	-2,14	7,30	-4,69	0,0002089	3,07E-02	0,82									
7969414	KLF5	-1,11	-2,15	6,84	-4,36	0,0004248	3,87E-02	0,17									
8175444	FGF13	-1,11	-2,16	5,78	-5,11	8,63E-05	2,28E-02	1,64									
8018494	TRIM47	-1,12	-2,17	7,76	-5,03	0,0001026	2,33E-02	1,48									
7909954	DISP1	-1,12	-2,17	7,16	-4,45	0,0003533	3,62E-02	0,34									
8056005	ACVR1	-1,13	-2,19	7,41	-5,67	2,77E-05	2,06E-02	2,67									
8063382	SNAI1	-1,14	-2,21	5,75	-6,14	1,09E-05	1,60E-02	3,51									
8063386	CEBPB	-1,15	-2,21	7,52	-4,08	0,0007751	4,82E-02	-0,38									
7972055	KCTD12	-1,16	-2,23	7,59	-4,06	0,0008107	4,93E-02	-0,43									
7897803	PLOD1	-1,17	-2,25	9,31	-4,21	0,000583	4,40E-02	-0,12									
8152491	EXT1	-1,18	-2,26	7,95	-5,50	3,91E-05	2,20E-02	2,36									
8103240	ANXA2P1	-1,19	-2,28	6,93	-4,17	0,0006465	4,55E-02	-0,22									
8030128	PPP1R15A	-1,19	-2,28	7,53	-4,32	0,0004591	3,99E-02	0,10									
8023220	SMAD7	-1,19	-2,28	5,73	-4,23	0,0005616	4,36E-02	-0,09									
8070720	ICOSLG	-1,19	-2,28	6,25	-4,26	0,0005248	4,26E-02	-0,02									
7966068	ENST00000479910	-1,19	-2,28	4,24	-4,49	0,0003199	3,49E-02	0,43									
8145865	GPR124	-1,20	-2,29	6,18	-5,25	6,51E-05	2,24E-02	1,89									
8019622	TMEM106A	-1,20	-2,30	6,21	-5,75	2,34E-05	1,95E-02	2,83									
7964757	RBMS1	-1,20	-2,30	6,77	-4,55	0,0002813	3,34E-02	0,55									
8126486	CUL7	-1,22	-2,33	7,48	-5,05	9,93E-05	2,32E-02	1,51									
8017850	WIPI1	-1,23	-2,34	8,57	-4,17	0,0006371	4,50E-02	-0,20									
8102482	SEC24D	-1,24	-2,36	8,22	-4,74	0,000187	2,92E-02	0,93									
8080487	PRKCD	-1,27	-2,41	6,86	-4,33	0,0004492	3,96E-02	0,12									
8076690	C22ORF9	-1,28	-2,42	7,45	-5,23	6,80E-05	2,24E-02	1,85									
8138442	TWIST1	-1,28	-2,43	7,75	-4,68	0,0002139	3,08E-02	0,80									
8157804	OLFML2A	-1,29	-2,45	6,93	-4,53	0,0002924	3,38E-02	0,51									
8037374	PLAUR	-1,31	-2,47	7,10	-4,51	0,0003067	3,44E-02	0,47									
8179103	HLA-E	-1,31	-2,48	8,95	-4,53	0,0002961	3,38E-02	0,50									
7922130	DPT	-1,31	-2,48	5,12	-4,44	0,0003595	3,62E-02	0,32									
8052669	SERTAD2	-1,33	-2,51	7,66	-5,49	3,97E-05	2,20E-02	2,35									
7936115	SH3PXD2A	-1,33	-2,52	6,31	-4,45	0,0003524	3,62E-02	0,34									
8075728	MYH9	-1,34	-2,54	9,26	-4,43	0,0003657	3,62E-02	0,31									
8038407	RRAS	-1,38	-2,60	8,24	-6,07	1,24E-05	1,60E-02	3,40									
8180300	TWIST2	-1,38	-2,61	7,31	-6,11	1,16E-05	1,60E-02	3,46									
8180372	NM_006456.1	-1,39	-2,62	5,94	-4,65	0,0002298	3,17E-02	0,74									
7913787	C1ORF201	-1,40	-2,63	6,30	-4,11	0,0007299	4,76E-02	-0,33									
7994659	MVP	-1,41	-2,65	7,24	-5,10	8,80E-05	2,28E-02	1,62									
7927723	C10ORF107	-1,42	-2,67	3,70	-5,81	2,10E-05	1,94E-02	2,92									
7910111	EPHX1	-1,42	-2,68	8,32	-4,06	0,0008159	4,93E-02	-0,43									
8150988	ASPH	-1,43	-2,69	8,78	-5,45	4,27E-05	2,20E-02	2,28									
8109093	ABLIM3	-1,44	-2,71	6,51	-6,40	6,53E-06	1,54E-02	3,97									
8037408	KCNN4	-1,45	-2,73	6,78	-4,24	0,0005499	4,34E-02	-0,07									

Probe	Gene	logFC GSf vs GSr	FC GSf vs GSr	Ave Expr	t	p value	adj. p value	B	chr19p13	chr19p12	chr19q13	VER_PN	VER_CL	VER_MES	BOQUEST_STEM	WONG_STEM	GOBERT
8011027	MYO1C	-1,46	-2,75	8,08	-6,29	8,04E-06	1,60E-02	3,79									
8027778	FXYD5	-1,48	-2,79	6,79	-4,53	0,0002961	3,38E-02	0,50									
7977046	TNFAIP2	-1,48	-2,80	6,67	-4,06	0,0008141	4,93E-02	-0,43									
7981161	C14ORF49	-1,51	-2,85	6,40	-5,15	8,04E-05	2,24E-02	1,70									
8013384	ALDH3A1	-1,53	-2,89	6,98	-4,31	0,0004726	4,03E-02	0,07									
8008646	SCPEP1	-1,54	-2,91	8,21	-4,74	0,0001875	2,92E-02	0,92									
7945663	LOC402778	-1,56	-2,94	6,52	-4,33	0,0004537	3,98E-02	0,11									
8159142	COL5A1	-1,59	-3,01	6,56	-4,73	0,000191	2,95E-02	0,91									
8040792	EMILIN1	-1,59	-3,01	7,31	-6,01	1,40E-05	1,60E-02	3,29									
8020141	APCDD1	-1,60	-3,03	7,50	-5,03	0,0001031	2,33E-02	1,47									
8041048	FOSL2	-1,60	-3,03	7,92	-4,79	0,000169	2,79E-02	1,02									
7919800	CTSS	-1,62	-3,08	5,76	-4,37	0,000419	3,84E-02	0,18									
8153568	PLEC	-1,63	-3,09	8,60	-4,97	0,0001165	2,38E-02	1,36									
8034694	MIR24-2	-1,63	-3,10	4,46	-4,24	0,0005522	4,34E-02	-0,07									
8114010	IRF1	-1,64	-3,11	6,79	-4,12	0,0007189	4,75E-02	-0,31									
8156228	CTSL1	-1,64	-3,12	8,62	-4,53	0,0002981	3,38E-02	0,50									
7924996	C1ORF198	-1,73	-3,31	7,99	-5,06	9,61E-05	2,28E-02	1,54									
7906085	LMNA	-1,73	-3,32	8,28	-5,13	8,35E-05	2,24E-02	1,67									
7953040	CACNA1C	-1,75	-3,35	5,70	-4,43	0,0003652	3,62E-02	0,31									
8150881	PLAG1	-1,76	-3,38	5,36	-4,92	0,0001299	2,50E-02	1,26									
8164217	FAM129B	-1,77	-3,41	8,57	-4,87	0,0001446	2,60E-02	1,16									
8160637	B4GALT1	-1,79	-3,45	7,72	-4,18	0,0006291	4,50E-02	-0,19									
8016390	COPZ2	-1,81	-3,50	7,39	-4,75	0,000184	2,92E-02	0,94									
8130556	SOD2	-1,81	-3,50	9,27	-4,84	0,0001516	2,66E-02	1,12									
8120043	RUNX2	-1,84	-3,57	7,08	-4,75	0,0001848	2,92E-02	0,94									
8163896	STOM	-1,86	-3,63	8,11	-4,63	0,0002393	3,17E-02	0,70									
8163002	KLF4	-1,87	-3,66	6,44	-4,28	0,0005081	4,21E-02	0,01									
8097288	FAT4	-1,87	-3,66	5,45	-5,14	8,14E-05	2,24E-02	1,69									
8040238	HPCAL1	-1,88	-3,67	8,58	-7,85	4,70E-07	6,64E-03	6,28									
7950005	MRGPRF	-1,89	-3,69	6,32	-5,20	7,26E-05	2,24E-02	1,79									
8009243	C17ORF60	-1,90	-3,74	5,17	-4,39	0,0003949	3,80E-02	0,24									
8066513	SDC4	-1,93	-3,80	8,45	-4,08	0,0007714	4,81E-02	-0,38									
8113220	ELL2	-1,93	-3,81	7,07	-4,45	0,0003538	3,62E-02	0,34									
7951372	CASP4	-1,93	-3,82	6,08	-4,34	0,0004422	3,92E-02	0,13									
8021946	COLEC12	-1,94	-3,82	5,51	-4,62	0,0002427	3,17E-02	0,69									
7995681	MMP2	-1,96	-3,89	8,78	-4,66	0,0002256	3,17E-02	0,75									
8034643	CACNA1A	-1,97	-3,91	6,40	-4,08	0,0007721	4,81E-02	-0,38									
8060997	SPTLC3	-1,99	-3,97	5,09	-5,57	3,36E-05	2,20E-02	2,50									
7933194	CXCL12	-2,01	-4,02	5,56	-4,18	0,0006206	4,47E-02	-0,18									
8094609	FAM114A1	-2,04	-4,11	8,03	-4,48	0,0003257	3,52E-02	0,42									
8104321	ADAMTS16	-2,04	-4,11	6,41	-4,88	0,0001403	2,59E-02	1,19									
8004510	CD68	-2,07	-4,20	7,68	-5,60	3,20E-05	2,15E-02	2,54									
8081386	NFKBIZ	-2,08	-4,24	7,08	-4,31	0,00047	4,02E-02	0,08									
8147469	CPQ	-2,11	-4,32	7,20	-4,52	0,0003007	3,39E-02	0,49									
8113981	P4HA2	-2,14	-4,40	8,14	-4,97	0,000116	2,38E-02	1,36									
8170648	BGN	-2,20	-4,60	7,09	-5,41	4,69E-05	2,20E-02	2,19									
8160260	BNC2	-2,23	-4,68	6,30	-4,80	0,0001658	2,77E-02	1,04									
8056151	PLA2R1	-2,25	-4,75	6,22	-4,30	0,0004872	4,11E-02	0,04									

Probe	Gene	logFC GSf vs GSr	FC GSf vs GSr	Ave Expr	t	p value	adj. p value	B	chr19p13	chr19p12	chr19q13	VER_PN	VER_CL	VER_MES	BOQUEST_STEM	WONG_STEM	GOBERT
7920271	S100A4	-2,31	-4,98	6,44	-4,59	0,0002621	3,25E-02	0,62									
8118734	ITPR3	-2,33	-5,03	6,90	-5,70	2,58E-05	2,02E-02	2,74									
7963786	ITGA5	-2,35	-5,10	8,21	-4,39	0,0004018	3,82E-02	0,22									
7986214	SLCO3A1	-2,36	-5,12	7,46	-4,54	0,000288	3,35E-02	0,53									
7981157	C14ORF139	-2,37	-5,17	6,17	-5,52	3,75E-05	2,20E-02	2,40									
7902452	AK5	-2,41	-5,30	4,68	-5,03	0,0001015	2,33E-02	1,49									
7914557	SYNC	-2,49	-5,60	7,42	-5,97	1,51E-05	1,60E-02	3,22									
8122099	ENPP1	-2,53	-5,77	6,43	-5,91	1,72E-05	1,67E-02	3,11									
8049752	SNED1	-2,55	-5,84	6,69	-4,81	0,0001636	2,76E-02	1,05									
8069269	COL6A1	-2,57	-5,92	9,94	-5,62	3,06E-05	2,15E-02	2,58									
8069301	COL6A2	-2,59	-6,02	8,73	-6,40	6,53E-06	1,54E-02	3,97									
8043995	IL1R1	-2,61	-6,12	4,74	-4,53	0,0002976	3,38E-02	0,50									
7988467	FBN1	-2,66	-6,34	7,86	-5,36	5,20E-05	2,20E-02	2,10									
7948667	AHNAK	-2,71	-6,53	8,77	-4,64	0,0002329	3,17E-02	0,72									
8089145	ABI3BP	-2,71	-6,54	5,27	-4,25	0,0005371	4,29E-02	-0,05									
8149774	LOXL2	-2,76	-6,77	8,03	-6,90	2,54E-06	1,02E-02	4,81									
8172266	MIR221	-2,78	-6,85	6,58	-4,13	0,0006919	4,67E-02	-0,28									
8151816	GEM	-2,86	-7,24	9,04	-5,13	8,25E-05	2,24E-02	1,68									
8116418	GFPT2	-3,01	-8,05	7,15	-4,35	0,0004341	3,88E-02	0,15									
7935058	MYOF	-3,02	-8,13	8,50	-4,39	0,0003964	3,81E-02	0,23									
8127563	COL12A1	-3,03	-8,16	6,00	-4,46	0,0003449	3,62E-02	0,36									
8051583	CYP1B1	-3,09	-8,52	7,23	-4,09	0,0007612	4,80E-02	-0,37									
7922976	PTGS2	-3,09	-8,54	5,96	-4,98	0,0001149	2,38E-02	1,37									
7906900	DDR2	-3,10	-8,55	7,96	-4,72	0,0001962	2,96E-02	0,88									
7906878	DDR2	-3,10	-8,59	8,72	-4,54	0,000286	3,35E-02	0,54									
8152703	FBXO32	-3,12	-8,71	7,67	-6,73	3,51E-06	1,10E-02	4,52									
7952341	CLMP	-3,14	-8,81	7,82	-6,08	1,22E-05	1,60E-02	3,42									
8135601	MET	-3,18	-9,08	6,07	-4,10	0,0007443	4,78E-02	-0,35									
7981514	AHNAK2	-3,18	-9,08	7,85	-4,90	0,0001348	2,51E-02	1,23									
8134869	PCOLCE	-3,33	-10,06	8,73	-5,73	2,46E-05	1,99E-02	2,78									
7991335	ANPEP	-3,36	-10,26	6,75	-6,10	1,16E-05	1,60E-02	3,46									
8112980	EDIL3	-3,51	-11,41	5,63	-4,09	0,0007598	4,80E-02	-0,37									
8102792	PCDH18	-3,53	-11,57	7,50	-5,14	8,09E-05	2,24E-02	1,69									
7961514	MGP	-3,58	-11,93	6,79	-4,38	0,0004044	3,82E-02	0,22									
8111772	DAB2	-3,72	-13,13	6,05	-7,93	4,09E-07	6,64E-03	6,40									
8063536	TFAP2C	-3,87	-14,67	6,59	-7,10	1,76E-06	9,96E-03	5,13									
8120719	CD109	-4,01	-16,12	7,54	-5,33	5,55E-05	2,20E-02	2,04									
8046922	COL3A1	-4,22	-18,63	7,52	-4,70	0,0002056	3,06E-02	0,84									
8131844	GPNMB	-4,58	-23,84	7,43	-5,29	5,92E-05	2,24E-02	1,98									
8134263	COL1A2	-5,43	-43,13	8,76	-7,12	1,70E-06	9,96E-03	5,16									

Supplementary Table S1b. Gene Set Enrichment Results

MSigDB v5.0 — Link

Positional Gene Sets

Gene Set Name	# Genes in Gene Set (K)	Description	# Genes in Overlap (k)	k/K	p-value	FDR q-value
chr19p13	645	Genes in cytogenetic band chr19p13	35	0,0543	1,51E-16	4,93E-14
chr19p12	62	Genes in cytogenetic band chr19p12	13	0,2097	2,17E-14	3,54E-12
chr19q13	948	Genes in cytogenetic band chr19q13	28	0,0295	1,49E-07	1,62E-05
chr1q12	27	Genes in cytogenetic band chr1q12	4	0,1481	1,16E-04	9,45E-03

Curated Gene sets

Gene Set Name	# Genes in Gene Set (K)	Description	# Genes in Overlap (k)	k/K	p-value	FDR q-value
CHICAS_RB1_TARGETS_CONFLUENT	567	Genes up-regulated in confluent IMR90 cells (fibroblast) after knockdown of RB1 [GeneID=5925] by RNAi.	43	0,0758	1,12E-25	5,28E-22
REACTOME_GENERIC_TRANSCRIPTION_PATHWAY	352	Genes involved in Generic Transcription Pathway	35	0,0994	5,52E-25	1,31E-21
REN_ALVEOLAR_RHABDOMYOSARCOMA_DN	408	Genes commonly down-regulated in human alveolar rhabdomyosarcoma (ARMS) and its mouse model overexpressing PAX3-FOXO1 [GeneID=5077;2308] fusion.	36	0,0882	7,04E-24	1,11E-20
MEISSNER_BRAIN_HCP_WITH_H3K4ME3_AND_H3K27ME3	1069	Genes with high-CpG-density promoters (HCP) bearing histone H3 dimethylation at K4 (H3K4me2) and trimethylation at K27 (H3K27me3) in brain.	50	0,0468	2,09E-20	2,47E-17
BOQUEST_STEM_CELL_UP	260	Genes up-regulated in freshly isolated CD31- [GeneID=5175] (stromal stem cells from adipose tissue) versus the CD31+ (non-stem) counterparts.	26	0,1	6,28E-19	5,93E-16
DODD_NASOPHARYNGEAL_CARCINOMA_DN	1375	Genes down-regulated in nasopharyngeal carcinoma (NPC) compared to the normal tissue.	53	0,0385	6,40E-18	5,00E-15
BLALOCK_ALZHEIMERS_DISEASE_UP	1691	Genes up-regulated in brain from patients with Alzheimer's disease.	59	0,0349	7,41E-18	5,00E-15
SCHUETZ_BREAST_CANCER_DUCTAL_INVASIVE_UP	351	Genes up-regulated in invasive ductal carcinoma (IDC) relative to ductal carcinoma in situ (DCIS, non-invasive).	28	0,0798	1,11E-17	6,58E-15
ZWANG_TRANSIENTLY_UP_BY_1ST_EGF_PULSE_ONLY	1839	Genes transiently induced only by the first pulse of EGF [GeneID =1950] in 184A1 cells (mammary epithelium).	61	0,0332	2,08E-17	1,09E-14
CHARAFE_BREAST_CANCER_LUMINAL_VS_MESENCHYMAL_DN	460	Genes down-regulated in luminal-like breast cancer cell lines compared to the mesenchymal-like ones.	31	0,0674	2,36E-17	1,11E-14
GOBERT_OLIGODENDROCYTE_DIFFERENTIATION_DN	1080	Genes down-regulated during differentiation of Oli-Neu cells (oligodendroglial precursor) in response to PD174265 [PubChemID=4709].	45	0,0417	1,41E-16	5,65E-14
DUTERTRE_ESTRADIOL_RESPONSE_24HR_UP	324	Genes up-regulated in MCF7 cells (breast cancer) at 24 h of estradiol [PubChemID=5757] treatment.	26	0,0802	1,44E-16	5,65E-14
VERHAAK_GLIOBLASTOMA_MESENCHYMAL	216	Genes correlated with mesenchymal type of glioblastoma multiforme tumors.	22	0,1019	2,20E-16	7,98E-14
ONDER_CDH1_TARGETS_2_UP	256	Genes up-regulated in HMLE cells (immortalized nontransformed mammary epithelium) after E-cadhedrin (CDH1) [GeneID=999] knockdown by RNAi.	23	0,0898	7,20E-16	2,43E-13
PICCALUGA_ANGIOIMMUNOBLASTIC_LYMPHOMA_UP	205	Up-regulated genes in angioimmunoblastic lymphoma (AILT) compared to normal T lymphocytes.	21	0,1024	9,45E-16	2,98E-13
WONG_ADULT_TISSUE_STEM_MODULE	721	The 'adult tissue stem' module: genes coordinately up-regulated in a compendium of adult tissue stem cells.	35	0,0485	4,61E-15	1,36E-12
KOBAYASHI_EGFR_SIGNALING_24HR_DN	251	Genes down-regulated in H1975 cells (non-small cell lung cancer, NSCLC) resistant to gefitinib [PubChem=123631] after treatment with EGFR inhibitor CL-387785 [PubChem=2776] for 24h.	22	0,0876	5,18E-15	1,44E-12
CHEN_METABOLIC_SYNDROM_NETWORK	1210	Genes forming the macrophage-enriched metabolic network (MEMN) claimed to have a causal relationship with the metabolic syndrom traits.	45	0,0372	8,63E-15	2,21E-12
GOZGIT_ESR1_TARGETS_DN	781	Genes down-regulated in TMX2-28 cells (breast cancer) which do not express ESR1 [GeneID=2099]) compared to the parental MCF7 cells which do.	36	0,0461	8,87E-15	2,21E-12
ONKEN_UVEAL_MELANOMA_UP	783	Genes up-regulated in uveal melanoma: class 2 vs class 1 tumors.	36	0,046	9,58E-15	2,26E-12
MASSARWEH_TAMOXIFEN_RESISTANCE_UP	578	Genes up-regulated in breast cancer tumors (formed by MCF-7 xenografts) resistant to tamoxifen [PubChem=5376].	31	0,0536	1,26E-14	2,82E-12
KINSEY_TARGETS_OF_EWSR1_FLII_FUSION_UP	1278	Genes up-regulated in TC71 and EWS502 cells (Ewing's sarcoma) by EWSR1-FLI1 [GeneID=2130;2314] as inferred from RNAi knockdown of this fusion protein.	46	0,036	1,39E-14	2,98E-12
GEORGES_TARGETS_OF_MIR192_AND_MIR215	893	Genes down-regulated in HCT116 cells (colon cancer) by expression of MIR192 or MIR215 [GeneID=406967;406997] at 24 h.	38	0,0426	1,92E-14	3,94E-12
PHONG_TNF_RESPONSE_NOT_VIA_P38	337	Genes whose expression changes in Calu-6 cells (lung cancer) by TNF [GeneID=7124] were not affected by p38 inhibitor LY479754.	24	0,0712	3,03E-14	5,97E-12
BERENJENO_TRANSFORMED_BY_RHOA_UP	536	Genes up-regulated in NIH3T3 cells (fibroblasts) transformed by expression of contitutively active (Q63L) form of RHOA [GeneID=387] off plasmid vector.	29	0,0541	7,37E-14	1,39E-11
BRUINS_UVC_RESPONSE_LATE	1137	Late response genes: differentially expressed only 12 h after UV-C irradiation of MEF cells (embryonic fibroblast).	42	0,0369	8,94E-14	1,63E-11
MILI_PSEUDOPODIA_HAPTOTAXIS_DN	668	Transcripts depleted from pseudopodia of NIH/3T3 cells (fibroblast) in response to haptotactic migratory stimulus by fibronectin, FN1 [GeneID=2335].	32	0,0479	1,02E-13	1,79E-11
LINDGREN_BLADDER_CANCER_CLUSTER_2B	392	Genes specifically up-regulated in Cluster IIb of urothelial cell carcinom (UCC) tumors.	25	0,0638	1,07E-13	1,80E-11
BUYTAERT_PHOTODYNAMIC_THERAPY_STRESS_UP	811	Genes up-regulated in T24 (bladder cancer) cells in response to the photodynamic therapy (PDT) stress.	35	0,0432	1,44E-13	2,34E-11
LIU_PROSTATE_CANCER_DN	481	Genes down-regulated in prostate cancer samples.	27	0,0561	2,29E-13	3,61E-11
CHICAS_RB1_TARGETS_SENESCENT	572	Genes up-regulated in senescent IMR90 cells (fibroblast) after knockdown of RB1 [GeneID=5925] by RNAi.	29	0,0507	3,74E-13	5,70E-11
LIAO_METASTASIS	539	Genes up-regulated in the samples with intrahepatic metastatic hepatocellular carcinoma (HCC) vs primary HCC.	28	0,0519	5,36E-13	7,91E-11
BILD_HRAS_ONCOGENIC_SIGNATURE	261	Genes selected in supervised analyses to discriminate cells expressing activated HRAS [GeneID=3265] oncogene from control cells expressing GFP.	20	0,0766	1,12E-12	1,61E-10
SENGUPTA_NASOPHARYNGEAL_CARCINOMA_UP	294	Genes up-regulated in nsopharyngeal carcinoma relative to the normal tissue.	21	0,0714	1,18E-12	1,64E-10
KINSEY_TARGETS_OF_EWSR1_FLII_FUSION_DN	329	Genes down-regulated in TC71 and EWS502 cells (Ewing's sarcoma) by EWSR1-FLI1 [GeneID=2130;2314] as inferred from RNAi knockdown of this fusion protein.	22	0,0669	1,28E-12	1,73E-10
CUI_TCF21_TARGETS_2_DN	830	All significantly down-regulated genes in kidney glomeruli isolated from TCF21 [Gene ID=6943] knockout mice.	34	0,041	1,38E-12	1,81E-10
VERHAAK_GLIOBLASTOMA_PRONEURAL	177	Genes correlated with proneural type of glioblastoma multiforme tumors.	17	0,096	1,51E-12	1,93E-10
HOSHIDA_LIVER_CANCER_SUBCLASS_S1	237	Genes from 'subtype S1' signature of hepatocellular carcinoma (HCC): aberrant activation of the WNT signaling pathway.	19	0,0802	1,87E-12	2,26E-10
LEE_BMP2_TARGETS_UP	745	Genes up-regulated in uterus upon knockout of BMP2 [GeneID=650].	32	0,043	1,87E-12	2,26E-10
FOSTER_TOLERANT_MACROPHAGE_DN	409	Class NT (non-tolerizeable) genes: induced during the first LPS stimulation and induced at equal or greater degree in tolerant macrophages.	24	0,0587	1,97E-12	2,30E-10
RODWELL_AGING_KIDNEY_UP	487	Genes whose expression increases with age in normal kidney.	26	0,0534	1,99E-12	2,30E-10
GRAESSMANN_APOPTOSIS_BY_DOXORUBICIN_DN	1781	Genes down-regulated in ME-A cells (breast cancer) undergoing apoptosis in response to doxorubicin [PubChem=31703].	51	0,0286	2,91E-12	3,27E-10
MILI_PSEUDOPODIA_CHEMOTAXIS_DN	457	Transcripts depleted in pseudopodia of NIH/3T3 cells (fibroblast) in response to the chemotactic migration stimulus by lysophosphatidic acid (LPA) [PubChem=3988].	25	0,0547	3,15E-12	3,46E-10
WANG_SMARCE1_TARGETS_UP	280	Genes up-regulated in BT549 cells (breast cancer) by expression of SMARCE1 [GeneID=6605] off a retroviral vector.	20	0,0714	4,09E-12	4,39E-10
NUYTTEN_EZH2_TARGETS_DN	1024	Genes down-regulated in PC3 cells (prostate cancer) after knockdown of EZH2 [GeneID=2146] by RNAi.	37	0,0361	5,46E-12	5,73E-10
NABA_MATRISOME	1028	Ensemble of genes encoding extracellular matrix and extracellular matrix-associated proteins	37	0,036	6,11E-12	6,28E-10
NUYTTEN_EZH2_TARGETS_UP	1037	Genes up-regulated in PC3 cells (prostate cancer) after knockdown of EZH2 [GeneID=2146] by RNAi.	37	0,0357	7,85E-12	7,78E-10
RUIZ_TNC_TARGETS_DN	142	Genes down-regulated in T98G cells (glioblastoma) by TNC [GeneID=3371].	15	0,1056	7,91E-12	7,78E-10
ZHANG_TLX_TARGETS_60HR_UP	293	Genes up-regulated in neural stem cells (NSC) at 60 h after cre-lox knockout of TLX (NR2E1) [GeneID=7101].	20	0,0683	9,35E-12	9,02E-10
DOUGLAS_BMI1_TARGETS_UP	566	Genes up-regulated in A4573 cells (Ewing's sarcoma, ESFT) after knockdown of BMI1 [GeneID=648] by RNAi.	27	0,0477	9,96E-12	9,42E-10

Computational Gene sets

Gene Set Name	# Genes in Gene Set (K)	Description	# Genes in Overlap (k)	k/K	p-value	FDR q-value
MODULE_5	434	Genes in the cancer module 5	26	0,0599	1,45E-13	1,24E-10
GNF2_CDH11	25	Neighborhood of CDH11	9	0,36	1,00E-12	4,31E-10
MODULE_1	368	Genes in the cancer module 1	23	0,0625	1,58E-12	4,53E-10
MODULE_38	465	Genes in the cancer module 38	25	0,0538	4,59E-12	9,85E-10
MODULE_47	225	Genes in the cancer module 47	18	0,08	7,43E-12	1,27E-09
GNF2_PTX3	36	Neighborhood of PTX3	9	0,25	4,22E-11	6,03E-09
MODULE_321	112	Genes in the cancer module 321	13	0,1161	5,96E-11	7,31E-09
MODULE_45	583	Genes in the cancer module 45	26	0,0446	1,05E-10	1,12E-08
MODULE_6	416	Genes in the cancer module 6	22	0,0529	1,22E-10	1,17E-08
MODULE_12	360	Genes in the cancer module 12	20	0,0556	3,65E-10	3,14E-08
MODULE_44	327	Genes in the cancer module 44	18	0,055	3,22E-09	2,51E-07
MODULE_84	549	Genes in the cancer module 84	22	0,0401	1,99E-08	1,43E-06
MODULE_2	384	Genes in the cancer module 2	18	0,0469	3,82E-08	2,52E-06
MODULE_55	834	Genes in the cancer module 55	27	0,0324	4,23E-08	2,59E-06
MODULE_98	393	Genes in the cancer module 98	18	0,0458	5,41E-08	3,10E-06

MODULE_24	453	Genes in the cancer module 24	19	0,0419	9,18E-08	4,92E-06
MODULE_122	141	Genes in the cancer module 122	11	0,078	1,18E-07	5,94E-06
MODULE_198	303	Genes in the cancer module 198	15	0,0495	2,60E-07	1,17E-05
GNF2_PCNA	68	Neighborhood of PCNA	8	0,1176	2,72E-07	1,17E-05
MODULE_54	263	Genes in the cancer module 54	14	0,0532	2,72E-07	1,17E-05
MODULE_128	98	Genes in the cancer module 128	9	0,0918	4,20E-07	1,72E-05
MODULE_79	100	Genes in the cancer module 79	9	0,09	5,00E-07	1,93E-05
MODULE_252	237	Genes in the cancer module 252	13	0,0549	5,17E-07	1,93E-05
MODULE_170	101	Genes in the cancer module 170	9	0,0891	5,44E-07	1,94E-05
MODULE_16	511	Genes in the cancer module 16	19	0,0372	5,74E-07	1,97E-05
MODULE_88	838	Genes in the cancer module 88	25	0,0298	6,09E-07	2,01E-05
MODULE_60	425	Genes in the cancer module 60	17	0,04	8,37E-07	2,66E-05
MORF_ATRX	211	Neighborhood of ATRX	12	0,0569	9,67E-07	2,96E-05
GNF2_RFC4	60	Neighborhood of RFC4	7	0,1167	1,63E-06	4,81E-05
MODULE_18	451	Genes in the cancer module 18	17	0,0377	1,87E-06	5,36E-05
MODULE_53	405	Genes in the cancer module 53	16	0,0395	2,06E-06	5,70E-05
MODULE_19	314	Genes in the cancer module 19	14	0,0446	2,23E-06	5,98E-05
GCM_AQP4	45	Neighborhood of AQP4	6	0,1333	4,14E-06	1,06E-04
MORF_PSMF1	164	Neighborhood of PSMF1	10	0,061	4,18E-06	1,06E-04
MODULE_3	385	Genes in the cancer module 3	15	0,039	5,06E-06	1,24E-04
MODULE_15	358	Genes in the cancer module 15	14	0,0391	9,98E-06	2,38E-04
GNF2_MMP1	32	Neighborhood of MMP1	5	0,1562	1,21E-05	2,81E-04
MORF_RUNX1	150	Neighborhood of RUNX1	9	0,06	1,44E-05	3,25E-04
GNF2_FEN1	56	Neighborhood of FEN1	6	0,1071	1,51E-05	3,33E-04
MODULE_11	540	Genes in the cancer module 11	17	0,0315	1,96E-05	4,21E-04
MODULE_100	544	Genes in the cancer module 100	17	0,0312	2,15E-05	4,44E-04
MODULE_33	384	Genes in the cancer module 33	14	0,0365	2,17E-05	4,44E-04
MODULE_137	546	Genes in the cancer module 137	17	0,0311	2,26E-05	4,50E-04
MODULE_105	200	Genes in the cancer module 105	10	0,05	2,37E-05	4,62E-04
MODULE_66	552	Genes in the cancer module 66	17	0,0308	2,59E-05	4,92E-04
MORF_MT4	246	Neighborhood of MT4	11	0,0447	2,64E-05	4,92E-04
GNF2_CENPF	62	Neighborhood of CENPF	6	0,0968	2,73E-05	4,98E-04
MODULE_49	294	Genes in the cancer module 49	12	0,0408	2,81E-05	5,03E-04
GNF2_RFC3	41	Neighborhood of RFC3	5	0,122	4,20E-05	7,36E-04
MODULE_13	519	Genes in the cancer module 13	16	0,0308	4,39E-05	7,45E-04

Motif Gene sets

Gene Set Name	# Genes in Gene Set (K)	Description	# Genes in Overlap (k)	k/K	p-value	FDR q-value
GGGCGGR_V$SP1_Q6	2940	Genes with promoter regions [-2kb,2kb] around transcription start site containing the motif GGGCGGR which matches annotation for SP1: Sp1 transcription factor	89	0,0303	1,09E-22	9,10E-20
AACTTT_UNKNOWN	1890	Genes with promoter regions [-2kb,2kb] around transcription start site containing motif AACTTT. Motif does not match any known transcription factor	64	0,0339	1,11E-18	4,63E-16
GGGAGGRR_V$MAZ_Q6	2274	Genes with promoter regions [-2kb,2kb] around transcription start site containing the motif GGGAGGRR which matches annotation for MAZ: MYC-associated zinc finger protein (purine-binding transcription factor)	69	0,0303	1,28E-17	3,56E-15
TTGTTT_V$FOXO4_01	2061	Drosophila); translocated to, 7	63	0,0306	2,83E-16	5,91E-14
TGGAAA_V$NFAT_Q4_01	1896	Genes with promoter regions [-2kb,2kb] around transcription start site containing the motif TGGAAA which matches annotation for NFAT<br>NFATC	59	0,0311	1,28E-15	2,14E-13
TGTTTAC,MIR-30A-5P,MIR-30C,MIR-30D,MIR-30B,MIR-30E-5P	579	Targets of MicroRNA TGTTTAC,MIR-30A-5P,MIR-30C,MIR-30D,MIR-30B,MIR-30E-5P	32	0,0553	2,01E-15	2,80E-13
CTTTGT_V$LEF1_Q2	1972	Genes with promoter regions [-2kb,2kb] around transcription start site containing the motif CTTTGT which matches annotation for LEF1: lymphoid enhancer-binding factor 1	58	0,0294	2,56E-14	3,05E-12
GGGTGGRR_V$PAX4_03	1294	Genes with promoter regions [-2kb,2kb] around transcription start site containing the motif GGGTGGRR which matches annotation for PAX4: paired box gene 4	44	0,034	3,76E-13	3,92E-11
TGCCTTA,MIR-124A	552	Targets of MicroRNA TGCCTTA,MIR-124A	28	0,0507	9,49E-13	8,82E-11
GTGCCTT,MIR-506	727	Targets of MicroRNA GTGCCTT,MIR-506	31	0,0426	5,04E-12	4,22E-10
CAGGTG_V$E12_Q6	2485	Genes with promoter regions [-2kb,2kb] around transcription start site containing the motif CAGGTG which matches annotation for TCF3: transcription factor 3 (E2A immunoglobulin enhancer binding factors E12/E47)	60	0,0241	3,34E-11	2,54E-09
CTTTGA_V$LEF1_Q2	1232	Genes with promoter regions [-2kb,2kb] around transcription start site containing the motif CTTTGA which matches annotation for LEF1: lymphoid enhancer-binding factor 1	38	0,0308	2,67E-10	1,76E-08
TGANTCA_V$AP1_C	1121	Genes with promoter regions [-2kb,2kb] around transcription start site containing the motif TGANTCA which matches annotation for JUN: jun oncogene	36	0,0321	2,74E-10	1,76E-08
TATTATA,MIR-374	284	Targets of MicroRNA TATTATA,MIR-374	18	0,0634	3,43E-10	2,05E-08
ACCAAAG,MIR-9	499	Targets of MicroRNA ACCAAAG,MIR-9	23	0,0461	6,77E-10	3,77E-08
YCATTAA_UNKNOWN	556	Genes with promoter regions [-2kb,2kb] around transcription start site containing motif YCATTAA. Motif does not match any known transcription factor	24	0,0432	1,06E-09	5,52E-08
GTGCAAT,MIR-25,MIR-32,MIR-92,MIR-363,MIR-367	311	Targets of MicroRNA GTGCAAT,MIR-25,MIR-32,MIR-92,MIR-363,MIR-367	18	0,0579	1,46E-09	7,19E-08
TTTGCAC,MIR-19A,MIR-19B	516	Targets of MicroRNA TTTGCAC,MIR-19A,MIR-19B	22	0,0426	6,59E-09	3,06E-07
V$CEBP_C	200	Genes with promoter regions [-2kb,2kb] around transcription start site containing the motif NGWVTKNKGYAAKNSAYA which matches annotation for CEBPA: CCAAT/enhancer binding protein (C/EBP), alpha	14	0,07	9,00E-09	3,96E-07
ATATGCA,MIR-448	212	Targets of MicroRNA ATATGCA,MIR-448	14	0,066	1,89E-08	7,89E-07
CTATGCA,MIR-153	216	Targets of MicroRNA CTATGCA,MIR-153	14	0,0648	2,39E-08	9,51E-07
CAGCTG_V$AP4_Q5	1524	Genes with promoter regions [-2kb,2kb] around transcription start site containing the motif CAGCTG which matches annotation for REPIN1: replication initiator 1	39	0,0256	2,65E-08	1,01E-06
TGGTGCT,MIR-29A,MIR-29B,MIR-29C	521	Targets of MicroRNA TGGTGCT,MIR-29A,MIR-29B,MIR-29C	21	0,0403	3,80E-08	1,38E-06
GTGCCAA,MIR-96	303	Targets of MicroRNA GTGCCAA,MIR-96	16	0,0528	4,30E-08	1,49E-06
V$E2F_Q3	227	Genes with promoter regions [-2kb,2kb] around transcription start site containing motif TTTCGCGC. Motif does not match any known transcription factor	14	0,0617	4,45E-08	1,49E-06
ATGTACA,MIR-493	314	Targets of MicroRNA ATGTACA,MIR-493	16	0,051	7,02E-08	2,26E-06
CATTGTYY_V$SOX9_B1	358	Genes with promoter regions [-2kb,2kb] around transcription start site containing the motif CATTGTYY which matches annotation for SOX9: SRY (sex determining region Y)-box 9 (campomelic dysplasia, autosomal sex-reversal)	17	0,0475	7,58E-08	2,26E-06
CTTTAAR_UNKNOWN	972	Genes with promoter regions [-2kb,2kb] around transcription start site containing motif CTTTAAR. Motif does not match any known transcription factor	29	0,0298	7,58E-08	2,26E-06
RTAAACA_V$FREAC2_01	919	Genes with promoter regions [-2kb,2kb] around transcription start site containing the motif RTAAACA which matches annotation for FOXF2: forkhead box F2	28	0,0305	8,33E-08	2,40E-06
CACTGCC,MIR-34A,MIR-34C,MIR-449	280	Targets of MicroRNA CACTGCC,MIR-34A,MIR-34C,MIR-449	15	0,0536	9,42E-08	2,62E-06
V$RP58_01	207	Genes with promoter regions [-2kb,2kb] around transcription start site containing the motif NNAACATCTGGA which matches annotation for ZNF238: zinc finger protein 238	13	0,0628	1,09E-07	2,95E-06
RNGTGGGC_UNKNOWN	766	Genes with promoter regions [-2kb,2kb] around transcription start site containing motif RNGTGGGC. Motif does not match any known transcription factor	25	0,0326	1,16E-07	3,03E-06
TATAAA_V$TATA_01	1296	Genes with promoter regions [-2kb,2kb] around transcription start site containing the motif TATAAA which matches annotation for TAF<br>TATA	34	0,0262	1,21E-07	3,06E-06
V$TFIIA_Q6	251	Genes with promoter regions [-2kb,2kb] around transcription start site containing the motif TMTRWRAGGRSS which matches annotation for GTF2A1: general transcription factor IIA, 1, 19/37kDa GTF2A2: general transcription factor IIA, 2, 12kDa	14	0,0558	1,54E-07	3,79E-06
GCACTTT,MIR-17-5P,MIR-20A,MIR-106A,MIR-106B,MIR-20B,MIR-519D	595	Targets of MicroRNA GCACTTT,MIR-17-5P,MIR-20A,MIR-106A,MIR-106B,MIR-20B,MIR-519D	21	0,0353	3,43E-07	8,08E-06
CAGTGTT,MIR-141,MIR-200A	310	Targets of MicroRNA CAGTGTT,MIR-141,MIR-200A	15	0,0484	3,48E-07	8,08E-06
CTTTGCA,MIR-527	235	Targets of MicroRNA CTTTGCA,MIR-527	13	0,0553	4,69E-07	1,06E-05
TAGCTTT,MIR-9	236	Targets of MicroRNA TAGCTTT,MIR-9	13	0,0551	4,93E-07	1,08E-05
TGCCAAR_V$NF1_Q6	722	Genes with promoter regions [-2kb,2kb] around transcription start site containing the motif TGCCAAR which matches annotation for NF1: neurofibromin 1 (neurofibromatosis, von Recklinghausen disease, Watson disease)	23	0,0319	5,67E-07	1,22E-05
RYTTCCTG_V$ETS2_B	1085	Genes with promoter regions [-2kb,2kb] around transcription start site containing the motif RYTTCCTG which matches annotation for ETS2: v-ets erythroblastosis virus E26 oncogene homolog 2 (avian)	29	0,0267	7,24E-07	1,51E-05

AAGCCAT,MIR-135A,MIR-135B	335	Targets of MicroRNA AAGCCAT,MIR-135A,MIR-135B	15	0,0448	9,23E-07	1,88E-05
MGGAAGTG_V$GABP_B	757	Genes with promoter regions [-2kb,2kb] around transcription start site containing the motif MGGAAGTG which matches annotation for GABPA: GA binding protein transcription factor, alpha subunit 60kDa<br>GABPB2: GA binding protein transcription factor, beta subunit 2	23	0,0304	1,27E-06	2,52E-05
TACTTGA,MIR-26A,MIR-26B	300	Targets of MicroRNA TACTTGA,MIR-26A,MIR-26B	14	0,0467	1,31E-06	2,55E-05
TCTATGA,MIR-376A,MIR-376B	84	Targets of MicroRNA TCTATGA,MIR-376A,MIR-376B	8	0,0952	1,41E-06	2,67E-05
WGTTNNNNNAAA_UNKNOWN	547	Genes with promoter regions [-2kb,2kb] around transcription start site containing motif WGTTNNNNNAAA. Motif does not match any known transcription factor	19	0,0347	1,57E-06	2,92E-05
V$RORA2_01	151	Genes with promoter regions [-2kb,2kb] around transcription start site containing the motif NWAWNTAGGTCAN which matches annotation for RORA: RAR-related orphan receptor A	10	0,0662	1,99E-06	3,62E-05
V$VDR_Q6	269	Genes with promoter regions [-2kb,2kb] around transcription start site containing the motif CNSNNTGAACCN which matches annotation for VDR: vitamin D (1,25- dihydroxyvitamin D3) receptor	13	0,0483	2,13E-06	3,78E-05
YYCATTCAWW_UNKNOWN	191	Genes with promoter regions [-2kb,2kb] around transcription start site containing motif YYCATTCAWW. Motif does not match any known transcription factor	11	0,0576	2,42E-06	4,22E-05
V$AP1_C	275	Genes with promoter regions [-2kb,2kb] around transcription start site containing the motif NTGASTCAG which matches annotation for JUN: jun oncogene	13	0,0473	2,71E-06	4,62E-05
V$E2F1_Q6_01	238	Genes with promoter regions [-2kb,2kb] around transcription start site containing the motif NNTTCGCGCS which matches annotation for E2F1: E2F transcription factor 1	12	0,0504	3,39E-06	5,55E-05

Immunological gene sets

Gene Set Name	# Genes in Gene Set (K)	Description	# Genes in Overlap (k)	k/K	p-value	FDR q-value
GSE17721_12H_VS_24H_GARDIQUIMOD_BMDM_UP	200	Genes up-regulated in comparison of dendritic cells (DC) stimulated with Gardiquimod (TLR7 agonist) at 12 h versus those stimulated with Gardiquimod (TLR7 agonist) at 24 h.	19	0,095	8,86E-14	1,69E-10
GSE24634_TREG_VS_TCONV_POST_DAY10_IL4_CONVERSION_DN	200	Genes down-regulated in comparison of CD25+ regulatory T cell (Treg) treated with IL4 [GeneID=3565] at day 10 versus CD25- T cells treated with IL4 [GeneID=3565] at 10 h.	16	0,08	1,09E-10	1,04E-07
GSE10325_LUPUS_BCELL_VS_LUPUS_MYELOID_DN	200	Genes down-regulated in comparison of systemic lupus erythematosus B cells versus systemic lupus erythromatosus myeloid cells.	15	0,075	1,03E-09	3,27E-07
GSE13485_DAY3_VS_DAY21_YF17D_VACCINE_PBMC_UP	200	Genes up-regulated in comparison of unstimulated peripheral blood mononuclear cells (PBMC) 3 days after stimulation with YF17D vaccine versus PBMC 21 days after the stimulation.	15	0,075	1,03E-09	3,27E-07
GSE36476_CTRL_VS_TSST_ACT_40H_MEMORY_CD4_TCELL_OLD_DN	200	Genes down-regulated in comparison of untreated CD4 [GeneID=920] memory T cells from old donors versus those treated with TSST at 40 h.	15	0,075	1,03E-09	3,27E-07
GSE9988_LOW_LPS_VS_VEHICLE_TREATED_MONOCYTE_UP	200	Genes up-regulated in comparison of monocytes treated with 1 ng/ml LPS (TLR4 agonist) versus monocytes treated with control IgG.	15	0,075	1,03E-09	3,27E-07
GSE11057_NAIVE_VS_EFF_MEMORY_CD4_TCELL_UP	200	Genes up-regulated in comparison of naive T cells versus effector memory T cells.	14	0,07	9,00E-09	1,23E-06
GSE14769_UNSTIM_VS_60MIN_LPS_BMDM_DN	200	Genes down-regulated in comparison of unstimulated macrophage cells versus macrophage cells stimulated with LPS (TLR4 agonist) for 60 min.	14	0,07	9,00E-09	1,23E-06
GSE24142_EARLY_THYMIC_PROGENITOR_VS_DN3_THYMOCYTE_ADULT_UP	200	Genes up-regulated in comparison of adult thymic progenitors versus adult DN3 thymocytes.	14	0,07	9,00E-09	1,23E-06
GSE24634_TEFF_VS_TCONV_DAY7_IN_CULTURE_DN	200	Genes down-regulated in comparison of untreated CD25+ T effector cells at day 7 versus untreated CD25- T cells at day 7.	14	0,07	9,00E-09	1,23E-06
GSE29618_MONOCYTE_VS_MDC_DAY7_FLU_VACCINE_UP	200	Genes up-regulated in comparison of monocytes from influenza vaccinee at day 7 post-vaccination versus myeloid dendritic cells at day 7 post-vaccination.	14	0,07	9,00E-09	1,23E-06
GSE29618_MONOCYTE_VS_MDC_UP	200	Genes up-regulated in comparison of monocytes versus myeloid dendritic cells (mDC).	14	0,07	9,00E-09	1,23E-06
GSE29618_PDC_VS_MDC_UP	200	Genes up-regulated in comparison of plasmacytoid dendritic cells (DC) versus myeloid DCs.	14	0,07	9,00E-09	1,23E-06
GSE36476_CTRL_VS_TSST_ACT_40H_MEMORY_CD4_TCELL_YOUNG_DN	200	Genes down-regulated in comparison of untreated CD4 [GeneID=920] memory T cells from young donors versus those treated with TSST at 40 h.	14	0,07	9,00E-09	1,23E-06
GSE12366_GC_VS_MEMORY_BCELL_UP	200	Genes up-regulated in comparison of germinal center B cells versus memory B cells.	13	0,065	7,31E-08	4,66E-06
GSE12366_GC_VS_NAIVE_BCELL_UP	200	Genes up-regulated in comparison of germinal center B cells versus naive B cells.	13	0,065	7,31E-08	4,66E-06
GSE13411_IGM_MEMORY_BCELL_VS_PLASMA_CELL_DN	200	Genes down-regulated in comparison of IgM-memory B cells versus plasma cells.	13	0,065	7,31E-08	4,66E-06
GSE13411_SWITCHED_MEMORY_BCELL_VS_PLASMA_CELL_DN	200	Genes down-regulated in comparison of Ig isotype switched memory B cells versus plasma cells.	13	0,065	7,31E-08	4,66E-06
GSE1460_DP_THYMOCYTE_VS_THYMIC_STROMAL_CELL_DN	200	Genes down-regulated in comparison of CD4 [GeneID=920] CD8 thymocytes versus thymic stromal cells.	13	0,065	7,31E-08	4,66E-06
GSE14769_UNSTIM_VS_80MIN_LPS_BMDM_DN	200	Genes down-regulated in comparison of unstimulated macrophage cells versus macrophage cells stimulated with LPS (TLR4 agonist) for 80 min.	13	0,065	7,31E-08	4,66E-06
GSE15930_STIM_VS_STIM_AND_TRICHOSTATINA_48H_CD8_T_CELL_UP	200	Genes up-regulated in comparison of unstimulated CD8 T cells at 48 h versus CD8 T cells at 48 h after treatment with trichostatin A (TSA) [PubChem=5562].	13	0,065	7,31E-08	4,66E-06
GSE17721_LPS_VS_PAM3CSK4_1H_BMDM_DN	200	Genes down-regulated in comparison of dendritic cells (DC) stimulated with LPS (TLR4 agonist) at 1 h versus DC cells stimulated with Pam3Csk4 (TLR1/2 agonist) at 1 h.	13	0,065	7,31E-08	4,66E-06
GSE2706_UNSTIM_VS_2H_LPS_AND_R848_DC_DN	200	Genes down-regulated in comparison of unstimulated dendritic cells (DC) at 0 h versus DCs stimulated with LPS (TLR4 agonist) and R848 for 2 h.	13	0,065	7,31E-08	4,66E-06
GSE29618_LAIV_VS_TIV_FLU_VACCINE_DAY7_MDC_UP	200	Genes up-regulated in comparison of myeloid dendritic cells from LAIV influenza vaccinee at day 7 post-vaccination vesus those from TIV influenza vaccinee at day 7.	13	0,065	7,31E-08	4,66E-06
GSE36476_CTRL_VS_TSST_ACT_72H_MEMORY_CD4_TCELL_OLD_DN	200	Genes down-regulated in comparison of untreated CD4 [GeneID=920] memory T cells from old donors versus those treated with TSST at 72 h.	13	0,065	7,31E-08	4,66E-06
GSE36476_CTRL_VS_TSST_ACT_72H_MEMORY_CD4_TCELL_YOUNG_DN	200	Genes down-regulated in comparison of untreated CD4 [GeneID=920] memory T cells from young donors versus those treated with TSST at 72 h.	13	0,065	7,31E-08	4,66E-06
GSE7852_LN_VS_FAT_TCONV_DN	200	Genes down-regulated in comparison of lymph node conventional T cells versus fat tissue conventional T cells.	13	0,065	7,31E-08	4,66E-06
GSE7852_LN_VS_THYMUS_TCONV_UP	200	Genes up-regulated in comparison of lymph node conventional T cells versus thymus conventional T cells.	13	0,065	7,31E-08	4,66E-06
GSE9988_LPS_VS_LPS_AND_ANTI_TREM1_MONOCYTE_DN	200	Genes down-regulated in comparison of monocytes treated with 5000 ng/ml LPS (TLR4 agonist) versus monocytes treated with anti-TREM1 [GeneID=54210].	13	0,065	7,31E-08	4,66E-06
GSE9988_LPS_VS_VEHICLE_TREATED_MONOCYTE_UP	200	Genes up-regulated in comparison of monocytes treated with 1 ng/ml LPS (TLR4 agonist) versus monocytes treated with vehicle.	13	0,065	7,31E-08	4,66E-06
GSE30962_ACUTE_VS_CHRONIC_LCMV_PRIMARY_INF_CD8_TCELL_DN	199	Genes down-regulated in comparison of splenic primary CD8 effector T cells at day 8 post-acute infection versus splenic primary CD8 effector T cells at day 8 post-chronic infection.	12	0,0603	5,20E-07	1,69E-05
GSE10325_LUPUS_CD4_TCELL_VS_LUPUS_MYELOID_DN	200	Genes down-regulated in comparison of systemic lupus erythematosus CD4 [GeneID=920] T cells versus systemic lupus erythematosus myeloid cells.	12	0,06	5,49E-07	1,69E-05
GSE13484_UNSTIM_VS_12H_YF17D_VACCINE_STIM_PBMC_DN	200	Genes down-regulated in comparison of unstimulated peripheral blood mononuclear cells (PBMC) cultured for 0 h versus PBMC cultured for 12 h with YF17D vaccine.	12	0,06	5,49E-07	1,69E-05
GSE14769_UNSTIM_VS_120MIN_LPS_BMDM_DN	200	Genes down-regulated in comparison of unstimulated macrophage cells versus macrophage cells stimulated with LPS (TLR4 agonist) for 120 min.	12	0,06	5,49E-07	1,69E-05
GSE15750_DAY6_VS_DAY10_EFF_CD8_TCELL_UP	200	Genes up-regulated in comparison of wild type CD8 effector T cells at day 6 versus those at day 10.	12	0,06	5,49E-07	1,69E-05
GSE17721_ALL_VS_24H_PAM3CSK4_BMDM_UP	200	Genes up-regulated in comparison of dendritic cells (DC) stimulated with Pam3Csk4 (TLR1/2 agonist) at all time points versus those stimulated with Pam3Csk4 (TLR1/2 agonist) at 24 h only.	12	0,06	5,49E-07	1,69E-05
GSE17721_LPS_VS_CPG_6H_BMDM_UP	200	Genes up-regulated in comparison of dendritic cells (DC) stimulated with LPS (TLR4 agonist) at 6 h versus DC cells stimulated with CpG DNA (TLR9 agonist) at 6 h.	12	0,06	5,49E-07	1,69E-05
GSE17721_PAM3CSK4_VS_GADIQUIMOD_6H_BMDM_DN	200	Genes down-regulated in comparison of dendritic cells (DC) stimulated with Pam3Csk4 (TLR1/2 agonist) at 6 h versus DC cells stimulated with Gardiquimod (TLR7 agonist) at 6 h.	12	0,06	5,49E-07	1,69E-05
GSE17974_IL4_AND_ANTI_IL12_VS_UNTREATED_72H_ACT_CD4_TCELL_DN	200	Genes down-regulated in comparison of CD4 [GeneID=920] T cells treated with IL4 [GeneID=3565] and anti-IL12 at 72 h versus the untreated cells at 72 h.	12	0,06	5,49E-07	1,69E-05
GSE20366_EX_VIVO_VS_HOMEOSTATIC_CONVERSION_TREG_DN	200	Genes down-regulated in comparison of TregLP versus Homeo Convert (see Table 1S in the paper for details).	12	0,06	5,49E-07	1,69E-05
GSE20715_WT_VS_TLR4_KO_48H_OZONE_LUNG_UP	200	Genes up-regulated in comparison of lung tissue from wild type mice subjected to ozone for 48 h versus that from TLR4 [GeneID=7099] deficient mice subjected to ozone for 48 h.	12	0,06	5,49E-07	1,69E-05
GSE22886_UNSTIM_VS_IL15_STIM_NKCELL_DN	200	Genes down-regulated in comparison of unstimulated NK cells versus those stimulated with IL2 [GeneID=3600] at 16 h.	12	0,06	5,49E-07	1,69E-05
GSE22886_UNSTIM_VS_IL2_STIM_NKCELL_DN	200	Genes down-regulated in comparison of unstimulated NK cells versus those stimulated with IL2 [GeneID=3558] at 16 h.	12	0,06	5,49E-07	1,69E-05
GSE24142_ADULT_VS_FETAL_DN2_THYMOCYTE_UP	200	Genes up-regulated in comparison of adult DN2 thymocytes versus fetal DN2 thymocytes.	12	0,06	5,49E-07	1,69E-05
GSE24142_EARLY_THYMIC_PROGENITOR_VS_DN2_THYMOCYTE_ADULT_UP	200	Genes up-regulated in comparison of adult thymic progenitors versus adult DN2 thymocytes.	12	0,06	5,49E-07	1,69E-05
GSE24142_EARLY_THYMIC_PROGENITOR_VS_DN2_THYMOCYTE_UP	200	Genes up-regulated in comparison of thymic progenitors versus DN2 thymocytes.	12	0,06	5,49E-07	1,69E-05
GSE24634_IL4_VS_CTRL_TREATED_NAIVE_CD4_TCELL_DAY10_DN	200	Genes down-regulated in comparison of CD25- T cells treated with IL4 [GeneID=3565] at day 10 versus untreated CD25- T cells at day 10.	12	0,06	5,49E-07	1,69E-05
GSE26495_NAIVE_VS_PD1LOW_CD8_TCELL_DN	200	Genes down-regulated in comparison of naive CD8 T cells versus PD-1 low CD8 T cells.	12	0,06	5,49E-07	1,69E-05
GSE26928_NAIVE_VS_CENT_MEMORY_CD4_TCELL_DN	200	Genes down-regulated in comparison of naive CD4 [GeneID=920] T cells versus CD4 [GeneID=920] central memory T cells.	12	0,06	5,49E-07	1,69E-05
GSE30083_SP3_VS_SP4_THYMOCYTE_DN	200	Genes down-regulated in comparison of SP3 thymocytes versus SP4 thymocytes.	12	0,06	5,49E-07	1,69E-05

Oncogenic gene sets

Gene Set Name	# Genes in Gene Set (K)	Description	# Genes in Overlap (k)	k/K	p-value	FDR q-value
RB_P107_DN.V1_UP	140	Genes up-regulated in primary keratinocytes from RB1 and RBL1 [Gene ID=5925, 5933] skin specific knockout mice.	14	0,1	8,30E-11	1,57E-08
E2F1_UP.V1_DN	193	Genes down-regulated in mouse fibroblasts over-expressing E2F1 [Gene ID=1869] gene.	14	0,0725	5,71E-09	5,39E-07
MEL18_DN.V1_UP	141	Genes up-regulated in DAOY cells (medulloblastoma) upon knockdown of PCGF2 [Gene ID=7703] gene by RNAi.	12	0,0851	1,18E-08	7,44E-07
LEF1_UP.V1_UP	195	Genes up-regulated in DLD1 cells (colon carcinoma) over-expressing LEF1 [Gene ID=51176].	13	0,0667	5,44E-08	2,05E-06
PRC2_EZH2_UP.V1_UP	195	Genes up-regulated in TIG3 cells (fibroblasts) upon knockdown of EZH2 [Gene ID=2146] gene.	13	0,0667	5,44E-08	2,05E-06
BMI1_DN_MEL18_DN.V1_UP	145	Genes up-regulated in DAOY cells (medulloblastoma) upon knockdown of BMI1 and PCGF2 [Gene ID=648, 7703] genes by RNAi.	11	0,0759	1,57E-07	4,93E-06
EGFR_UP.V1_UP	193	Genes up-regulated in MCF-7 cells (breast cancer) positive for ESR1 [Gene ID=2099] and engineered to express ligand-activatable EGFR [Gene ID=1956].	12	0,0622	3,75E-07	1,01E-05
STK33_SKM_UP	290	Genes up-regulated in SKM-1 cells (AML) after knockdown of STK33 [Gene ID=65975] by RNAi.	14	0,0483	8,78E-07	2,07E-05
BMI1_DN.V1_UP	147	Genes up-regulated in DAOY cells (medulloblastoma) upon knockdown of BMI1 [Gene ID=648] gene by RNAi.	10	0,068	1,56E-06	3,02E-05
ESC_J1_UP_EARLY.V1_UP	183	Genes up-regulated during early stages of differentiation of embryoid bodies from J1 embryonic stem cells.	11	0,0601	1,60E-06	3,02E-05
LTE2_UP.V1_UP	190	Genes up-regulated in MCF-7 cells (breast cancer) positive for ESR1 [Gene ID=2099] MCF-7 cells (breast cancer) and long-term adapted for estrogen-independent growth.	11	0,0579	2,30E-06	3,95E-05
RPS14_DN.V1_UP	192	Genes up-regulated in CD34+ hematopoietic progenitor cells after knockdown of RPS14 [Gene ID=6208] by RNAi.	11	0,0573	2,55E-06	4,01E-05
P53_DN.V1_UP	194	Genes up-regulated in NCI-60 panel of cell lines with mutated TP53 [Gene ID=7157].	11	0,0567	2,82E-06	4,09E-05
TBK1.DF_UP	290	Genes up-regulated in epithelial lung cancer cell lines upon over-expression of an oncogenic form of KRAS [Gene ID=3845] gene and knockdown of TBK1 [Gene ID=29110] gene by RNAi.	13	0,0448	4,83E-06	6,52E-05

ERB2_UP.V1_UP	191	Genes up-regulated in MCF-7 cells (breast cancer) positive for ESR1 [Gene ID=2099] and engineered to express ligand-activatable ERBB2 [Gene ID=2064].	10	0,0524	1,59E-05	1,83E-04
ATF2_UP.V1_UP	192	Genes up-regulated in myometrial cells over-expressing ATF2 [Gene ID=1386] gene.	10	0,0521	1,67E-05	1,83E-04
P53_DN.V1_DN	192	Genes down-regulated in NCI-60 panel of cell lines with mutated TP53 [Gene ID=7157].	10	0,0521	1,67E-05	1,83E-04
ATF2_S_UP.V1_UP	193	Genes up-regulated in myometrial cells over-expressing a shortened splice form of ATF2 [Gene ID=1386] gene.	10	0,0518	1,75E-05	1,83E-04
HOXA9_DN.V1_DN	195	Genes down-regulated in MOLM-14 cells (AML) with knockdown of HOXA9 [Gene ID=3205] gene by RNAi vs controls.	10	0,0513	1,91E-05	1,88E-04
MEK_UP.V1_UP	196	Genes up-regulated in MCF-7 cells (breast cancer) positive for ESR1 [Gene ID=2099] MCF-7 cells (breast cancer) stably over-expressing constitutively active MAP2K1 [Gene ID=5604] gene.	10	0,051	1,99E-05	1,88E-04
STK33_NOMO_UP	294	Genes up-regulated in NOMO-1 cells (AML) after knockdown of STK33 [Gene ID=65975] by RNAi.	12	0,0408	2,81E-05	2,53E-04
PKCA_DN.V1_DN	167	Genes down-regulated in small intenstine in PRKCA [Gene ID=5578] knockout mice.	9	0,0539	3,37E-05	2,89E-04
ESC_V6.5_UP_EARLY.V1_DN	172	Genes down-regulated during early stages of differentiation of embryoid bodies from V6.5 embryonic stem cells.	9	0,0523	4,24E-05	3,48E-04
NFE2L2.V2	481	Genes up-regulated in MEF cells (embryonic fibroblasts) with knockout of NFE2L2 [Gene ID=4780] gene.	15	0,0312	6,65E-05	5,08E-04
PDGF_UP.V1_DN	142	Genes down-regulated in SH-SY5Y cells (neuroblastoma) in response to PDGF [Gene ID=] stimulation.	8	0,0563	6,72E-05	5,08E-04
ATF2_UP.V1_DN	187	Genes down-regulated in myometrial cells over-expressing ATF2 [Gene ID=1386] gene.	9	0,0481	8,09E-05	5,71E-04
PDGF_UP.V1_UP	146	Genes up-regulated in SH-SY5Y cells (neuroblastoma) in response to PDGF [Gene ID=] stimulation.	8	0,0548	8,16E-05	5,71E-04
BCAT_GDS748_UP	48	Genes up-regulated in HEK293 cells (kidney fibroblasts) expressing constitutively active form of CTNNB1 [Gene ID=1499] gene.	5	0,1042	9,10E-05	6,14E-04
JNK_DN.V1_DN	191	Genes down-regulated in JNK inhibitor-treated (SP600125[PubChem=8515]) keratinocytes.	9	0,0471	9,51E-05	6,19E-04
IL2_UP.V1_UP	192	Genes up-regulated in Sez-4 cells (T lymphocyte) that were first starved of IL2 [Gene ID=3558] and then stimulated with IL2 [Gene ID=3558].	9	0,0469	9,89E-05	6,23E-04
PRC2_EDD_UP.V1_UP	193	Genes up-regulated in TIG3 cells (fibroblasts) upon knockdown of EED [Gene ID=8726] gene.	9	0,0466	1,03E-04	6,27E-04
PRC2_EZH2_UP.V1_DN	194	Genes down-regulated in TIG3 cells (fibroblasts) upon knockdown of EZH2 [Gene ID=2146] gene.	9	0,0464	1,07E-04	6,32E-04
EGFR_UP.V1_DN	196	Genes down-regulated in MCF-7 cells (breast cancer) positive for ESR1 [Gene ID=2099] and engineered to express ligand-activatable EGFR [Gene ID=1956].	9	0,0459	1,16E-04	6,43E-04
LTE2_UP.V1_DN	196	Genes down-regulated in MCF-7 cells (breast cancer) positive for ESR1 [Gene ID=2099] MCF-7 cells (breast cancer) and long-term adapted for estrogen-independent growth.	9	0,0459	1,16E-04	6,43E-04
CSR_LATE_UP.V1_UP	172	Genes up-regulated in late serum response of CRL 2091 cells (foreskin fibroblasts).	8	0,0465	2,52E-04	1,36E-03
NRL_DN.V1_UP	136	Genes up-regulated in retina cells from NRL [Gene ID=4901] knockout mice.	7	0,0515	3,32E-04	1,74E-03
RB_DN.V1_UP	137	Genes up-regulated in primary keratinocytes from RB1 [Gene ID=5925] skin specific knockout mice.	7	0,0511	3,47E-04	1,77E-03
GCNP_SHH_UP_LATE.V1_UP	183	Genes up-regulated in granule cell neuron precursors (GCNPs) after stimulation with Shh for 24h.	8	0,0437	3,83E-04	1,80E-03
RAPA_EARLY_UP.V1_UP	183	Genes up-regulated in BJAB (lymphoma) cells by everolimus [PubChem = 6442177].	8	0,0437	3,83E-04	1,80E-03
CAHOY_ASTROGLIAL	100	Genes up-regulated in astrogia cells.	6	0,06	3,91E-04	1,80E-03
EIF4E_DN	100	Genes down-regulated in HMEC cells (primary mammary epithelium) upon over-expression of EIF4E [Gene ID=1977] gene.	6	0,06	3,91E-04	1,80E-03
TBK1.DF_DN	287	Genes down-regulated in epithelial lung cancer cell lines upon over-expression of an oncogenic form of KRAS [Gene ID=3845] gene and knockdown of TBK1 [Gene ID=29110] gene by RNAi.	10	0,0348	4,56E-04	2,05E-03
KRAS.KIDNEY_UP.V1_UP	145	Genes up-regulated in epithelial kidney cancer cell lines over-expressing an oncogenic form of KRAS [Gene ID=3845] gene.	7	0,0483	4,89E-04	2,15E-03
STK33_NOMO_DN	292	Genes down-regulated in NOMO-1 cells (AML) after knockdown of STK33 [Gene ID=65975] by RNAi.	10	0,0342	5,22E-04	2,16E-03
IL15_UP.V1_UP	192	Genes up-regulated in Sez-4 cells (T lymphocyte) that were first starved of IL2 [Gene ID=3558] and then stimulated with IL15 [Gene ID=3600].	8	0,0417	5,26E-04	2,16E-03
TGFB_UP.V1_UP	192	Genes up-regulated in a panel of epithelial cell lines by TGFB1 [Gene ID=7040].	8	0,0417	5,26E-04	2,16E-03
CYCLIN_D1_KE_.V1_DN	194	Genes down-regulated in MCF-7 cells (breast cancer) over-expressing a mutant K112E form of CCND1 [Gene ID=595] gene.	8	0,0412	5,63E-04	2,22E-03
HOXA9_DN.V1_UP	194	Genes up-regulated in MOLM-14 cells (AML) with knockdown of HOXA9 [Gene ID=3205] gene by RNAi vs controls.	8	0,0412	5,63E-04	2,22E-03
RELA_DN.V1_UP	149	Genes up-regulated in HEK293 cells (kidney fibroblasts) upon knockdown of RELA [Gene ID=5970] gene by RNAi.	7	0,047	5,75E-04	2,22E-03
E2F3_UP.V1_UP	196	Genes up-regulated in primary epithelial breast cancer cell culture over-expressing E2F3 [Gene ID=1871] gene.	8	0,0408	6,02E-04	2,28E-03

GO gene sets						
Gene Set Name	# Genes in Gene Set (K)	GO term	# Genes in Overlap (k)	k/K	p-value	FDR q-value
MEMBRANE	1994	GO:0016020	62	0,0311	2,29E-16	3,32E-13
MEMBRANE_PART	1670	GO:0044425	53	0,0317	1,91E-14	1,39E-11
BIOPOLYMER_METABOLIC_PROCESS	1684	GO:0043283	50	0,0297	1,31E-12	6,09E-10
NUCLEOBASENUCLEOSIDENUCLEOTIDE_AND_NUCLEIC_ACID_METABOLIC_PROCESS	1244	GO:0006139	42	0,0338	1,67E-12	6,09E-10
INTEGRAL_TO_MEMBRANE	1330	GO:0016021	42	0,0316	1,39E-11	4,05E-09
INTRINSIC_TO_MEMBRANE	1348	GO:0031224	42	0,0312	2,12E-11	5,13E-09
CYTOPLASM	2131	GO:0005737	54	0,0253	6,29E-11	1,31E-08
TRANSCRIPTION_DNA_DEPENDENT	636	GO:0006351	26	0,0409	6,68E-10	1,04E-07
MULTICELLULAR_ORGANISMAL_DEVELOPMENT	1049	GO:0007275	34	0,0324	6,93E-10	1,04E-07
RNA_BIOSYNTHETIC_PROCESS	638	GO:0032774	26	0,0408	7,13E-10	1,04E-07
TRANSCRIPTION	753	GO:0006350	28	0,0372	1,20E-09	1,59E-07
PLASMA_MEMBRANE	1426	GO:0005886	40	0,0281	1,37E-09	1,66E-07
RNA_METABOLIC_PROCESS	841	GO:0016070	29	0,0345	3,31E-09	3,70E-07
ANATOMICAL_STRUCTURE_DEVELOPMENT	1013	GO:0048856	32	0,0316	4,13E-09	4,27E-07
SKELETAL_DEVELOPMENT	103	GO:0001501	11	0,1068	4,40E-09	4,27E-07
SYSTEM_DEVELOPMENT	861	GO:0048731	29	0,0337	5,57E-09	5,06E-07
TRANSCRIPTION_FROM_RNA_POLYMERASE_II_PROMOTER	457	GO:0006366	20	0,0438	2,10E-08	1,79E-06
REGULATION_OF_CELLULAR_METABOLIC_PROCESS	787	GO:0031323	26	0,033	5,09E-08	4,11E-06
REGULATION_OF_METABOLIC_PROCESS	799	GO:0019222	26	0,0325	6,84E-08	5,24E-06
CELL_PROJECTION	108	GO:0042995	10	0,0926	8,83E-08	6,42E-06
REGULATION_OF_NUCLEOBASENUCLEOSIDENUCLEOTIDE_AND_NUCLEIC_ACID_METABOLIC_PROCESS	618	GO:0019219	22	0,0356	1,56E-07	1,08E-05
ORGAN_DEVELOPMENT	571	GO:0048513	21	0,0368	1,75E-07	1,16E-05
NUCLEUS	1430	GO:0005634	35	0,0245	4,00E-07	2,53E-05
REGULATION_OF_TRANSCRIPTIONDNA_DEPENDENT	461	GO:0006355	18	0,039	5,65E-07	3,43E-05
REGULATION_OF_TRANSCRIPTION	566	GO:0045449	20	0,0353	6,41E-07	3,73E-05
REGULATION_OF_RNA_METABOLIC_PROCESS	471	GO:0051252	18	0,0382	7,69E-07	4,30E-05
PLASMA_MEMBRANE_PART	1158	GO:0044459	30	0,0259	8,82E-07	4,64E-05
BASOLATERAL_PLASMA_MEMBRANE	35	GO:0016323	6	0,1714	8,94E-07	4,64E-05
ACTIN_POLYMERIZATION_AND_OR_DEPOLYMERIZATION	23	GO:0008154	5	0,2174	2,17E-06	1,09E-04
SIGNAL_TRANSDUCTION	1634	GO:0007165	36	0,022	3,05E-06	1,48E-04
IMMUNE_SYSTEM_PROCESS	332	GO:0002376	14	0,0422	4,25E-06	1,99E-04
NEGATIVE_REGULATION_OF_CELLULAR_PROCESS	646	GO:0048523	20	0,031	4,73E-06	2,15E-04
CYTOSKELETON_ORGANIZATION_AND_BIOGENESIS	208	GO:0007010	11	0,0529	5,49E-06	2,42E-04
BONE_REMODELING	29	GO:0046849	5	0,1724	7,31E-06	3,08E-04
DNA_METABOLIC_PROCESS	257	GO:0006259	12	0,0467	7,40E-06	3,08E-04
PROTEIN_METABOLIC_PROCESS	1231	GO:0019538	29	0,0236	8,33E-06	3,26E-04
REGULATION_OF_GENE_EXPRESSION	673	GO:0010468	20	0,0297	8,60E-06	3,26E-04
TISSUE_REMODELING	30	GO:0048771	5	0,1667	8,71E-06	3,26E-04
AMINE_METABOLIC_PROCESS	141	GO:0009308	9	0,0638	8,74E-06	3,26E-04
NEGATIVE_REGULATION_OF_BIOLOGICAL_PROCESS	677	GO:0048519	20	0,0295	9,37E-06	3,40E-04
CYTOPLASMIC_PART	1383	GO:0044444	31	0,0224	1,08E-05	3,83E-04
TRANSFERASE_ACTIVITY_TRANSFERRING_PHOSPHORUS_CONTAINING_GROUPS	424	GO:0016772	15	0,0354	1,58E-05	5,48E-04
NITROGEN_COMPOUND_METABOLIC_PROCESS	155	GO:0006807	9	0,0581	1,87E-05	6,32E-04

DNA_BINDING	602	GO:0003677	18	0,0299	2,22E-05	7,35E-04
REGULATION_OF_TRANSCRIPTION_FROM_RNA_POLYMERASE_II_PROMOTER	289	GO:0006357	12	0,0415	2,38E-05	7,69E-04
POSITIVE_REGULATION_OF_CELLULAR_PROCESS	668	GO:0048522	19	0,0284	2,62E-05	8,27E-04
RESPONSE_TO_STRESS	508	GO:0006950	16	0,0315	3,41E-05	1,05E-03
CELLULAR_MACROMOLECULE_METABOLIC_PROCESS	1131	GO:0044260	26	0,023	3,67E-05	1,11E-03
NEGATIVE_REGULATION_OF_NUCLEOBASENUCLEOSIDENUCLEOTIDE_AND_NUCLEIC_ACID_METABOLIC_PROCESS	211	GO:0045934	10	0,0474	3,74E-05	1,11E-03
TRANSCRIPTION_FACTOR_ACTIVITY	354	GO:0003700	13	0,0367	3,94E-05	1,15E-03

Supplementary Table S1c. VERHAAK_GLIOBLASTOMA_OVERLAP

	Overlap_Mesenchymal	Overlap_Classical	Overlap_Neural	Overlap_Proneural
p value	1,47E-15	1,26E-07	0,5913129	2,18E-10
Adjusted p value	6,95E-12	5,94E-04	1	1,03E-06
	BNC2	ABCD2	EDIL3	ABAT
	CASP4	B3GALT1	YPEL5	C1ORF61
	CEBPB	BLM		CDC25A
	COL1A2	ELOVL2		CHD7
	COL5A1	FZD3		CSPG5
	COPZ2	KLHL25		DPF1
	DAB2	KLHL4		FXYD6
	FXYD5	MEIS1		GNG4
	IL1R1	SLC4A4		MAST1
	ITGA5	WSCD1		MCM10
	MVP	ZNF134		NCALD
	MYH9	ZNF20		NLGN3
	P4HA2	ZNF419		OLIG2
	PLAUR			SEZ6L
	RBMS1			ZNF711
	RRAS			
	RUNX2			
	S100A4			
	SCPEP1			
	SEC24D			
	SLC10A3			

Supplementary Table S1d. GSEA GO enrichment

	Top 1000 Cluster 1 vs Cluster 2				Top 1000 GSCs and Schulte				
Gene Set Name	# Genes in Overlap (k)	k/K	p-value	FDR q-value	# Genes in Overlap (k)	k/K	p-value	FDR q-value	GO term
NUCLEUS	69	0,0483	5,15E-10	1,17E-08					GO:0005634
NUCLEOBASENUCLEOSIDENUCLEOTIDE_AND_NUCLEIC_ACID_METABOLIC_PROCESS	68	0,0547	3,28E-12	1,11E-10					GO:0006139
TRANSCRIPTION	48	0,0637	3,41E-11	8,86E-10					GO:0006350
INTRACELLULAR_SIGNALING_CASCADE	44	0,066	7,63E-11	1,88E-09					GO:0007242
REGULATION_OF_GENE_EXPRESSION	42	0,0624	1,09E-09	2,30E-08					GO:0010468
REGULATION_OF_NUCLEOBASENUCLEOSIDENUCLEOTIDE_AND_NUCLEIC_ACID_METABOLIC_PROCESS	41	0,0663	2,85E-10	6,69E-09					GO:0019219
INTRACELLULAR_NON_MEMBRANE_BOUND_ORGANELLE	40	0,0634	1,74E-09	3,47E-08					GO:0043232
NON_MEMBRANE_BOUND_ORGANELLE	40	0,0634	1,74E-09	3,47E-08					GO:0043228
REGULATION_OF_TRANSCRIPTION	38	0,0671	9,17E-10	1,96E-08					GO:0045449
DNA_BINDING	36	0,0598	4,90E-08	7,58E-07					GO:0003677
CELL_CYCLE_GO_0007049	32	0,1016	5,13E-13	2,07E-11					GO:0007049
POST_TRANSLATIONAL_PROTEIN_MODIFICATION	32	0,0672	1,87E-08	3,16E-07					GO:0043687
CYTOSKELETON	31	0,0845	1,35E-10	3,27E-09					GO:0005856
KINASE_ACTIVITY	31	0,084	1,54E-10	3,68E-09					GO:0016301
RECEPTOR_BINDING	29	0,0769	4,50E-09	8,72E-08					GO:0005102
PROTEIN_KINASE_ACTIVITY	28	0,0982	3,20E-11	8,47E-10					GO:0004672
RESPONSE_TO_CHEMICAL_STIMULUS	27	0,086	1,40E-09	2,88E-08					GO:0042221
PROTEIN_KINASE_CASCADE	25	0,0853	6,61E-09	1,22E-07					GO:0007243
TRANSCRIPTION_FACTOR_BINDING	24	0,0782	6,99E-08	1,02E-06					GO:0008134
CELL_CYCLE_PROCESS	23	0,1192	3,82E-11	9,74E-10					GO:0022402
MITOTIC_CELL_CYCLE	22	0,1438	2,32E-12	8,02E-11					GO:0000278
CELL_CYCLE_PHASE	22	0,1294	1,97E-11	5,51E-10					GO:0022403
POSITIVE_REGULATION_OF_CELL_PROLIFERATION	21	0,1409	1,08E-11	3,27E-10					GO:0008284
NEUROGENESIS	17	0,1828	1,42E-11	4,20E-10					GO:0022008
NITROGEN_COMPOUND_METABOLIC_PROCESS	17	0,1097	4,62E-08	7,22E-07					GO:0006807
NEURON_DIFFERENTIATION	14	0,1842	8,01E-10	1,76E-08					GO:0030182
NEURON_DEVELOPMENT	12	0,1967	5,91E-09	1,11E-07					GO:0048666
TRANSMEMBRANE_RECEPTOR_PROTEIN_KINASE_ACTIVITY	11	0,2157	9,13E-09	1,62E-07					GO:0019199
AXONOGENESIS	10	0,2326	1,99E-08	3,33E-07					GO:0007409
COLLAGEN	8	0,3478	1,61E-08	2,75E-07					GO:0005581
PHOSPHORIC_ESTER_HYDROLASE_ACTIVITY					19	0,1242	2,62E-10	9,07E-09	GO:0042578
SUBSTRATE_SPECIFIC_TRANSMEMBRANE_TRANSPORTER_ACTIVITY					28	0,0814	4,40E-10	1,45E-08	GO:0022891
SUBSTRATE_SPECIFIC_TRANSPORTER_ACTIVITY					30	0,0765	4,65E-10	1,47E-08	GO:0022892
TRANSMEMBRANE_TRANSPORTER_ACTIVITY					29	0,0773	7,11E-10	2,15E-08	GO:0022857
MEMBRANE_FRACTION					27	0,0796	1,44E-09	4,20E-08	GO:0005624
INTRACELLULAR_SIGNALING_CASCADE					39	0,0585	3,13E-09	8,94E-08	GO:0007242
RESPONSE_TO_WOUNDING					19	0,1	1,02E-08	2,75E-07	GO:0009611
IONOTROPIC_GLUTAMATE_RECEPTOR_ACTIVITY					6	0,6	1,19E-08	3,14E-07	GO:0004970
REGULATION_OF_SIGNAL_TRANSDUCTION					20	0,0901	2,49E-08	6,35E-07	GO:0009966
REGULATION_OF_PHOSPHORYLATION					10	0,2041	3,72E-08	9,17E-07	GO:0042325
ION_BINDING					22	0,0806	3,81E-08	9,24E-07	GO:0043167
HOMEOSTATIC_PROCESS					19	0,0918	4,11E-08	9,79E-07	GO:0042592
CELL_SURFACE					12	0,1519	5,32E-08	1,25E-06	GO:0009986
LIPID_RAFT					8	0,2759	6,98E-08	1,61E-06	GO:0045121
TRANSPORT					40	0,0503	1,18E-07	2,59E-06	GO:0006810
EXTRACELLULAR_STRUCTURE_ORGANIZATION_AND_BIOGENESIS					8	0,25	1,62E-07	3,42E-06	GO:0043062
ENZYME_ACTIVATOR_ACTIVITY					14	0,112	2,16E-07	4,42E-06	GO:0008047
POSITIVE_REGULATION_OF_CATALYTIC_ACTIVITY					16	0,097	2,24E-07	4,47E-06	GO:0043085
SYNAPSE_ORGANIZATION_AND_BIOGENESIS					7	0,3043	2,22E-07	4,47E-06	GO:0050808
BIOSYNTHETIC_PROCESS					28	0,0596	3,53E-07	6,76E-06	GO:0009058
ESTABLISHMENT_OF_LOCALIZATION					41	0,0471	4,55E-07	8,27E-06	GO:0051234
CELLULAR_MORPHOGENESIS_DURING_DIFFERENTIATION					9	0,1837	4,67E-07	8,39E-06	GO:0000904
CHEMICAL_HOMEOSTASIS					15	0,0968	5,46E-07	9,46E-06	GO:0048878
GLUTAMATE_SIGNALING_PATHWAY					6	0,3529	6,21E-07	1,06E-05	GO:0007215
REGULATION_OF_TRANSFERASE_ACTIVITY					15	0,0932	8,89E-07	1,42E-05	GO:0051338

	Top 1000 Cluster 1 vs Cluster 2				Top 1000 GSCs and Schulte				
**Gene Set Name**	**# Genes in Overlap (k)**	**k/K**	**p-value**	**FDR q-value**	**# Genes in Overlap (k)**	**k/K**	**p-value**	**FDR q-value**	**GO term**
ION_TRANSMEMBRANE_TRANSPORTER_ACTIVITY					20	0,0719	9,51E-07	1,49E-05	GO:0015075
POSITIVE_REGULATION_OF_TRANSFERASE_ACTIVITY					11	0,1279	1,13E-06	1,75E-05	GO:0051347
GTPASE_REGULATOR_ACTIVITY					13	0,104	1,37E-06	2,05E-05	GO:0030695
CELLULAR_HOMEOSTASIS					14	0,0952	1,57E-06	2,27E-05	GO:0019725
METAL_ION_TRANSMEMBRANE_TRANSPORTER_ACTIVITY					14	0,0952	1,57E-06	2,27E-05	GO:0046873
MEMBRANE	171	0,0858	3,20E-55	4,66E-52	172	0,0863	5,90E-61	8,58E-58	GO:0016020
PLASMA_MEMBRANE	139	0,0975	4,58E-51	3,33E-48	142	0,0996	1,49E-57	1,08E-54	GO:0005886
MEMBRANE_PART	139	0,0832	4,25E-43	1,03E-40	143	0,0856	6,69E-50	3,24E-47	GO:0044425
SIGNAL_TRANSDUCTION	129	0,0789	1,46E-37	2,35E-35	109	0,0667	2,13E-28	2,58E-26	GO:0007165
INTRINSIC_TO_MEMBRANE	123	0,0912	4,12E-42	8,55E-40	124	0,092	2,15E-46	7,80E-44	GO:0031224
INTEGRAL_TO_MEMBRANE	121	0,091	2,72E-41	4,95E-39	123	0,0925	2,93E-46	8,53E-44	GO:0016021
MULTICELLULAR_ORGANISMAL_DEVELOPMENT	119	0,1134	1,89E-50	9,17E-48	104	0,0991	9,02E-42	1,64E-39	GO:0007275
ANATOMICAL_STRUCTURE_DEVELOPMENT	115	0,1135	9,01E-49	3,27E-46	107	0,1056	1,44E-45	3,48E-43	GO:0048856
CYTOPLASM	110	0,0516	2,29E-17	1,23E-15	89	0,0418	4,54E-11	1,94E-09	GO:0005737
PLASMA_MEMBRANE_PART	107	0,0924	4,19E-37	6,09E-35	113	0,0976	9,97E-45	2,07E-42	GO:0044459
SYSTEM_DEVELOPMENT	104	0,1208	1,33E-46	3,86E-44	93	0,108	2,06E-40	3,33E-38	GO:0048731
INTRINSIC_TO_PLASMA_MEMBRANE	95	0,0959	2,99E-34	3,63E-32	97	0,0979	1,70E-38	2,48E-36	GO:0031226
INTEGRAL_TO_PLASMA_MEMBRANE	93	0,0952	2,68E-33	2,99E-31	96	0,0983	3,06E-38	4,05E-36	GO:0005887
BIOPOLYMER_METABOLIC_PROCESS	90	0,0534	3,12E-15	1,42E-13	74	0,0439	2,40E-10	8,69E-09	GO:0043283
PROTEIN_METABOLIC_PROCESS	68	0,0552	2,05E-12	7,43E-11	59	0,0479	7,30E-10	2,17E-08	GO:0019538
ORGAN_DEVELOPMENT	66	0,1156	1,11E-28	1,16E-26	61	0,1068	1,65E-26	1,85E-24	GO:0048513
CELLULAR_MACROMOLECULE_METABOLIC_PROCESS	63	0,0557	9,60E-12	2,97E-10	54	0,0477	4,44E-09	1,24E-07	GO:0044260
NERVOUS_SYSTEM_DEVELOPMENT	62	0,161	2,83E-35	3,74E-33	49	0,1273	6,99E-25	7,26E-23	GO:0007399
CELLULAR_PROTEIN_METABOLIC_PROCESS	62	0,0555	1,63E-11	4,65E-10	53	0,0474	7,62E-09	2,09E-07	GO:0044267
CYTOPLASMIC_PART	62	0,0448	5,77E-08	8,57E-07	55	0,0398	1,20E-06	1,84E-05	GO:0044444
POSITIVE_REGULATION_OF_BIOLOGICAL_PROCESS	59	0,0832	1,22E-18	7,69E-17	44	0,0621	4,66E-11	1,94E-09	GO:0048518
POSITIVE_REGULATION_OF_CELLULAR_PROCESS	58	0,0868	3,30E-19	2,18E-17	43	0,0644	2,44E-11	1,09E-09	GO:0048522
NEGATIVE_REGULATION_OF_BIOLOGICAL_PROCESS	56	0,0827	1,22E-17	7,11E-16	36	0,0532	1,34E-07	2,87E-06	GO:0048519
EXTRACELLULAR_REGION	55	0,123	2,02E-25	1,96E-23	47	0,1051	2,07E-20	1,88E-18	GO:0005576
CELL_PROLIFERATION_GO_0008283	55	0,1072	1,64E-22	1,40E-20	37	0,0721	2,47E-11	1,09E-09	GO:0008283
RECEPTOR_ACTIVITY	55	0,0943	6,55E-20	5,01E-18	53	0,0909	5,60E-20	4,52E-18	GO:0004872
NEGATIVE_REGULATION_OF_CELLULAR_PROCESS	54	0,0836	2,99E-17	1,55E-15	34	0,0526	3,76E-07	7,09E-06	GO:0048523
CELL_SURFACE_RECEPTOR_LINKED_SIGNAL_TRANSDUCTION_GO_0007166	52	0,0811	4,04E-16	1,96E-14	48	0,0749	6,47E-15	4,28E-13	GO:0007166
SYSTEM_PROCESS	51	0,0906	8,28E-18	5,02E-16	53	0,0941	1,17E-20	1,13E-18	GO:0003008
CELL_DEVELOPMENT	50	0,0867	1,07E-16	5,36E-15	45	0,078	1,07E-14	6,52E-13	GO:0048468
REGULATION_OF_CELLULAR_METABOLIC_PROCESS	50	0,0635	1,49E-11	4,34E-10	40	0,0508	9,04E-08	2,05E-06	GO:0031323
REGULATION_OF_METABOLIC_PROCESS	50	0,0626	2,55E-11	6,87E-10	40	0,0501	1,34E-07	2,87E-06	GO:0019222
BIOPOLYMER_MODIFICATION	47	0,0723	7,04E-13	2,69E-11	40	0,0615	4,54E-10	1,47E-08	GO:0043412
EXTRACELLULAR_REGION_PART	46	0,1361	2,64E-23	2,40E-21	41	0,1213	3,02E-20	2,59E-18	GO:0044421
CELL_CELL_SIGNALING	45	0,1114	2,66E-19	1,84E-17	42	0,104	3,38E-18	2,46E-16	GO:0007267
PROTEIN_MODIFICATION_PROCESS	45	0,0713	3,48E-12	1,15E-10	39	0,0618	6,63E-10	2,05E-08	GO:0006464
RESPONSE_TO_STRESS	42	0,0827	1,50E-13	6,23E-12	29	0,0571	5,27E-07	9,35E-06	GO:0006950
ANATOMICAL_STRUCTURE_MORPHOGENESIS	41	0,109	2,25E-17	1,23E-15	41	0,109	1,53E-18	1,17E-16	GO:0009653
TRANSMEMBRANE_RECEPTOR_ACTIVITY	41	0,0981	9,32E-16	4,37E-14	9	0,1765	6,68E-07	1,12E-05	GO:0004888
NEUROLOGICAL_SYSTEM_PROCESS	36	0,095	1,39E-13	5,96E-12	37	0,0976	2,57E-15	1,78E-13	GO:0050877
CELL_FRACTION	34	0,069	3,57E-09	7,01E-08	38	0,0771	1,83E-12	8,85E-11	GO:0000267
REGULATION_OF_DEVELOPMENTAL_PROCESS	33	0,075	7,49E-10	1,68E-08	28	0,0636	9,23E-08	2,06E-06	GO:0050793
TRANSFERASE_ACTIVITY_TRANSFERRING_PHOSPHORUS_CONTAINING_GROUPS	32	0,0755	1,16E-09	2,41E-08	38	0,0909	1,08E-14	6,52E-13	GO:0016772
REGULATION_OF_CELL_PROLIFERATION	31	0,1006	1,51E-12	5,63E-11	21	0,0682	1,22E-06	1,85E-05	GO:0042127
ENZYME_REGULATOR_ACTIVITY	31	0,096	5,24E-12	1,66E-10	30	0,0929	3,96E-12	1,86E-10	GO:0030234
ORGANELLE_ORGANIZATION_AND_BIOGENESIS	31	0,0655	5,51E-08	8,34E-07	8	0,1905	1,53E-06	2,27E-05	GO:0006996
REGULATION_OF_BIOLOGICAL_QUALITY	30	0,0716	1,27E-08	2,22E-07	38	0,0907	1,16E-14	6,76E-13	GO:0065008
PHOSPHOTRANSFERASE_ACTIVITY_ALCOHOL_GROUP_AS_ACCEPTOR	30	0,0898	5,98E-11	1,50E-09	11	0,275	2,45E-10	8,69E-09	GO:0016773
RESPONSE_TO_EXTERNAL_STIMULUS	26	0,0833	5,43E-09	1,04E-07	24	0,0769	2,28E-08	5,91E-07	GO:0009605
PROTEINACEOUS_EXTRACELLULAR_MATRIX	25	0,2551	4,44E-20	3,59E-18	19	0,1939	7,63E-14	4,26E-12	GO:0005578
EXTRACELLULAR_MATRIX	25	0,25	7,60E-20	5,53E-18	19	0,19	1,12E-13	6,00E-12	GO:0031012
SYNAPTIC_TRANSMISSION	24	0,1379	5,94E-13	2,33E-11	24	0,1379	1,16E-13	6,00E-12	GO:0007268

	Top 1000 Cluster 1 vs Cluster 2				Top 1000 GSCs and Schulte				
**Gene Set Name**	**# Genes in Overlap (k)**	**k/K**	**p-value**	**FDR q-value**	**# Genes in Overlap (k)**	**k/K**	**p-value**	**FDR q-value**	**GO term**
TRANSMISSION_OF_NERVE_IMPULSE	24	0,127	3,66E-12	1,18E-10	24	0,127	7,29E-13	3,66E-11	GO:0019226
EXTRACELLULAR_SPACE	24	0,098	8,56E-10	1,86E-08	24	0,098	1,86E-10	7,11E-09	GO:0005615
HYDROLASE_ACTIVITY_ACTING_ON_ESTER_BONDS	23	0,0855	2,53E-08	4,19E-07	22	0,0818	2,93E-08	7,34E-07	GO:0016788
CENTRAL_NERVOUS_SYSTEM_DEVELOPMENT	20	0,1626	2,22E-12	7,89E-11	18	0,1463	4,91E-11	1,98E-09	GO:0007417
CATION_BINDING	20	0,0939	4,36E-08	6,96E-07	22	0,1033	3,85E-10	1,30E-08	GO:0043169
ORGAN_MORPHOGENESIS	19	0,1319	3,27E-10	7,56E-09	19	0,1319	9,07E-11	3,56E-09	GO:0009887
TISSUE_DEVELOPMENT	17	0,1232	8,01E-09	1,46E-07	14	0,1014	7,32E-07	1,21E-05	GO:0009888
PHOSPHORIC_ESTER_HYDROLASE_ACTIVITY	17	0,1111	3,81E-08	6,15E-07	6	0,4	2,60E-07	5,10E-06	GO:0042578
REGULATION_OF_PROTEIN_KINASE_ACTIVITY	17	0,1097	4,62E-08	7,22E-07	15	0,0968	5,46E-07	9,46E-06	GO:0045859
REGULATION_OF_KINASE_ACTIVITY	17	0,1083	5,58E-08	8,37E-07	15	0,0955	6,44E-07	1,09E-05	GO:0043549
EXTRACELLULAR_MATRIX_PART	16	0,2807	4,55E-14	2,01E-12	10	0,1754	1,69E-07	3,52E-06	GO:0044420
GENERATION_OF_NEURONS	16	0,1928	2,42E-11	6,63E-10	11	0,1325	7,87E-07	1,27E-05	GO:0048699
SKELETAL_DEVELOPMENT	15	0,1456	5,97E-09	1,11E-07	16	0,1553	2,32E-10	8,66E-09	GO:0001501
PROTEIN_TYROSINE_KINASE_ACTIVITY	12	0,1905	8,70E-09	1,56E-07	10	0,1587	4,50E-07	8,27E-06	GO:0004713
NEURITE_DEVELOPMENT	11	0,2075	1,41E-08	2,43E-07	9	0,1698	9,39E-07	1,48E-05	GO:0031175
GLYCOSAMINOGLYCAN_BINDING	9	0,2647	3,01E-08	4,92E-07	8	0,2353	2,70E-07	5,24E-06	GO:0005539
POLYSACCHARIDE_BINDING	9	0,25	5,20E-08	7,96E-07	8	0,2222	4,35E-07	8,11E-06	GO:0030247
EXTRACELLULAR_MATRIX_STRUCTURAL_CONSTITUENT	8	0,2963	6,75E-08	9,91E-07	7	0,2593	7,49E-07	1,22E-05	GO:0005201

Supplementary Table S2. List of the antibodies used for RPPA analysis

Cat.#	Antibody	Company
9451	4E-BP1 (S65)	Cell Signaling Technology
9455	4E-BP1 (T70)	Cell Signaling Technology
3661	Acetyl-CoA Carboxylase (S79)	Cell Signaling Technology
3138	Ack1 (Y284)	Cell Signaling Technology
3137	Ack1 (Y857/858)	Cell Signaling Technology
9271	Akt (S473)	Cell Signaling Technology
9275	Akt (T308)	Cell Signaling Technology
2964	Akt2 (5B5)	Cell Signaling Technology
3343	ALK (Y1586)	Cell Signaling Technology
4184	AMPKalpha1 (S485)	Cell Signaling Technology
4181	AMPKBeta1 (S108)	Cell Signaling Technology
ab47563	Androgen Receptor (S650)	Abcam
07-1375	Androgen Receptor (S81)	Millipore
4431	A-Raf (S299)	Cell Signaling Technology
3761	ASK1 (S83)	Cell Signaling Technology
3761	ASK1 (S83)	Cell Signaling Technology
9225	ATF-2 (T69/71)	Cell Signaling Technology
4331	ATP-Citrate Lyase (S454)	Cell Signaling Technology
2853	ATR (S428)	Cell Signaling Technology
2853	ATR (S428)	Cell Signaling Technology
2914	Aurora A (T288)/B (T232)/C (T198) (D13A11)	Cell Signaling Technology
9292	Bad	Cell Signaling Technology
9291	Bad (S112)	Cell Signaling Technology
9295	Bad (S136)	Cell Signaling Technology
9297	Bad (S155)	Cell Signaling Technology
3814	Bak	Cell Signaling Technology
2772	Bax	Cell Signaling Technology
2872	Bcl-2	Cell Signaling Technology
2827	Bcl-2 (S70) (5H2)	Cell Signaling Technology
2762	Bcl-xL	Cell Signaling Technology
3738	Beclin-1 (part of Autophagy Ab Sampler #4445)	Cell Signaling Technology
MAB4376	Bmi-1 (10C7.2)	Chemicon
2696	B-Raf (S445)	Cell Signaling Technology
2864	c-Abl (T735)	Cell Signaling Technology
9661	Caspase-3, cleaved (D175)	Cell Signaling Technology
9761	Caspase-6, cleaved (D162)	Cell Signaling Technology
9491	Caspase-7, cleaved (D198)	Cell Signaling Technology
9501	Caspase-9, cleaved (D330)	Cell Signaling Technology
9561	Catenin (beta) (S33/37/T41)	Cell Signaling Technology
9565	Catenin (beta) (T41/S45)	Cell Signaling Technology
sc-11406	CD24 (FL-80)	SantaCruz
3570	CD44 (156-3C11)	Cell Signaling Technology
610265	CD45	BD Biosciences
9112	CDC2	Cell Signaling Technology
4539	CDC2 (Tyr15)	Cell Signaling Technology
4688	CDC25C (5H9)	Cell Signaling Technology
4901	CDC25C (Ser216)	Cell Signaling Technology
12302	Chk1 (S317)	Cell Signaling Technology
2348	Chk1 (S345)	Cell Signaling Technology
2661	Chk2 (Thr68)	Cell Signaling Technology
3073	c-Kit (Y703) (D12E12)	Cell Signaling Technology
3391	c-Kit (Y719)	Cell Signaling Technology
3313	Cofilin (S3) (77G2)	Cell Signaling Technology
610203	Cox-2 (33)	BD Biosciences
9421	C-Raf (S259)	Cell Signaling Technology
9427	C-Raf (S338) (56A6)	Cell Signaling Technology
9191	CREB (S133)	Cell Signaling Technology
3491	CrkII (Y221)	Cell Signaling Technology
3181	CrkL (Y207)	Cell Signaling Technology
4656	Cyclin A (BF683)	Cell Signaling Technology
4135	Cyclin B1 (V152)	Cell Signaling Technology
2926	Cyclin D1 (DCS6)	Cell Signaling Technology
3195	E-Cadherin	Cell Signaling Technology
2232	EGFR	Cell Signaling Technology

Cat.#	Antibody	Company
2238	EGFR (S1046/1047)	Cell Signaling Technology
2237	EGFR (Y1045)	Cell Signaling Technology
2234	EGFR (Y1068)	Cell Signaling Technology
4404	EGFR (Y1148)	Cell Signaling Technology
4407	EGFR (Y1173) (53A3)	Cell Signaling Technology
2235	EGFR (Y992)	Cell Signaling Technology
9741	eIF4E (S209)	Cell Signaling Technology
2441	eIF4G (S1108)	Cell Signaling Technology
9181	Elk-1 (S383)	Cell Signaling Technology
9575	eNOS (S113)	Cell Signaling Technology
2242	ErbB2/HER2	Cell Signaling Technology
4754	ErbB3/HER3 (1B2)	Cell Signaling Technology
4795	ErbB4/HER4 (111B2)	Cell Signaling Technology
9101	ERK 1/2 (p44/42 MAPK) (T202/Y204)	Cell Signaling Technology
M7047	Estrogen Receptor alpha	DAKO
2511	Estrogen Receptor alpha (S118) (16J4)	Cell Signaling Technology
2781	FADD (S194)	Cell Signaling Technology
611806	FAK (Y397)	BD Biosciences
3281	FAK (Y576/577)	Cell Signaling Technology
9461	FoxO1 (S256)	Cell Signaling Technology
9464	FoxO1 (T24)/FoxO3a (T32)	Cell Signaling Technology
3231	Gab1 (Y627)	Cell Signaling Technology
610565	GFAP	BD Biosciences
44-604	GSK-3 alpha/beta (S279/216)	Biosource/Invitrogen
9331	GSK-3alpha/beta (S21/9)	Cell Signaling Technology
2247	HER2/ErbB2 (Y1248)	Cell Signaling Technology
4561	HER3/ErbB3 (Y1197) (C56E4)	Cell Signaling Technology
4791	HER3/ErbB3 (Y1289) (21D3)	Cell Signaling Technology
610958	HIF-1alpha (54)	BD Biosciences
3377	Histone H3 (Ser10) (D2C8)XP	Cell Signaling Technology
3021	IGF-1 Rec (Y1131)/Insulin Rec (Y1146)	Cell Signaling Technology
3018	IGF-1 Receptor beta	Cell Signaling Technology
3024	IGF-1R (Y1135/36)/IR (Y1150/51) (19H7)	Cell Signaling Technology
9246	IkappaB-alpha (S32/36) (5A5)	Cell Signaling Technology
3025	Insulin Receptor beta (4B8)	Cell Signaling Technology
2386	IRS-1 (S612)	Cell Signaling Technology
3331	Jak1 (Y1022/1023)	Cell Signaling Technology
3841	LIMK1 (T508)/LIMK2 (T505)	Cell Signaling Technology
3055	LKB1 (S334)	Cell Signaling Technology
3482	LKB1 (S428)	Cell Signaling Technology
2741	MARCKS (S152/156)	Cell Signaling Technology
3521	MDM2 Ser166	Cell Signaling Technology
9121	MEK1/2 (S217/221)	Cell Signaling Technology
3126	Met (Y1234/1235)	Cell Signaling Technology
2739	MGMT	Cell Signaling Technology
9594	MSK1 (S360)	Cell Signaling Technology
3681	Mst1 (T183)/Mst2 (T180)	Cell Signaling Technology
2971	mTOR (S2448)	Cell Signaling Technology
2974	mTOR (S2481)	Cell Signaling Technology
3580	Nanog	Cell Signaling Technology
4061	N-Cadherin	Cell Signaling Technology
3031	NF-kappaB p65 (S536)	Cell Signaling Technology
07-1231	Notch1	Millipore
ab14140	NUMB	Abcam
4824	p16 INK4A	Cell Signaling Technology
2947	p21 Waf1/Cip1 (12D1)	Cell Signaling Technology
71-7700	p27 (T187)	Life Technologies/Invitrogen
9211	p38 MAP Kinase (T180/Y182)	Cell Signaling Technology
9284	p53 (S15)	Cell Signaling Technology
9208	p70 S6 Kinase (S371)	Cell Signaling Technology
9205	p70 S6 Kinase (T389)	Cell Signaling Technology
07-018	p70 S6 Kinase (T412)	Millipore
9341	p90RSK (S380)	Cell Signaling Technology
2605	PAK1 (S199/204)/PAK2 (S192/197)	Cell Signaling Technology
9541	PARP, cleaved (D214)	Cell Signaling Technology
2992	PDGF Receptor alpha (Y754) (23B2)	Cell Signaling Technology

Cat.#	Antibody	Company
3161	PDGF Receptor beta (Y751)	Cell Signaling Technology
07-021	PDGFR beta (Y716)	Upstate/Millipore
3062	PDK1	Cell Signaling Technology
3061	PDK1 (S241)	Cell Signaling Technology
4781	PKA C (T197)	Cell Signaling Technology
9371	PKC (pan) (betaII S660)	Cell Signaling Technology
2056	PKC alpha	Cell Signaling Technology
06-822	PKC alpha (S657)	Upstate/Millipore
9375	PKC alpha/beta II (T638/641)	Cell Signaling Technology
9374	PKC delta (T505)	Cell Signaling Technology
9377	PKC theta (T538)	Cell Signaling Technology
9378	PKC zeta/lambda (T410/403)	Cell Signaling Technology
2821	PLCgamma1 (Y783)	Cell Signaling Technology
558400	PLK1 (T210)	BD Biosciences
2039	PP2A A Subunit	Cell Signaling Technology
4953	PP2A B Subunit	Cell Signaling Technology
44-1100	PRAS40 (T246)	Life Technologies/Invitrogen
2611	PRK1 (T774)/PRK2 (T816)	Cell Signaling Technology
3171	Progesterone Receptor (S190)	Cell Signaling Technology
9552	PTEN	Cell Signaling Technology
9551	PTEN (S380)	Cell Signaling Technology
3291	Pyk2 (Y402)	Cell Signaling Technology
sc-9072	RANK	Santa Cruz Biotechnology
NBP1-31140	RANKL	Novus Biologicals
3321	Ras-GRF1 (S916)	Cell Signaling Technology
3221	Ret (Y905)	Cell Signaling Technology
A300-245A	RPA32 (S4/8)	Bethyl Labs
ab109394	RPA32 (T21)	Abcam
9348	RSK3 (T356/S360)	Cell Signaling Technology
H00006278-A01	S100A7 calcium binding protein	Abnova
4856	S6 Ribosomal Protein (S235/236) (2F9)	Cell Signaling Technology
2215	S6 Ribosomal Protein (S240/244)	Cell Signaling Technology
9251	SAPK/JNK (T183/Y185)	Cell Signaling Technology
9155	SEK1/MKK4 (S80)	Cell Signaling Technology
5599	SGK (S78) (D36D11)	Cell Signaling Technology
07-206	Shc (Y317)	Millipore
3941	SHIP1 (Y1020)	Cell Signaling Technology
44-558	SHP2 (Y580)	Biosource
2954	Smac/Diablo	Cell Signaling Technology
9511	SMAD1/5/9_pS463-65/pS463-65/pS465-67	Cell Signaling Technology
3104	Smad2 (S245/250/255)	Cell Signaling Technology
3101	Smad2 (S465/467)	Cell Signaling Technology
2105	Src (Y527)	Cell Signaling Technology
2101	Src Family (Y416)	Cell Signaling Technology
9171	Stat1 (Y701)	Cell Signaling Technology
9134	Stat3 (S727)	Cell Signaling Technology
9145	Stat3 (Y705) (D3A7)	Cell Signaling Technology
9351	Stat5 (Y694)	Cell Signaling Technology
9361	Stat6 (Y641)	Cell Signaling Technology
2808	Survivin (71G4)	Cell Signaling Technology
2711	Syk (Y525/526)	Cell Signaling Technology
3709	TGF-Beta (56E4)	Cell Signaling Technology
3614	Tuberin/TSC2 (Y1571)	Cell Signaling Technology
9321	Tyk2 (Y1054/1055)	Cell Signaling Technology
3111	VASP (S157)	Cell Signaling Technology
44-488	Vav3 (Y173)	Biosource
2479	VEGF Receptor 2 (55B11)	Cell Signaling Technology
2471	VEGFR 2 (Y951)	Cell Signaling Technology
5741	Vimentin xp	Cell Signaling Technology
4910	WEE1 S642 (D47G5)	Cell Signaling Technology
2042	XIAP Antibody	Cell Signaling Technology
2701	Zap-70 (Y319)/Syk (Y352)	Cell Signaling Technology

Supplementary Table S3. List of the RPPA results on GSC subtypes

Subtype	p.value	Log2.Gsf.to.Gsr.Ratio	Neg.Log10.p.value	Antibody
GSf-like	0.624206114766406	0.542470881261485	0.204671981270973	ACC1_pS79
GSf-like	0.141644690295137	0.329786435799689	0.848799700838165	Ack1_pY857-58
GSf-like	0.276366613750387	0.207747474917617	0.558514422729609	ALK_pY1586
GSf-like	0.588178896568752	0.0361775438650872	0.23049056171973	AMPKa_pS485
GSf-like	0.00202135516573954	0.258138377240422	2.69435737130955	AMPK-beta_pS108
GSf-like	0.000000002452070998	5.63699370386942	8.61046696057176	Androgen-Rec_pS650
GSf-like	0.0322206525061268	0.189602145900196	1.49186566885346	Androgen-Rec_pS81
GSf-like	0.102470434859749	0.323163689448546	0.98940142074879	Ask1_pS83
GSf-like	0.0696424047983282	2.31082763690692	1.1571262408199	ATR_pS428
GSf-like	0.974048246325889	0.015888661447244	0.011419531217714	BAD_pS155
GSf-like	0.000948657669920028	0.317888159704612	3.022890477653	BAK
GSf-like	0.0505020068873414	0.540154705362481	1.29669136321242	BAX
GSf-like	0.000542324050470055	0.5871607261059	3.26574113545976	BCL2
GSf-like	0.870282772281036	0.12430610677069	0.0603396134871364	CASP6_cl-D162
GSf-like	0.102470434859749	0.223906553251299	0.98940142074879	CD24
GSf-like	0.624053969495896	0.188611507461895	0.204777849995913	CD45
GSf-like	0.150926950066716	1.15780050673711	0.821233204093055	CDC2
GSf-like	0.0963036920286884	1.68759553346281	1.01635706285521	CDC2_pY15
GSf-like	0.939742989577073	1.28685221889401	0.0269909054112759	CDC25C
GSf-like	0.76236881846984	1.36974049970705	0.117834875018498	CDC25C_pS216
GSf-like	0.449691797968891	1.2351829242675	0.347085033555776	Chk1_pS317
GSf-like	0.489846811228207	0.413767054330187	0.309939714746871	CHK1_pS345
GSf-like	0.256839257957857	4.07703646704674	0.59033859348718	Chk2_pT68
GSf-like	0.865235143189356	0.019304553456968	0.0628658491690861	c-Kit_pY719
GSf-like	0.02224312059135	1.12978048124159	1.65280428361284	c-Met_pY1234-35
GSf-like	0.0279222369883332	0.402795447599095	1.55404979117882	Cofilin_pS3
GSf-like	0.752607234080857	0.250888183685556	0.12343161154592	c-RAF_pS259
GSf-like	0.0467924763442644	0.422010288199458	1.32982397053359	c-RAF_pS338
GSf-like	0.414216178242525	0.339925835702724	0.382772942660716	CREB_pS133
GSf-like	0.437846436294425	0.0868072919912257	0.358678180737542	Crk II_pY221
GSf-like	0.000293895807163214	0.904434042924398	3.53180660970442	CrkL_pY207
GSf-like	0.76236881846984	2.764857472448	0.117834875018498	Cyclin B1
GSf-like	0.25110745743941	0.0555466938645112	0.600140389331314	EGFR_pY1045
GSf-like	0.191418425237607	0.568494741061831	0.718016260948477	Elk_pS383
GSf-like	0.000921840759821713	0.7213863003365	3.03534409315845	Elk1_pS383
GSf-like	0.437741792646151	0.127029382313854	0.358781987887354	eNOS-NOS III_pS116
GSf-like	0.252332231945352	2.0789235319069	0.598027270838216	ER-alpha
GSf-like	0.252999061474684	0.13372387617422	0.596881089879999	ER-alpha_pS118
GSf-like	0.00630473003017507	2.06644629263305	2.20033350527099	ERK1-2_pT202-pY204
GSf-like	0.0603890813092702	0.471822915055563	1.21904157720323	FADD_pS194
GSf-like	0.743971478075057	0.284697850500939	0.128443713852043	FAK_pY397
GSf-like	0.0337629775534036	0.499865520304	1.47155925997321	FAK_pY576-77
GSf-like	0.02224312059135	0.493111864776486	1.65280428361284	FoxO1_pS256
GSf-like	0.130570018115736	0.709329462998312	0.88415653563166	FoxO1-FoxO3a_pT24-pT32
GSf-like	0.0603890813092702	1.14968254787213	1.21904157720323	Gab1_pY627
GSf-like	0.513444337525733	0.14081351937262	0.2895066313573	GFAP
GSf-like	0.388641803740222	0.268437188487767	0.410450486913669	GSK3alpha-beta_pS21-9
GSf-like	0.122591069820236	0.201708179680378	0.911541164964827	GSK3alpha-beta_pS279-pS216
GSf-like	0.0113692653407269	0.606850044306503	1.9442675976497	HER3
GSf-like	0.220266184038241	0.61865270078518	0.657052171995222	HER3_pY1197
GSf-like	0.00001021210824703	0.603060624652418	4.99088459037783	HER3_pY1289
GSf-like	0.220671361919847	0.229124379331787	0.656254024641652	HER4
GSf-like	0.80649594050734	0.308040940651147	0.0933978142882851	HIF-1alpha
GSf-like	0.0274863361115103	0.302491587883531	1.56088314711912	Histone H3_pS10
GSf-like	0.0337629775534036	1.80725774425968	1.47155925997321	IGF1-R beta_pY1131/IR-beta_pY1146
GSf-like	0.0499430029124159	0.296478521140044	1.30152534846677	IGF1-R beta_pY1135-36/IR-beta_pY1150-51
GSf-like	0.00191772342809893	1.62571506869365	2.71721402610769	IGF1-R-beta

Subtype	p.value	Log2.Gsf.to.Gsr.Ratio	Neg.Log10.p.value	Antibody
GSf-like	0.165123590128394	0.585225783914597	0.782190877489892	IR-beta
GSf-like	0.00615097275495091	0.629237321365931	2.21105619663105	IRS1_pS612
GSf-like	0.0337629775534036	0.425736186244146	1.47155925997321	Jak1_pY1022-23
GSf-like	0.327186877790306	0.241071873856688	0.485204122535982	LIMK1-2_pT508-pT505
GSf-like	0.0724480135297871	0.15661125591872	1.13997351805864	LKB1_pS334
GSf-like	0.000000421442044818	3.73069049155611	6.37526213953589	MARKCS_pS152-56
GSf-like	0.00815097159350264	1.3101581263522	2.08879062039533	MDM2_pS166
GSf-like	0.166791824190023	0.0148956849419327	0.777825241445169	MEK1-2_pS217-221
GSf-like	0.934925311642117	0.180843250428743	0.0292230822154962	MSK1_pS360
GSf-like	0.414216178242525	0.015157068300034	0.382772942660716	Mst1-2_pT183_pT180
GSf-like	0.934925311642117	0.0635509264062201	0.0292230822154962	Nanog
GSf-like	0.191418425237607	0.481503816666006	0.718016260948477	N-Cadherin
GSf-like	0.934925311642117	0.200884309393851	0.0292230822154962	Numb
GSf-like	0.820595839755441	0.216153608541119	0.085870689143217	p16/INK4A
GSf-like	0.0696424047983282	2.64666670657285	1.1571262408199	p21
GSf-like	0.414216178242525	0.221517569696993	0.382772942660716	p27_pT187
GSf-like	0.288487463323489	1.63715203604652	0.539873055048043	p53_pS15
GSf-like	0.164938177051956	0.575634875535758	0.782678809704082	PAK1-2_pS199/pS204-pS192-97
GSf-like	0.0696424047983282	1.99353388873893	1.1571262408199	PARP_cl-D214
GSf-like	0.414011337165912	0.272028667445907	0.382987766122625	PDGFR-beta_pY716
GSf-like	0.513629113393124	0.470093486778866	0.289350367673548	PDGFR-beta_pY751
GSf-like	0.364346126633553	2.44541354881346	0.438485843102591	PDK1
GSf-like	0.0677921964819661	0.351299453772925	1.16882029462657	PDK1_pS241
GSf-like	0.256839257957857	2.00985936488141	0.59033859348718	PKC-alpha
GSf-like	0.00343801772137642	0.488937099551504	2.46369188905788	PKC-theta_pT538
GSf-like	0.124647197171303	0.109504643416696	0.904317482637323	PLC-gamma_pY783
GSf-like	0.173617334424943	2.18520926093986	0.760406915857296	PLK1_pT210
GSf-like	0.567458527195413	0.420239559399604	0.246065873462326	PP2A-A
GSf-like	0.0724480135297871	0.760274432382884	1.13997351805864	PRK1-2_pT774-pT816
GSf-like	0.369107590437563	0.270661013173676	0.43284702375245	Progesteron-Rec_pS190
GSf-like	0.0653906218951268	3.72242180461113	1.18448453227575	PTEN
GSf-like	0.0754206899955867	3.0207016625207	1.122509498715	PTEN_pS380
GSf-like	0.0412268333371637	2.20065184992092	1.3848200223903	Pyk2_pY402
GSf-like	0.356883929754839	0.187560340258789	0.447473007594714	RANK
GSf-like	0.252999061474684	0.236608527413678	0.596881089879999	Ras-GRF1_pS916
GSf-like	0.102470434859749	0.560670991002893	0.98940142074879	Ret_pY905
GSf-like	0.28991845394257	0.989937099241685	0.537724139973207	RPA32_pS4-8
GSf-like	0.0963036920286884	0.517085480574462	1.01635706285521	RPA32_pT21
GSf-like	0.22046883962008	0.129465773567845	0.656652783689787	S100A7
GSf-like	0.252999061474684	0.334711559049444	0.596881089879999	SAP-JNK_pT183-pY185
GSf-like	0.220671361919847	0.198401459888148	0.656254024641652	SEK1-MKK4_pS80
GSf-like	0.120819386535671	0.997034954510683	0.917863373745183	Shc_pY317
GSf-like	0.0178922676130193	1.60995510161831	1.74733461474774	SHIP1_pY1020
GSf-like	0.220671361919847	0.149030294706233	0.656254024641652	SHIP2_pY580
GSf-like	0.0412268333371637	0.27533960721362	1.3848200223903	SMAD2_pS245-50-55
GSf-like	0.740164126294547	0.638971168885988	0.130671967781668	SMAD2_pS465-67
GSf-like	0.0336849623858166	1.13830515916139	1.47256393326429	SRC_pY527
GSf-like	0.0274863361115103	2.54639121204951	1.56088314711912	SRC-Family_pY416
GSf-like	0.80649594050734	0.495188577714001	0.0933978142882851	STAT3_pY715
GSf-like	0.683091398309609	1.09823991419337	0.165521183396262	STAT5_pY964
GSf-like	0.220671361919847	0.649607151477012	0.656254024641652	STAT6_pY641
GSf-like	0.0862727671159231	0.183222094374238	1.06412627216145	Syk_pY525-26
GSf-like	0.0607850135646411	0.09722488570739	1.2162034820504	TSC2_pY1571
GSf-like	0.0724480135297871	0.917840494863963	1.13997351805864	Tyk2_pY1054-55
GSf-like	0.0178922676130193	0.799600240839286	1.74733461474774	Vav3_pY173
GSf-like	0.327186877790306	0.116749196847255	0.485204122535982	VEGFR2_pY951
GSf-like	0.0724480135297871	2.45366808615426	1.13997351805864	Vimentin
GSf-like	0.00650170237308179	0.298168287790962	2.18697291498242	WEE1_pS642
GSf-like	0.0864107329737001	0.370957932749669	1.06343231097302	Zap70/Syk_pY319/pY352

Subtype	p.value	Log2.Gsf.to.Gsr.Ratio	Neg.Log10.p.value	Antibody
GSr-like	0.0342937210364928	-2.77450283652777	1.46478538925253	4EBP1_pS65
GSr-like	0.000716283240943033	-1.04797220481354	3.14491521001841	4EBP1_pT70
GSr-like	0.513629113393124	-0.0245764596703393	0.289350367673548	Ack1_pY284
GSr-like	0.0200186716025004	-1.51094936945524	1.69856474478102	AKT_pS473
GSr-like	0.0412500165939395	-0.236039265746938	1.38457587240727	AKT_pT308
GSr-like	0.138138705867788	-0.702592468709614	0.859684616934991	AKT2
GSr-like	0.0178922676130193	-1.83123536527172	1.74733461474774	a-RAF_pS299
GSr-like	0.111629712759795	-0.461661972830762	0.952220192763026	ATF-2_pT69-71
GSr-like	0.368687217419712	-0.389239114591682	0.43334191935162	ATP-Citrate Lyase_pS454
GSr-like	0.1150901699032	-0.180441346958489	0.938961768803837	Aurora A-B-C_pT288-pT232-pT198
GSr-like	0.476985710858319	-0.246628957963576	0.321494630998324	BAD
GSr-like	0.0143058784354296	-1.04503643789794	1.84448546970445	BAD_pS112
GSr-like	0.252373955255636	-0.0936129660536288	0.597955465879514	BAD_pS136
GSr-like	0.0846778526715009	-0.993407207358624	1.07223016369871	BCL2_pS70
GSr-like	0.140945535250473	-0.625904445693318	0.85094867677566	Bcl-XL
GSr-like	0.252999061474684	-0.0822338485546103	0.596881089879999	Beclin 1
GSr-like	0.0603890813092703	-0.261452850836346	1.21904157720323	BMI1
GSr-like	0.347534051486669	-0.149985253975795	0.459002636690406	b-RAF_pS445
GSr-like	0.624206114766406	-0.352853539727782	0.204671981270973	c-Abl_pT735
GSr-like	0.414216178242525	-0.11714678087198	0.382772942660716	CASP3_cl-D175
GSr-like	0.462432726450476	-0.250243912301141	0.334951438398261	CASP7_cl-D198
GSr-like	0.620728235085189	-0.950735916373748	0.207098499398233	CASP9_cl-D330
GSr-like	0.327186877790306	-0.0605214855098365	0.485204122535982	Catenin-beta_pS33-37/pT41
GSr-like	0.922254442510852	-0.0465120931636241	0.0351492440943256	Catenin-beta_pT41-pS45
GSr-like	0.00247524451516783	-2.27513028310955	2.60638189315751	CD44
GSr-like	0.602713044956532	-0.722005029372048	0.219889408679212	c-Kit_pY703
GSr-like	0.462432726450476	-1.36334706129499	0.334951438398261	COX2
GSr-like	0.414216178242525	-0.207701310445459	0.382772942660716	Cyclin A
GSr-like	0.0412500165939395	-2.05401433636066	1.38457587240727	Cyclin D1
GSr-like	0.342361452442926	-0.431366196963496	0.465515139829445	E-Cadherin
GSr-like	0.177134832869638	-0.144834020479482	0.751696028106528	EGFR
GSr-like	0.252999061474684	-0.0774438217427535	0.596881089879999	EGFR_pS1046-47
GSr-like	0.320211942662985	-0.220218628264413	0.49456247461562	EGFR_pY1068
GSr-like	0.0336849623858167	-0.145653530896179	1.47256393326429	EGFR_pY1148
GSr-like	0.087654818737443	-0.262581193331398	1.05722420400194	EGFR_pY1173
GSr-like	0.845245286880015	-0.958731675103084	0.073017242198051	EGFR_pY992
GSr-like	0.0970949827939282	-0.946720863238901	1.01280321088712	eIF4E_pS209
GSr-like	0.0274863361115104	-1.05741193565842	1.56088314711911	eIF4G_pS1108
GSr-like	0.425439298451231	-0.535114591747368	0.371162396162871	ERBB2_pY1248
GSr-like	0.967406958095214	-0.163538708878056	0.0143907932639905	HER2
GSr-like	0.934925311642117	-0.0337069060686396	0.0292230822154961	IkB-alpha_pS32-36
GSr-like	0.934925311642117	-0.128942273100087	0.0292230822154962	Lck_pY505
GSr-like	0.165123590128394	-0.34272606826605	0.782190877489892	LKB1_pS428
GSr-like	0.141644690295137	-0.913019141836132	0.848799700838165	MGMT
GSr-like	0.130570018115736	-0.80229755836415	0.88415653563166	mTOR_pS2448
GSr-like	0.0846778526715009	-0.714199638409317	1.07223016369871	mTOR_pS2481
GSr-like	0.381655060067704	-0.361794416969236	0.418328975271343	NF-kB_pS536
GSr-like	0.934696282916467	-0.0432804659855323	0.0293294844014081	Notch1
GSr-like	0.00841257864773183	-1.28782366035058	2.07507086260556	p38MAPK_pT180-pY182
GSr-like	0.00005328243556103	-0.557843374372958	4.27341593161289	p70S6K_pS371
GSr-like	0.0684913393125574	-0.803713616230723	1.1643643413041	p70S6K_pT389
GSr-like	0.0495458564244081	-0.360740570704829	1.30499266019508	p70S6K_pT412
GSr-like	0.0534244001216966	-0.419029008976919	1.27226034563183	p90RSK_pS380
GSr-like	0.934925311642117	-0.0281270375432749	0.0292230822154961	PDGFR-alpha_pY754
GSr-like	0.0723227862872007	-0.53955111825108	1.14072485070406	PKA-c_pT197
GSr-like	0.0504982714526689	-0.711012898489739	1.29672348745392	PKC alpha-betaII_pT638-41
GSr-like	0.0575368548937279	-0.387338961044078	1.24005388140346	PKC pan-betaII_pS660
GSr-like	0.0450512779833448	-0.448091469224347	1.34629288474613	PKC-alpha_pS657
GSr-like	0.540113611871945	-0.0237898560028277	0.267514877548686	PKC-delta_pT505

Subtype	p.value	Log2.Gsf.to.Gsr.Ratio	Neg.Log10.p.value	Antibody
GSr-like	0.02224312059135	-0.25749949857592	1.65280428361284	PKC-zeta/lambda_pT410/pT403
GSr-like	0.369107590437563	-0.11177888197135	0.43284702375245	PP2A-B
GSr-like	0.000815039911826899	-1.15777706142994	3.08882112369956	PRAS40_pT246
GSr-like	0.82097961008484	-0.000134673982911191	0.085667628919054	RANKL
GSr-like	0.414216178242525	-0.524966925494084	0.382772942660716	RSK3_pT356-pS360
GSr-like	0.00309771927983606	-1.05770984775107	2.50895794123855	S6RP_pS235-36
GSr-like	0.00882990920381388	-0.512175688801361	2.05404376216256	S6RP_pS240-44
GSr-like	0.369107590437563	-0.066363256141505	0.43284702375245	SGK1_pS78
GSr-like	0.80649594050734	-0.171321330456613	0.0933978142882851	Smac-Diablo
GSr-like	0.567628499263336	-0.347501819413845	0.245935807784528	SMAD1/5/9_pS463-65/pS463-65/pS465-67
GSr-like	0.513629113393124	-0.104077125665464	0.289350367673548	STAT1_pY701
GSr-like	0.580239836981834	-0.728790582115471	0.236392457561166	STAT3_pS727
GSr-like	0.0377790289768611	-0.468905228572707	1.42274920881256	Survivin
GSr-like	0.409770292005494	-0.0936264961982764	0.387459530770753	TGF-beta
GSr-like	0.0724480135297872	-0.54313395135119	1.13997351805863	VASP_pS157
GSr-like	0.00002130165805921	-1.03868046816406	4.67158659102415	VEGFR2
GSr-like	0.743971478075057	-0.0424964372955466	0.128443713852043	XIAP

Supplementary Table S4. Clinical parameters

**Parameter**	**GSCs full**	**GSCs restricted**	***p value***
Age (mean ± SD)	58.2 ± 11.7	58.9 ± 10.8	0.8451^
Sex			
Males, *n*(%)	8/10 (80)	4/7 (57.1)	0.5928*
Females, *n*(%)	2/10 (20)	3/7 (42.9)	
Tumor Location			0.1574§
Frontal, *n*(%)	3/10 (30)	3/7 (42.9)	0.6437*
Temporal, *n*(%)	1/10 (10)	1/7 (14.3)	>0.9999*
Parietal, *n*(%)	6/10 (60)	1/7 (14.3)	0.1340*
Occipital, *n*(%)	0/10 (0)	2/7 (28.6)	0.1544*
Tumor diameter (mean ± SD)	4.1 ± 1.8	4.9 ± 1.2	0.3834^
Symptom duration (mean ± SD)	8.3 ± 12.0	1.9 ± 1.1	0.0422^
Extent of resection			
Gross total removal, *n*(%)	9/10 (90)	6/7 (85.7)	>0.9999*
Partial removal, *n*(%)	1/10 (10)	1/7 (14.3)	
KPS (mean ± SD)	79.0 ± 8.8	71.4 ± 6.9	0.0529^

*, Fisher exact test; ^, Mann-Whithey *U* test; §, Chi-square test

Supplementary Table S5. Molecular parameters

**Parameter**	**GSCs full**	**GSCs restricted**	***p value***
MGMT status			
methylated, *n*(%)	3/10 (30)	3/7 (42.9)	0.6437*
unmethylated, *n*(%)	7/10 (70)	4/7 (57.1)	
EGFRvIII status			
positive, *n*(%)	1/10 (10)	5/7 (71.4)	0.0345*
negative, *n*(%)	9/10 (90)	2/7 (28.6)	
PTEN status			
normal, *n*(%)	6/9 (66.7)	5/7 (71.4)	>0.9999*
hypoexpressed, *n*(%)	3/9 (33.3)	2/7 (28.6)	
VEGF status			
normal, *n*(%)	1/10 (10)	1/7 (14.3)	>0.9999*
hyperexpressed, *n*(%)	9/10 (90)	6/7 (85.7)	
Ki67 (mean $\pm$ SD)	25.0 $\pm$ 20.3	24.7 $\pm$ 11.0	0.6234^
GBM subtype			0.4547§
neural, n(%)	3/9 (33.3)	1/6 (16.7)	0.6044*
proneural, n(%)	1/9 (11.1)	0/6 (0)	>0.9999*
classic, n(%)	5/9 (55.6)	4/6 (66.7)	>0.9999*
mesenchymal, n(%)	0/9 (0)	1/6 (16.7)	0.4*

*, Fisher exact test; ^, Mann-Whithey *U* test; §, Chi-square test

Supplementary Table S6. List of GSf- and GSr-like genes for interrogation of GBM-BioDP database

		GBM-BioDP/TCGA	*RPPA GSCs/RPPA TCGA*
	Gene Symbol	Overlap with RPPA	Signature Concordance (P/GSf Vs M/GSr)
GSf-like	ERBB3 (HER3)	T+p	1/2 (pY1289)
	IGF1R	T	
	INSR	N.A.	N.A.
	IRS1	T	N.A.
	FOXO1	N.A.	N.A.
	PRKAA1 (AMPK)	T+p	N.A.
	CRAF	T+p	
	MAPK3 (ERK1)	p	
	PTK2 (FAK)	T	N.A.
	SRC	T+p	1/3 (pS527)
	SMAD2	N.A.	N.A.
GSr-like	EIF4G1	N.A.	N.A.
	EIF4EBP1	T+p	
	RPS6	T+p	1/3 (pS235-36)
	RPS6KB1	T+p	
	MTOR	T+p	N.A.
	AKT1S1 (PRAS40)	p	
	AKT1	T+p	
	BAD	N.A.	N.A.
	EGFR	T+p	3/3

T=Total protein
p=phosphorylated
N.A.=not available

Not significant or opposite
Not significant trend
Significant agreement

Supplementary Table S7 – Association of RPPA profiles obtained by RPPA on GSC with expression and clinical data in TCGA database.

			Analysis on RPPA data from TCGA database								
			Expression higher in			association with longer OS in			association with shorter OS in		
			P subtype	M subtype		P subtype	M subtype	total popul.	P subtype	M subtype	total popul.
RPPA profiles on GSC	GSf-like	HER3 pY1289 (Y)	p=0.00016		*Cox*	p=0.426	p=0.9059	p= 0.01			
					Cox - HR	p=0.4274	p=0.9059	p= 0.0316			
		SRC pS527 (Y)	p=0.00031		*Cox*	p=0.8111	p=0.1961	p=0.0835			
					Cox - HR	p=0.8114	p=0.198	p=0.3696			
		FAK		p=0.00241	*Cox*				p=0.0243	p=0.3877	p=0.0656
					Cox - HR				p=0.0278	p=0.3899	p=0.2245
	GSr-like	RPS6 pS235-36 (Y)		p=0.00761	*Cox*		p=0.719		p=0.0428		p=0.0466
					Cox - HR		p=0.72		p=0.0405		p=0.1376
		EIF4EBP1	p=0.21907		*Cox*	p=0.1507		p=0.0489		p=0.4472	
					Cox - HR	p=0.1476		p=0.1533		p=0.4521	
		EIF4EBP1 pS65	p=0.00053		*Cox*	p=0.0071		p=0.0206		p=0.4921	
					Cox - HR	p=0.0078		p=0.0477		p=0.4948	
		AKT pS473	p=0.00493		*Cox*		p=0.2653		p=0.0349		p=0.1044
					Cox - HR		p=0.2628		p=0.0366		p=0.6198
		EGFR pY1173	p=0.20243		*Cox*	p=0.0168				p=0.2133	p=0.0944
					Cox - HR	p=0.0162				p=0.2153	p=0.4707
		EGFR pY992	p=0.64183		*Cox*	p=0.0201	p=0.7278				p=0.1119
					Cox - HR	p=0.0196	p=0.7273				p=0.7852

Cox p values are intended for logrank test

Cox - HR p values are intended for hazard ratios of expression above the median values in the relative cohort (overall or subtype)

Supplementary Table S8. List of GSf-like genes for simplified mPI model

Protein	HR	Lower 95% CI	Upper 95% CI	p value for expression HR
FAKrppa	1.3366	0.9447	1.8910	0.1012
HER3RV	0.9373	0.6011	1.4618	0.7754
HER3.pY1289RC	0.2871	0.1052	0.7829	0.0147
SRCMV	0.2363	0.0544	1.0255	0.0540
SRC.pY416RC	0.8321	0.4587	1.5096	0.5454
SRC.pY527RV	0.6928	0.3306	1.4518	0.3310

Legend to Supplementary Figures

Supplementary Fig. S1

A) Unsupervised hierarchical clustering of the combined dataset containing GSCs, 173 Verhaak GBM samples and the Schulte clones, using 674 genes from the Verhaak signature. Differences in expression levels are in log2 scale (red +3, black=0, green= -3).

B) Overlap between the top 1000 most variable genes in Cluster 1 vs Cluster 2 and the top 1000 most variable genes in the combined dataset. Gene Ontology (GO) gene sets present in only one of the two lists are reported (the full list of GSEA results on GO Gene sets is available in Supplementary Table S1d).

Supplementary Fig. S2

Unsupervised hierarchical clustering of TCGA RNAseq data for GBM. Sample annotation on top of the clustered heatmap, comprises several relevant mutations and legend showing the color code and frequency of GBM subtypes is reported on the top-left box. Heatmap was obtained from the Pan-Cancer NG-CHM Compendium website (see methods for details) and shows RNA expression (Illumina, HiSeq) for 447 cancer interesting genes in 167 TCGA GBM samples, using the TCGA Pan-Cancer freezeV4 data. Z normalized expression values are color coded as follows: green < -2.5, black = 0, red > 2.5.

Supplementary Fig. S3

Volcano Plot of RPPA data produced on GSf- and GSr-like GSCs showing, for every antibody tested, the log2 of the ratio between GSf- and GSr-like intensity against the antilogarithm (base 10) of the p value obtained by Wilcoxon rank sum non-parametric test. Names of most statistically significant endpoints (dashed lines thresholds for p values) are reported on the plot.

Supplementary Fig. S4

Unsupervised hierarchical clustering of TCGA RPPA data for GBM. Sample annotation on top of the clustered heatmap, comprises several relevant mutations and legend showing the color code and frequency of GBM subtypes is reported on the top-left box. Heatmap was obtained from the Pan-Cancer NG-CHM Compendium website (see methods for details) and shows RPPA results for 181 variable proteins in 215 TCGA GBM samples, using the TCGA Pan-Cancer freezeV4 data. Z normalized RPPA values are color coded as follows: green < -2.5, black = 0, red > 2.5.

Supplementary Fig. S5

Table plots of hazard ratios (HR) for stratified expression levels of individual RPPA analytes, selected as distinctive of GSf- (**A**) and GSr-like (**B**) groups, in P (n=41) and M (n=29) subtypes of TCGA GBM samples. For each RPPA endpoint and subtype, a new binary variable was defined ('*Med.Expr*') to stratify patients based on expression levels

above (+) or below (-) the sample median. Subsequently, this new variable was used as covariate in a Cox regression model. HR, confidence intervals (95% CI), logrank test and expression p values refer to ‘*Med.Expr*’+ group.

Supplementary Fig. S6

The mPI was calculated (see methods section) in the full cohort of TCGA GBM patients, in the P and in the M subtypes using all GSf- (**A**) and GSr-like (**B**) RPPA endpoints, respectively as covariates of two different Cox models. A new binary variable was defined (‘*mPI.Cat*’) to stratify patients based on mPI values above (+) or below (-) the sample median. Subsequently, a new Cox survival analysis was performed by applying ‘*mPI.Cat*’ as a covariate. Inside each Kaplan-Meier plot, HR, confidence intervals (95% CI), logrank test and expression p values refer to ‘*mPI.Cat*’+ group.

Supplementary Fig. S7

(**A**) Cox survival analysis was done on the full cohort of TCGA patients and on patients from each Verhaak subtype including FAK, HER3, HER3 pY1289, SRC, SRC pY416 and SRC pY527 for GSf-like RPPA endpoints (simplified mPI model). After calculation of mPI (see methods section) in each class of patients analyzed (full cohort, C, P, M and N subtype), a new binary variable was defined (‘*mPI.Cat*’) to stratify patients based on mPI values above (+) or below (-) the sample median. Subsequently, ‘*mPI.Cat*’ was added as covariate in a new Cox regression analysis and the HR, confidence intervals (95% CI), logrank test and expression p values reported inside each plot refer to ‘*mPI.Cat*’+ group. (**B**) Building of a simplified mPI model for the GSr-like RPPA endpoints using only EGFR, EGFR pY1068, EGFR pY1173, EGFR pY992, RPS6, RPS6 pS235-36, RPS6 pS240-44 did not result in an increased power in prediction of survival if compared to a mPI model comprehensive of all GSr-like RPPA analytes (data not shown). Therefore, based on all GSr-like RPPA endpoints we calculated the mPI on the full cohort of TCGA GBM patients, in the Proneural and in the Mesenchymal subtypes and created a new binary variable (‘*mPI.Cat*’) to stratify patients based on mPI values above (+) or below (-) the sample median. Inclusion of *mPI.Cat*, age and MGMT status as covariates in a new Cox regression model dramatically improved the statistical significance of the ‘mPI.Cat’ variable. Inside each Kaplan-Meier plot the reported expression HR, confidence intervals (95% CI), logrank test and expression P values refer to ‘mPI.Cat’+ group while HR and p values for age and MGMT status are specified.
